# Two new species and two new country records of the giant pill-millipede genus *Zephronia* Gray, 1832 from Thailand, based on integrative taxonomy (Diplopoda, Sphaerotheriida, Zephroniidae)

**DOI:** 10.3897/zookeys.1294.205518

**Published:** 2026-10-02

**Authors:** Chalintorn Wilaisorn, Ruttapon Srisonchai, Wibhu Kutanan, Nisarat Tungpairojwong

**Affiliations:** 1 Department of Biology, Faculty of Science, Khon Kaen University, Khon Kaen 40002, Thailand Department of Biology, Faculty of Science, Khon Kaen University Khon Kaen Thailand; 2 Department of Biology, Faculty of Science, Naresuan University, Phitsanulok 65000, Thailand Department of Biology, Faculty of Science, Naresuan University Phitsanulok Thailand

**Keywords:** Biodiversity, DNA barcoding, Myriapoda, Southeast Asia, taxonomy

## Abstract

An integrative taxonomic study combining morphological and molecular data revealed two new species of giant pill-millipedes of the genus *Zephronia* Gray, 1832: *Zephronia tamarindina* Wilaisorn & Srisonchai, **sp. nov**. and *Zephronia triforma* Wilaisorn & Srisonchai, **sp. nov**., both endemic to provinces in the upper central region of Thailand. In addition, *Zephronia dawydoffi* Attems, 1953 and *Zephronia laotica* Wesener, 2019 are recorded from Thailand for the first time. The new species exhibit distinctive colouration patterns, and scanning electron microscopy revealed unique morphological characters for each taxon. Analysis of the 658 bp DNA barcoding region of the mitochondrial *COI* gene showed interspecific genetic distances ranging from 8.68–21.92% for *Z. dawydoffi*, 12.79–22.98% for *Z. laotica*, 10.81–22.07% for *Z. tamarindina***sp. nov**., and 10.81–22.83% for *Z. triforma***sp. nov**., in comparison with closely related species from Thailand, Vietnam, Laos, and Cambodia. Phylogenetic analyses placed all four species within the *Zephronia* s.s. clade, characterised by the Tömösváry’s organ located outside the antennal groove. However, relationships among other clades remained unresolved due to interspersion with species of *Cryxus* Leach, 1814, *Prionobelum* Verhoeff, 1924, and *Sphaerobelum* Verhoeff, 1924. *Zephronia* was recovered as non-monophyletic, indicating that the current generic limits within Zephroniinae require reassessment using multi-locus data. These results expand our understanding of giant pill-millipede diversity and highlight the upper central Thai region and Southeast Asia more broadly as a significant biodiversity hotspot.

## Introduction

The order Sphaerotheriida with their family Zephroniidae are notable for their heavily sclerotised exoskeleton and remarkable ability to roll into a ball-like shape when threatened (Enghoff et al. 2015). The genus *Zephronia* Gray, 1832 is among the most species-rich groups of giant pill-millipedes. With more than 50 described species distributed across Cambodia, China (including Hong Kong), India, Indonesia, Laos, Malaysia, Myanmar, Nepal, Singapore, Thailand, and Vietnam, *Zephronia* exemplifies the biological diversity and regional richness of Southeast Asia (Wesener 2016; Semenyuk et al. 2018; Wesener 2019; Semenyuk et al. 2020; Likhitrakarn et al. 2021; Rosenmejer et al. 2021; Srisonchai et al. 2021, 2024; Srimongkol et al. 2026). Morphologically diverse and ecologically specialised, *Zephronia* forms an integral part of the region’s diplopod fauna. However, despite recent taxonomic revisions and field surveys, the genus remains poorly documented in several biogeographically important areas, particularly in mainland Southeast Asia, including Thailand.

Thailand, situated within the Indo-Burma biodiversity hotspot, harbours a remarkable diversity of millipedes (Diplopoda), with approximately 287 recorded species, a continuously growing number as new discoveries are made (Likhitrakarn et al. 2023; Srisonchai et al. 2023; Donworth and Wesener 2024; Srisonchai et al. 2024; Srikampha et al. 2025; Srimongkol et al. 2026). Most Thai millipede species have been recorded from the central area near the Chao Phraya River Basin, which is considered a major centre of biodiversity in Thailand. Among Thai giant pill-millipedes, only the family Zephroniidae has been documented, represented by three genera: *Sphaerobelum* Verhoeff, 1924, *Prionobelum* Verhoeff, 1924, and *Zephronia* Gray, 1832 (Srisonchai et al. 2023; Donworth and Wesener 2024).

Recent targeted surveys have discovered many new species of the genus *Zephronia*. Since 2021, the interest in Thai giant pill-millipedes has surged, leading to the recognition of up to 14 *Zephronia* species, viz., *Z. chantaburiensis* Srisonchai & Wesener, 2024, *Z. chrysomallos* Bhansali & Wesener, 2022, *Z. enghoffi* Srisonchai & Likhitrakarn, 2021, *Z. erawani* Bhansali & Wesener, 2022, *Z. golovatchi* Srisonchai & Likhitrakarn, 2021, *Z. lannaensis* Likhitrakarn & Golovatch, 2021, *Z. macula* Srisonchai & Wesener, 2024, *Z. ovalis* Gray, 1832, *Z. panhai* Srisonchai & Likhitrakarn, 2021, *Z. phrain* Likhitrakarn & Golovatch, 2021, *Z. siamensis* Hirst, 1907, *Z. sukhothaiensis* Srimongkol & Srisonchai, 2026, *Z. tratensis* Srimongkol & Srisonchai, 2026, and *Z. viridisoma* Rosenmejer & Wesener, 2021. This growing list underscores the remarkable morphological diversity of *Zephronia* in Thailand. However, as with other genera in the Zephroniidae, the taxonomic research on *Zephronia* remains challenging due to limited genetic data and unclear evolutionary relationships (Wesener 2019).

DNA barcoding, based on a fragment of the mitochondrial cytochrome *c* oxidase subunit I (*COI*) gene, has become a powerful molecular tool for millipede species identification. This method enables the rapid and reliable detection of genetic divergence, even among morphologically similar taxa, which is particularly valuable for groups with cryptic species or subtle diagnostic traits. When combined with morphological data, DNA barcoding provides a robust framework for resolving taxonomic uncertainties, verifying species boundaries, and uncovering hidden diversity. Such integrative approaches have proven especially effective for species delimitation for the giant pill-millipede genus *Zephronia* (Wesener 2019; Rosenmejer et al. 2021; Bhansali and Wesener 2022; Zhao et al. 2022; Srisonchai et al. 2024; Srimongkol et al. 2026).

Continuous field surveys and taxonomic workshops have resulted in the accumulation of extensive material over time, from which the specimens examined in this study were obtained. Examination of four morphologically distinct groups of giant pill-millipedes collected during field surveys in 2022–2024 revealed both previously undescribed species and new country records. These findings highlight the rich but still incompletely documented diversity of *Zephronia* in Thailand, particularly in underexplored habitats where endemism is likely high. In this study, we formally describe two new *Zephronia* species and report the first records of *Z. dawydoffi* Attems, 1953 and *Z. laotica* Wesener, 2019 in the country. To ensure accurate identification and resolve potential taxonomic ambiguities, we employed an integrative taxonomic approach, combining detailed morphological examination with molecular analysis based on mitochondrial *COI* sequences.

## Materials and methods

### Specimen collections

The sampling was conducted across the upper central and northeastern regions of Thailand, as well as adjacent areas between 2022 and 2024. All specimens were collected by hand, searching at ground level, beneath leaf litter, and within decomposed logs across a variety of habitats, including agricultural fields, plantation forests, and limestone mountains. Live specimens were photographed *in situ* using a Canon 90D digital camera equipped with a Canon EF 100 mm f/2.8 Macro USM lens. Specimens were euthanised in accordance following the guidelines of the American Veterinary Medical Association (2020). The research protocol was approved by the Institutional Animal Care and Use Committee of Khon Kaen University (No. IACUC-KKU-137/67). After photography, all individuals were preserved in 95% ethanol. Geographic coordinates were recorded on site for each collection locality.

### Maps

The map was generated using a background image from Simplemappr (Shorthouse 2010) and subsequently edited in Adobe Photoshop 2025.

### Morphological identifications and descriptions

A total of 290 specimens were examined under a stereomicroscope. Species identification was based on morphological characters and comparisons with descriptions from the previous literature, as well as with type specimens of other Thai congeners. Morphological terminology and descriptive formats followed those used by Golovatch et al. (2012), Wesener (2019), Semenyuk et al. (2018), and Srisonchai et al. (2024) for morphological descriptions.

Type specimens, including holotypes and paratypes, were deposited in the following institutions: Chulalongkorn University Museum of Zoology, Thailand (**CUMZ**), Khon Kaen University Museum of Zoology, Thailand (invertebrate collections) (**MZKKU**), Natural History Museum of Denmark, Denmark (**NHMD**), Natural History Museum, United Kingdom (**NHMUK**), Naturhistorisches Museum Wien, Austria (**NHMW**) and Zoological Research Museum Koenig, Germany (**ZFMK**).

Because the present study focuses on the description and redescription of four species rather than a comprehensive revision of the genus *Zephronia*, no general description of the genus is provided. Instead, each species is described separately in full to facilitate direct comparisons and to ensure clarity in species delimitations. Such a format is commonly adopted in taxonomic works dealing with a limited number of new species, particularly when the primary objective is species diagnoses and identification rather than a generic revision.

### List of abbreviations

**3it** 3-combed inner tooth of mandible,

**bu** bursa of vulva,

**co** condylus of mandible,

**Cp** central pad of gnathochilarium,

**cr-t** crenulated teeth on telopods,

**ct** central tooth,

**cx** coxa,

**et** external tooth of epipharynx,

**Et** external tooth of mandibular gnathal lobe,

**fe** femur,

**Ia** inner area of mandible,

**il** incisura lateralis of the head,

**ip** inner palpi of gnathochilarium,

**ll** lamellae lingulales of gnathochilarium,

**me** mentum of gnathochilarium,

**ml** membranous lobe,

**mp** molar plate of mandible,

**Mp** membranous process

**op** operculum of vulva,

**pl** pectinate lamellae of mandible,

**po** postfemur,

**pre** prefemur,

**rdg** femoral ridge,

**rsp** row of spines,

**sc** sensory cone,

**scl-s** sclerotised spots,

**st** stipites of gnathochilarium,

**st-pl** stigmatic plate,

**syn-cx** syncoxite,

**ta** tarsus,

**ti** tibia.

### Scanning electron microscopy (SEM)

The specimens were examined and dissected under a stereo microscope. Morphological structures were dried in the desiccator for 24 hours, mounted on aluminium stubs, and coated with gold. Samples were then photographed under a high vacuum in a JEOL JSM-5410 LV scanning electron microscope at the Faculty of Science, Khon Kaen University. All figures were subsequently assembled and refined in Adobe Photoshop 2025.

### DNA extraction and PCR amplifications

The genomic DNA was extracted from 27 specimens, comprising four and eight specimens of the two new species, ten specimens of *Z. dawydoffi*, and foive specimens of *Z. laotica*. Extractions were performed from the leg tissue using proteinase K digestion and following the manufacturer’s instructions of Vivantis GF-1 Nucleic Acid Extraction Kits. PCR amplification of the 658 bp barcoding region of the mitochondrial cytochrome *c* oxidase subunit I (*COI*) gene using the universal primers — LCO-1490 (5’-GGT CAA CAA ATC ATA AAG ATA TTG G-3’) and HCO-2198 (5’-TAA ACT TCA GGG TGA CCA AAA AAT CA-3’) (Folmer et al. 1994). The thermal cycling program began with one cycle at 94 °C for 3 min; followed by 35 cycles of denaturation at 94 °C for 30 sec, annealing at 45 or 47 °C for 40 sec, and extension at 72 °C for 1 min, and a final extension step at 72 °C for 5 min. The PCR success was checked using 1.5% agarose gel electrophoresis and then observed under UV transillumination. The PCR products were purified and sequenced, using the same primers as those used for PCR amplification, at ATGC Co., Ltd. (Thailand).

### DNA alignment and *COI* barcoding tree reconstruction

A total of 124 sequences were analysed, including 27 sequences generated in this study and 97 sequences retrieved from public databases representing all recorded *Zephronia* species. Outgroups included *Arthrosphaera brandtii* (Humbert, 1865), *Cryxus ovalis* (Linnaeus, 1758), *Epicyliosoma* sp., *Glomeris marginata* (Villers, 1789), *Prionobelum* spp., *Sphaerobelum* spp., and *Sphaeromimus splendidus* Wesener & Sierwald, 2005. The newly generated sequences were then deposited in GenBank (Table 1).

**Table 1.** List of species analysed in this study with GenBank accession numbers.

	**Species**	***COI* accession number**	**Voucher code**	**Locality**	**Reference**
**Ingroups**					
1	*Zephronia chantaburiensis* Srisonchai & Wesener, 2024	PP754582	CUMZ (M47)	Thailand, Chantaburi Province, Tha Mai District, Wat Khao Sukim	Srisonchai et al. 2024
2	*Zephronia chantaburiensis* Srisonchai & Wesener, 2024	PP754583	CUMZ (M48)	Thailand, Chantaburi Province, Tha Mai District, Wat Khao Sukim	Srisonchai et al. 2024
3	*Zephronia chrysomallos* Bhansali & Wesener, 2022	OM509649	ZFMK MYR8826	Thailand, Kanchanaburi Province, Sai Yok District, Sai Yok Noi Waterfall	Bhansali and Wesener 2022
4	*Zephronia dawydoffi* Attems, 1953	MK330971	ZFMK Myr4504	N/A	Wesener 2019
5	*Zephronia dawydoffi* Attems, 1953	PX737475	MZKKU (BRM06)	Thailand, Buri Ram Province, Khu Mueang District, Dong Kheng rubber plantation	This study
6	*Zephronia dawydoffi* Attems, 1953	PX737476	MZKKU (BRM10)	Thailand, Buri Ram Province, Khu Mueang District, Dong Kheng rubber plantation	This study
7	*Zephronia dawydoffi* Attems, 1953	PX737477	MZKKU (BRM17)	Thailand, Buri Ram Province, Khu Mueang District, Non Somboon Monastery	This study
8	*Zephronia dawydoffi* Attems, 1953	PX737478	MZKKU (BRM24)	Thailand, Buri Ram Province, Khu Mueang District, Non Somboon Monastery	This study
9	*Zephronia dawydoffi* Attems, 1953	PX737479	MZKKU (DBR01)	Thailand, Buri Ram Province, Mueang District, Khao Kradong	This study
10	*Zephronia dawydoffi* Attems, 1953	PX737480	MZKKU (DBR02)	Thailand, Buri Ram Province, Mueang District, Khao Kradong	This study
11	*Zephronia dawydoffi* Attems, 1953	PX737481	MZKKU (DBR04)	Thailand, Buri Ram Province, Mueang District, Ban Khok Ta Sing Waterfall	This study
12	*Zephronia dawydoffi* Attems, 1953	PX737482	MZKKU (DBR06)	Thailand, Buri Ram Province, Mueang District, Ban Khok Ta Sing Waterfall	This study
13	*Zephronia dawydoffi* Attems, 1953	PX737483	MZKKU (KKN12)	Thailand, Khon Kaen Province, Mueang District, Thung Setthi Temple	This study
14	*Zephronia dawydoffi* Attems, 1953	PX737484	MZKKU (KKN20)	Thailand, Khon Kaen Province, Mueang District, Thung Setthi Temple	This study
15	*Zephronia erawani* Bhansali & Wesener, 2022	OM509650	NHMD K56x9	Thailand, Kanchanaburi Province, Si Sawat District, 50 km W of Kanchanaburi, Erawan Waterfall	Bhansali and Wesener 2022
16	*Zephronia golovatchi* Srisonchai, Sutcharit & Likhitrakarn, 2021	OM509646	ZFMK MYR6262	Thailand, Nakhon Ratchasima Province, Pak Chong District	Bhansali and Wesener 2022
17	*Zephronia golovatchi* Srisonchai, Sutcharit & Likhitrakarn, 2021	OM509647	ZFMK K53	Thailand, Nakhon Nayok Province, Khao Yai National Park	Bhansali and Wesener 2022
18	*Zephronia hui* Liu & Wesener, 2022	OP339790	SCAU YGM03	China, Guizhou, Tongren City, Jiangkou County, Taiping Town, Yamugou Scenic Area	Zhao et al. 2022
19	*Zephronia lannaensis* Likhitrakarn & Golovatch, 2021	OM509629	ZFMK MYR3498	Thailand, Chiangmai Province, Mueang District, Doi Suthep	Bhansali and Wesener 2022
20	*Zephronia lannaensis* Likhitrakarn & Golovatch, 2021	OM509630	ZFMK MYR3501	Thailand, Chiangmai Province, Mae Rim District, Traidhos School Campus	Bhansali and Wesener 2022
21	*Zephronia lannaensis* Likhitrakarn & Golovatch, 2021	OM509631	ZFMK MYR4911	Thailand, Chiangmai Province, Mae Rim District, Mae Sa Valley	Bhansali and Wesener 2022
22	*Zephronia lannaensis* Likhitrakarn & Golovatch, 2021	OM509632	NHMD K57B	Thailand, Chiangmai Province, Mueang District, Doi Suthep, Me Sa Waterfall	Bhansali and Wesener 2022
23	*Zephronia lannaensis* Likhitrakarn & Golovatch, 2021	OM509633	MHNG 3B	Thailand, Chiangmai Province, Mueang District, Doi Suthep	Bhansali and Wesener 2022
24	*Zephronia laotica* Wesener, 2019	MK330977	ZFMK Myr3502	Laos, Champasak Province, east of Mekong, Garden of Erawan Riverside Hotel	Wesener 2019
25	*Zephronia laotica* Wesener, 2019	PX737485	MZKKU (SSK01)	Thailand, Si Sa Ket Province, Khun Han District, Mae Lamoon Durian plantation	This study
26	*Zephronia laotica* Wesener, 2019	PX737486	MZKKU (SSK03)	Thailand, Si Sa Ket Province, Khun Han District, Mae Lamoon Durian plantation	This study
27	*Zephronia laotica* Wesener, 2019	PX737487	MZKKU (SSK14)	Thailand, Si Sa Ket Province, Khun Han District, Kantrom Tai Temple	This study
28	*Zephronia laotica* Wesener, 2019	PX737488	MZKKU (SSK18)	Thailand, Si Sa Ket Province, Khun Han District, Kantrom Tai Temple	This study
29	*Zephronia laotica* Wesener, 2019	PX737489	MZKKU (SSK25)	Thailand, Si Sa Ket Province, Khun Han District, Ta Nuan Rubber plantation	This study
30	*Zephronia macula* Srisonchai & Wesener, 2024	PP754589	CUMZ (M54)	Thailand, Sra Kaeo Province, Mueang Sra Kaeo District, Wat Tham Khao Maka	Srisonchai et al. 2024
31	*Zephronia macula* Srisonchai & Wesener, 2024	PP754590	CUMZ (M55)	Thailand, Sra Kaeo Province, Mueang Sra Kaeo District, Wat Tham Khao Maka	Srisonchai et al. 2024
32	*Zephronia macula* Srisonchai & Wesener, 2024	PP754585	CUMZ- MYR 0015 (M418)	Thailand, Chantaburi Province, Khlung District, Thaeo Khlong Khlung Monastery	Srisonchai et al. 2024
33	*Zephronia macula* Srisonchai & Wesener, 2024	PP754587	CUMZ- MYR 0015 (M422)	Thailand, Chantaburi Province, Tha Mai District, Wat Khao Sukim	Srisonchai et al. 2024
34	*Zephronia macula* Srisonchai & Wesener, 2024	PX526095	MZKKU (M400)	Thailand, Chantaburi Province, Tha Mai District, Wat Khao Sukim	Srimongkol et al. 2026
35	*Zephronia macula* Srisonchai & Wesener, 2024	PX526097	MZKKU (M437)	Thailand, Chachoengsao Province, Phanom Sarakham District, Wat Khao Hin Sorn	Srimongkol et al. 2026
36	*Zephronia macula* Srisonchai & Wesener, 2024	PX526098	MZKKU (M438)	Thailand, Rayong Province, Khao Chamao District, Wat Tham Suwan Phupha	Srimongkol et al. 2026
37	*Zephronia macula* Srisonchai & Wesener, 2024	PX526100	MZKKU (M457)	Thailand, Sa Kaeo Province, Khao Chakan District, Wat Tham Khao Chan	Srimongkol et al. 2026
38	*Zephronia medongensis* Zhao & Liu, 2022	OP339793	SCAU XZ01	China, Xizang Autonomous Region, Medog County	Zhao et al. 2022
39	*Zephronia ovalis* Gray, 1832	JX486068	ZFMK Myr 0832	Vietnam, Dong Nai Province, Cat Tien National Park	Golovatch et al. 2012
40	*Zephronia ovalis* Gray, 1832	PX526103	MZKKU (M180)	Thailand, Si Sa Ket Province, Khunhan District, Huai Chan Waterfall	Srimongkol et al. 2026
41	*Zephronia ovalis* Gray, 1832	PX526104	MZKKU (M181)	Thailand, Si Sa Ket Province, Khunhan District, Huai Chan Waterfall	Srimongkol et al. 2026
42	*Zephronia ovalis* Gray, 1832	PX526105	MZKKU (M204)	Thailand, Si Sa Ket Province, Khunhan District, Huai Chan Waterfall	Srimongkol et al. 2026
43	*Zephronia panhai* Srisonchai, Sutcharit & Likhitrakarn, 2021	OM509643	ZFMK MYR 8118	Thailand, Ratchaburi Province, Ratchaburi-Photharam Districts	Bhansali and Wesener 2022
44	*Zephronia panhai* Srisonchai, Sutcharit & Likhitrakarn, 2021	OM509644	MHNG 3A	Thailand, Ratchaburi Province, Chom Bueang District, Tham Kao Bin Forest Park	Bhansali and Wesener 2022
45	*Zephronia panhai* Srisonchai, Sutcharit & Likhitrakarn, 2021	OM509645	ZFMK MYR 8116	Thailand, Ratchaburi Province, Ratchaburi-Photharam Districts	Bhansali and Wesener 2022
46	*Zephronia phrain* Likhitrakarn & Golovatch, 2021	OM509634	ZFMK MYR 3499	Thailand, Chiang Mai Province, Chiang Dao District, Padeng Lodge	Bhansali and Wesener 2022
47	*Zephronia phrain* Likhitrakarn & Golovatch, 2021	OM509635	MYR3500	Thailand, Chiangmai Province, Mueang District, Doi Suthep	Bhansali and Wesener 2022
48	*Zephronia phrain* Likhitrakarn & Golovatch, 2021	OM509636	SMF	Thailand, Chiang Mai Province, Chiang Dao District, Tham Houay Luk	Bhansali and Wesener 2022
49	*Zephronia phrain* Likhitrakarn & Golovatch, 2021	OM509637	SMF	Thailand, Chiang Mai Province, Chiang Dao District, Doi Chiang Dao, Ma Lee’s Resort	Bhansali and Wesener 2022
50	*Zephronia phrain* Likhitrakarn & Golovatch, 2021	OM509638	SMF	Thailand, Chiang Mai Province, Chai Prakan District, Tham Ngam	Bhansali and Wesener 2022
51	*Zephronia phrain* Likhitrakarn & Golovatch, 2021	OM509639	ZFMK MYR 4907	Thailand, Chiang Mai Province, Chiang Dao	Bhansali and Wesener 2022
52	*Zephronia phrain* Likhitrakarn & Golovatch, 2021	OM509640	MHNG 5G	Thailand, Lamphun Province, Mae Tha District, Doi Khuntan National Park	Bhansali and Wesener 2022
53	*Zephronia phrain* Likhitrakarn & Golovatch, 2021	OM509641	MHNG 5I	Thailand, Chiang Mai Province, Chiang Dao District, Doi Chiang Dao	Bhansali and Wesener 2022
54	*Zephronia phrain* Likhitrakarn & Golovatch, 2021	OM509642	NHMD K35	Thailand, Chiang Mai Province, Ban Musue	Bhansali and Wesener 2022
55	*Zephronia siamensis* Hirst, 1907 (unverified)	JX486067.2	FMNH-INS-72669	Thailand, Chonburi Province, Sichang District, Koh Sichang	Golovatch et al. 2012
56	*Zephronia siamensis* Hirst, 1907	OR530089	CUMZ	Thailand, Chonburi Province, Sichang District, Koh Sichang	Srisonchai et al. 2023
57	*Zephronia siamensis* Hirst, 1907	PP754592	CUMZ (M455)	Thailand, Chachoengsao Province, Phanom Sarakham District, Wat Khao Hin Sorn	Srisonchai et al. 2024
58	*Zephronia siamensis* Hirst, 1907	PP754591	CUMZ (D397)	Thailand, Srakaeo Province, Khao Chakan District, Wat Tham Khao Chan	Srisonchai et al. 2024
59	*Zephronia* sp. (K45 Aow Noi Temple)	MW898741	NHMD K55	Thailand, Prachuap Kiri Khan Province, Mueang District, Aow Noi Temple	Rosenmejer et al. 2021
60	*Zephronia* sp. (Aow Noi Temple)	MW898742	ZFMK MYR 8787	Thailand, Prachuap Kiri Khan Province, Mueang District, Aow Noi Temple	Rosenmejer et al. 2021
61	*Zephronia sukhothaiensis* Srimongkol & Srisonchai, 2026	PX526107	MZKKU (L43)	Thailand, Sukhothai Province, Ban Dan Lan Hoi District, Wat Khao Fang	Srimongkol et al. 2026
62	*Zephronia sukhothaiensis* Srimongkol & Srisonchai, 2026	PX526108	MZKKU (M323)	Thailand, Sukhothai Province, Ban Dan Lan Hoi District, Wat Khao Fang	Srimongkol et al. 2026
63	*Zephronia sukhothaiensis* Srimongkol & Srisonchai, 2026	PX526110	MZKKU (M325)	Thailand, Sukhothai Province, Ban Dan Lan Hoi District, Wat Khao Fang	Srimongkol et al. 2026
64	*Zephronia tamarindina* sp. nov.	PX737490	MZKKU (L83)	Thailand, Phetchabun Province, Bueng Sam Phan District, Nimit Sat Cha Dhum Temple	This study
65	*Zephronia tamarindina* sp. nov.	PX737491	MZKKU (L84)	Thailand, Phetchabun Province, Bueng Sam Phan District, Nimit Sat Cha Dhum Temple	This study
66	*Zephronia tamarindina* sp. nov.	PX737492	MZKKU (UPNB01)	Thailand, Phetchabun Province, Bueng Sam Phan District, Nimit Sat Cha Dhum Temple	This study
67	*Zephronia tamarindina* sp. nov.	PX737493	MZKKU (UPNB02)	Thailand, Phetchabun Province, Bueng Sam Phan District, Nimit Sat Cha Dhum Temple	This study
68	*Zephronia tratensis* Srimongkol & Srisonchai, 2026	PX526112	MZKKU (M415)	Thailand, Trat Province, Mueang District, Khao Khun Tham Monastery	Srimongkol et al. 2026
69	*Zephronia tratensis* Srimongkol & Srisonchai, 2026	PX526113	MZKKU (M416)	Thailand, Trat Province, Mueang District, Khao Khun Tham Monastery	Srimongkol et al. 2026
70	*Zephronia triforma* sp. nov.	PX737494	MZKKU (L60)	Thailand, Nakhon Sawan Province, Takhli District, Buddhawajana Dhamma Retreat	This study
71	*Zephronia triforma* sp. nov.	PX737495	MZKKU (L61)	Thailand, Nakhon Sawan Province, Takhli District, Buddhawajana Dhamma Retreat	This study
72	*Zephronia triforma* sp. nov.	PX737498	MZKKU (UNSN01)	Thailand, Nakhon Sawan Province, Takhli District, Buddhawajana Dhamma Retreat	This study
73	*Zephronia triforma* sp. nov.	PX737499	MZKKU (UNSN02)	Thailand, Nakhon Sawan Province, Takhli District, Buddhawajana Dhamma Retreat	This study
74	*Zephronia triforma* sp. nov.	PX737496	MZKKU (L227)	Thailand, Phitsanulok Province, Wat Bot District, Ban Nam Kob	This study
75	*Zephronia triforma* sp. nov.	PX737497	MZKKU (L229)	Thailand, Phitsanulok Province, Wat Bot District, Ban Nam Kob	This study
76	*Zephronia triforma* sp. nov.	PX737500	MZKKU (UPLK07)	Thailand, Phitsanulok Province, Wat Bot District, Khao Bo Ma Viewpoint	This study
77	*Zephronia triforma* sp. nov.	PX737501	MZKKU (UPLK12)	Thailand, Phitsanulok Province, Wat Bot District, Khao Bo Ma Viewpoint	This study
78	*Zephronia viridisoma* Rosenmejer & Wesener, 2021	MW898739	NHMD 621695	Thailand, Nakhon Si Thammarat Province, Sichon District, Khao Lark Waterfall	Rosenmejer et al. 2021
79	*Zephronia zhouae* Zhao & Liu, 2022	OP339794	SCAU YN02	China, Yunnan Province, Diqing Tibetan Autonomous Prefecture, Weixi County, Laowo Village	Rosenmejer et al. 2021
**Outgroups**					
80	*S. aesculus* Rosenmejer & Wesener, 2021	MW898737	NHMD 621693	Thailand, Phuket Province, Kathu District, Forest	Rosenmejer et al. 2021
81	*S. cf. aesculus* Rosenmejer & Wesener, 2021	MW898738	NHMD 621694	Thailand, Nakhon Si Thammarat Province, Khao Luang NP	Rosenmejer et al. 2021
82	*S. benquii* Liu & Wesener, 2022	OP339792	SCAU MMY01	China, Guizhou, Tongren City, Jiangkou County, Guanhe Town, Guanhe Village, Maomaoyan	Zhao et al. 2022
83	*S. bolavensis* Wesener, 2019	MK330982	MHNG LT-10/24	Laos, Champasak Province, Bolaven Plateau, 3 km S of Ban Nong Luang, Tad Kameud	Wesener 2019
84	*S. denticulatum* Wesener, 2019	MK330984	MHNG LT-10/12	Laos, Oudomxai Province, ca 3 km E of Tad Lak 11, SE of Oudomxai city	Wesener 2019
85	*S. huzhengkuni* Zhao, Yu & Liu, 2020	MT657327	SCAU SP02	China, Guizhou Province, Tongren City, Fanjingshan National Nature Reserve	Zhao et al. 2020
86	*S. huzhengkuni* Zhao, Yu & Liu, 2020	MT657328	SCAU SP03	China, Guizhou Province, Tongren City, Fanjingshan National Nature Reserve	Zhao et al. 2020
87	*S. lachneeis* Wesener, 2019	MK330983	MHNG LT-10/12	Laos, Oudomxai Province, ca 3 km E of Tad Lak 11, SE of Oudomxai city	Wesener 2019
88	*S. laoticum* Wesener, 2019	MK330975	SMF	Laos, Vientiane Province, Vang Vieng	Wesener 2019
89	*S. meridionalis* Bhansali & Wesener, 2022	OM509648	MHNG 4B-2	Thailand, Yala Province, Bannang Sata District, Bang Lang National Park, near Than To Waterfall	Bhansali and Wesener 2022
90	*S. nigrum* Wesener, 2019	MK330976	SMF	Laos, Champasak Province, Muang Bachieng, Ban Lak 35, Tad Etu	Wesener 2019
91	*S. nonghinensis* Srikampha & Srisonchai, 2025	PQ838982	MZKKU (A35)	Thailand, Loei Province, Nong Hin District, Suan Hin Pha Ngam (Hin Pha Ngam Park)	Srikampha et al. 2025
92	*S. nonghinensis* Srikampha & Srisonchai, 2025	PQ838983	MZKKU (A36)	Thailand, Loei Province, Nong Hin District, Suan Hin Pha Ngam (Hin Pha Ngam Park)	Srikampha et al. 2025
93	*S. nonghinensis* Srikampha & Srisonchai, 2025	PQ838984	MZKKU (E28)	Thailand, Loei Province, Nong Hin District, Suan Hin Pha Ngam (Hin Pha Ngam Park)	Srikampha et al. 2025
94	*S. nonghinensis* Srikampha & Srisonchai, 2025	PQ838986	MZKKU (M237)	Thailand, Loei Province, Nong Hin District, Suan Hin Pha Ngam (Hin Pha Ngam Park)	Srikampha et al. 2025
95	*S. onyx* Srikampha & Srisonchai, 2025	PQ838987	MZKKU (M211)	Thailand, Buang Kan Province, Seka District, Wat Tham Chum Chang (Nam Yaad Clift)	Srikampha et al. 2025
96	*S. peterjaegeri* Wesener, 2019	MK330972	SMF SD553	Laos, Luang Prabang Province, SE Luang Prabang, Nam Khan, Ban Pak Bak, Houay Kho	Wesener 2019
97	*S. petramurum* Srikampha & Srisonchai, 2025	PQ838989	MZKKU (E5)	Thailand, Khon Kaen Province, Phu Pha Man District, Ban Khao Wong	Srikampha et al. 2025
98	*S. petramurum* Srikampha & Srisonchai, 2025	PQ838991	MZKKU (L128)	Thailand, Khon Kaen Province, Phu Pha Man District, Ban Khao Wong	Srikampha et al. 2025
99	*S. petramurum* Srikampha & Srisonchai, 2025	PQ838992	MZKKU (L6)	Thailand, Chaiyaphum Province, Khon San District, Mortagek Monastery	Srikampha et al. 2025
100	*S. petramurum* Srikampha & Srisonchai, 2025	PQ838993	MZKKU (M217)	Thailand, Khon Kaen Province, Phu Pha Man District, Ban Khao Wong	Srikampha et al. 2025
101	*S. phouloei* Wesener, 2019	MK330974	ZMUC00040257	Laos, Houaphan Province, Phou Loei	Wesener 2019
102	*S. schwendingeri* Wesener, 2019	MK330978	MHNG LT-10/03	Laos, Vientiane Province, trail to Tham Pou Kham, W. of Vang Vieng	Wesener 2019
103	*S. schwendingeri* Wesener, 2019	MK330981	SMF	Laos, Vientiane Province, Vang Vieng, Tham Pou Kham	Wesener 2019
104	*Sphaerobelum* sp. L07	MK330979	ZMUC00040261	Laos, Khammouane Province, Ban Khounkham [Khun Kham] (Nahin)	Wesener 2019
105	*Sphaerobelum* sp. L10	MK330980	SMF	Laos, Vientiane Province, Vang Vieng, W. of Nam Song, Tham Nam Or Khem	Wesener 2019
106	*S. spinatum* Wesener, 2019	MK330973	ZMUC00040258	Laos, Vientiane Province, Phou Khao Khouay	Wesener 2019
107	*S. truncatum* Wongthamwanich, 2012	JN885184	FMNH-INS 0000 072 674	Thailand, Nan Province, Song Khwae District, Na Rai Luang Subdistrict, Pang Hi Village	Wongthamwanich et al. 2012
108	*S. tujiaphilum* Zhao & Liu, 2022	OP339785	SCAU BHS01	China, Guizhou, Tongren City, Jiangkou County, Taiping Town, Baiheshan Village	Zhao et al. 2022
109	*S. tujiaphilum* Zhao & Liu, 2022	OP339787	SCAU JXT02	China, Guizhou, Tongren City, Jiangkou County, Taiping Town, Jiang-xitun Village	Zhao et al. 2022
110	*S. tujiaphilum* Zhao & Liu, 2022	OP339788	SCAU SD03	China, Guizhou, Tongren City, Jiangkou County, Guanhe Town, Sidu Village	Zhao et al. 2022
111	*S. tujiaphilum* Zhao & Liu, 2022	OP339789	SCAU DW01	China, Guizhou, Tongren City, Jiangkou County, Dewang Town, Xiaobang Village	Zhao et al. 2022
112	*S. turcosa Srisonchai & Pimvichai*, *2023*	OR530087	CUMZ-Zeph0012	Thailand, Loei Province, Mueang Loei District, Phu Pha Lom Forest Park	Srisonchai et al. 2023
113	*S. turcosa Srisonchai & Pimvichai*, *2023*	OR530087	CUMZ-Zeph0012	Thailand, Loei Province, Mueang Loei District, Phu Pha Lom Forest Park	Srisonchai et al. 2023
114	*Arthrosphaera brandtii* (Humbert, 1865)	FJ409915	FMNH-INS 8650	Tanzania, Usambara hills	Wesener et al. 2010
115	*Cryxus ovalis* (Linnaeus, 1758) (unverified)	JX486069.2	ZFMK MYR0824	Vietnam, Dong Nai Province, Cat Tien National Park	Golovatch et al. 2012
116	*Epicyliosoma* sp.	AF218270	NA	NA	Edgecombe and Giribet 2004
117	*Glomeris marginata* (Villers, 1789)	FJ409909	ZFMK Myr009	Germany, Bonn, Venusberg	Wesener et al. 2010
118	*P. inthanonense* Donworth & Wesener, 2024	PP297645	MHNG 4E-2	Thailand, Chiang Mai Province, Chom Thong District, Doi Inthanon National Park	Donworth and Wesener 2024
119	*P. inthanonense* Donworth & Wesener, 2024	PP297646	MHNG 7A	Thailand, Chiang Mai Province, Chom Thong District, Doi Inthanon National Park	Donworth and Wesener 2024
120	*P. naevium* Donworth & Wesener, 2024	PP297647	MHNG 4B-1	Thailand, Yala Province, Than To District, Bang Lang National Park, Than To Waterfall	Donworth and Wesener 2024
121	*P. naevium* Donworth & Wesener, 2024	PP297648	NHMD 1184671	Thailand, Yala Province, Than To District, Bang Lang National Park	Donworth and Wesener 2024
122	*P. naevium* Donworth & Wesener, 2024	PP297649	NHMD 1184672	Thailand, Yala Province, Than To District, Bang Lang National Park	Donworth and Wesener 2024
123	*P. naevium* Donworth & Wesener, 2024	PP297650	NHMD 1184673	Thailand, Yala Province, Than To District, Bang Lang National Park	Donworth and Wesener 2024
124	*Sphaeromimus splendidus* Wesener & Sierwald, 2005	FJ409917	FMMC-INS 6702	Madagascar, Sainte Luce S9	Wesener et al. 2010

The chromatograms of all sequences were examined, aligned, and trimmed using BioEdit v. 7.0.5.3 (https://bioedit.software.informer.com/download/) (Hall 1999) and MEGA X (https://www.megasoftware.net/) (Kumar et al. 2018). Alignments were checked for stop codons, and missing nucleotides were coded as “N”. The best-fit nucleotide substitution model was calculated using JModelTest2 on XSEDE 2.1.6 (Darriba et al. 2012) via the CIPRES Gateway (Miller et al. 2010) which resulted GTR+I+G as the best model.

*COI* barcoding trees were reconstructed using maximum likelihood (ML) and Bayesian inference (BI) methods. The ML tree was generated using the IQ-tree on XSEDE 1.6.6 (Minh et al. 2020) with the GTR+I+G model and with 10,000 bootstrap replicates. For BI analysis, the dataset was processed in KAKUSAN v. 4.0 (https://kakusan4.software.informer.com/download/#google_vignette) (Tanabe 2011) to select the optimal model, and analyses were run in MrBayes on XSEDE 3.2.7a (Ronquist et al. 2012) via the CIPRES Gateway with a random starting tree and Markov Chain Monte Carlo (MCMC), sampling every 1,000 generations for 50 million generations. Phylogenetic trees were visualised in FigTree v. 1.4.4 (https://tree.bio.ed.ac.uk/software/figtree/) (Rambaut 2018) and finalised in Adobe Illustrator 2025. The BI posterior probabilities (PP) > 95% and ML bootstrap support (BS) > 70% were considered as strong support (Huelsenbeck and Hillis 1993; Larget and Simon 1999).

Interspecific and intraspecific genetic distances were calculated using the uncorrected *p*-distance model in MEGA X (Kumar et al. 2018), based on pairwise comparisons with pairwise deletion of missing data.

## Results

### *COI* sequences annotations and genetic distances

The 658 bp *COI* gene fragments from 124 sequences contained 376 polymorphic sites, 282 conservative sites, and 316 parsimony informative sites. The genetic distances (percentage) between *Zephronia* and other closely related genera were from 6.85–25.27% between *Sphaerobelum* and *Zephronia*, 16.44–23.29% between *Prionobelum* and *Zephronia*, and 18.02–23.38% between *Cryxus* Leach, 1814 and *Zephronia*.

Within *Zephronia* itself, interspecific divergence ranged from 8.68 to 24.20%, with the lowest distance observed between *Z. siamensis* and *Z. dawydoffi*. Pairwise interspecific distances between each taxon and its closest congeners within *Zephronia* were 8.68–21.92% in *Z. dawydoffi*, 12.79–22.98% in *Z. laotica*, 10.81–22.07% in *Z. tamarindina* sp. nov., and 10.81–22.83% in *Z. triforma* sp. nov. The intraspecific genetic distances reached up to 5.18% in *Z. dawydoffi*, 5.95% in *Z. laotica*, 0.61% in *Z. tamarindina* sp. nov., and 5.63% in *Z. triforma* sp. nov., respectively. Notably, *Z. dawydoffi* in this study differed by 3.65–5.18% from the GenBank sequence (MK330971), while *Z. laotica* differed by 4.88–5.95% from the GenBank sequence (MK330977) (see Suppl. material 1).

### Barcoding tree based on *COI*

**Figure 1.**
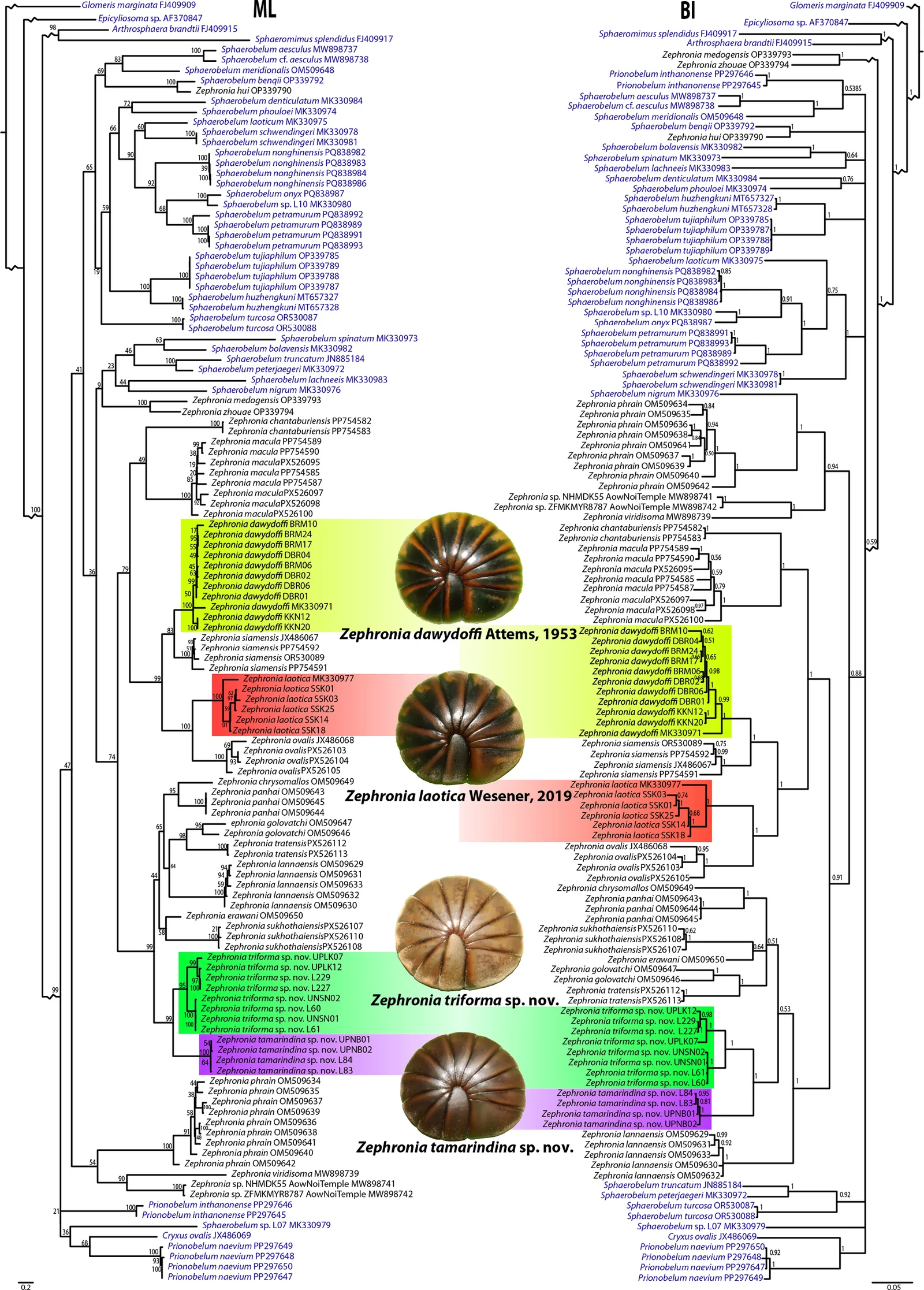
*COI* barcoding trees based on Bayesian Inference (BI) and maximum likelihood (ML) of the genus *Zephronia* and related genera. Numbers on each node represent posterior probabilities (PP) from BI and bootstrap support (BS) from ML. Background colours indicate the two new species (*Zephronia tamarindina* sp. nov. and *Zephronia triforma* sp. nov.), and the two new recorded species from Thailand (*Zephronia dawydoffi* Attems, 1953 and *Zephronia laotica* Wesener, 2019), relating to the photograph of live specimens. The scale bar represents the branch length. Colour of scientific letters: blue = outgroup species, black = *Zephronia* species.

*COI* barcoding tree reconstruction using BI and ML methods produced partially incongruent topologies. However, most interspecific relationships, particularly among *Zephronia* s.s. from Thailand, Laos, Vietnam, and Cambodia, were broadly consistent (Fig. 1 and see list of species in *Zephronia* s.s. below).

Overall, nodal support across deeper branches was generally low, limiting the resolution of relationships among *Zephronia*, *Sphaerobelum*, *Prionobelum*, and *Cryxus*. Both BI and ML trees indicated that *Zephronia* is non-monophyletic. In the BI tree, *Zephronia*, *Sphaerobelum*, *Prionobelum*, and *Cryxus* were intermixed, forming a poorly resolved polytomy with low support (PP = 0.59). Similarly, in the ML, *Zephronia* was divided into four major clades with weak support (BS = 47). While most of the *Zephronia* s.s. clustered together, *Z. phrain*, *Z. viridisoma*, and *Zephronia* sp. (Aow Noi Temple) formed a separate clade. In addition, *Z. hui*, *Z. medogensis*, and *Z. zhouae* were interspersed with *Sphaerobelum* in two distinct clades.

All other *Zephronia* s.s. specimens from Thailand, Laos, Vietnam, and Cambodia, including the newly sequenced *Z. dawydoffi*, *Z. laotica*, *Z. tamarindina* sp. nov., *and Z. triforma* sp. nov., formed a moderately to well-supported monophyletic group in both BI and ML trees (BS = 74, PP = 0.91). Within this clade, the two new species were nested among members of the *Zephronia* s.s. group (*Z. chrysomallos*, *Z. erawani*, *Z. golovatchi*, *Z. lannaensis*, and *Z. panhai*) with strong support (BS = 99, PP = 1.00). *Z. tamarindina* sp. nov. and *Z. triforma* sp. nov. were recovered as sister species (BS = 99, PP = 1.00). *Z. dawydoffi* and *Z. laotica* were grouped together with *Z. chantaburiensis*, *Z. macula*, *Z. ovalis*, and *Z. siamensis* with strong support (BS = 79, PP = 1.00). Sequences of *Z. dawydoffi* and *Z. laotica* from this study clustered with their respective GenBank references (MK330971 of *Z. dawydoffi*: BS = 50, PP = 1.00; MK330977 of *Z. laotica*: BS = 100, PP = 1.00).

### Systematics

**Class Diplopoda de Blainville in Gervais, 1844**

**Order Sphaerotheriida Brandt, 1833**

**Family Zephroniidae Gray, 1843**

**Subfamily Zephroniinae Gray, 1843**

**Tribe Zephroniini Gray, 1843**

#### Genus Zephronia

Taxon classification

Animalia

SphaerotheriidaZephroniidae

Gray, 1832

CCC627CB-CB92-5E64-B3EE-5B2471D9F2A4

##### Type species.

*Zephronia ovalis* Gray, 1832.

##### Species included.

59 species, including the two new species described here.

##### Distribution.

Cambodia, China (including Hong Kong), India (NE), Indonesia, Laos, Malaysia, Myanmar, Nepal, Singapore, Thailand, and Vietnam.

*Zephronia* is one of the most species-rich genera within Zephroniidae, currently comprising 59 described species. Many species remain insufficiently studied, especially type re-examinations are lacking, with many known only from females, particularly those from Myanmar, Borneo, Sumatra, and India. Consequently, the genus is in urgent need of a revision, and some species currently placed in *Zephronia* may be transferred to separate genera in the future.

*Zephronia* differs from other genera of Zephroniinae by the following combination of characters: endotergum weakly modified; posterior margin usually flat (rarely modified); outer area without setae; marginal bristles arranged in 1–5 rows; middle area often with a single row of circular cuticular impressions; tarsi of leg pairs 5–21 usually with more than one apical spine (rarely with a single apical spine) and several ventral spines; male anterior telopods with four podomeres distal to syncoxite; telopoditomere 2 bearing a large, curved process forming a clamp-like structure; telopoditomeres 3 and 4 simple; male posterior telopods with four telopoditomeres; process of telopoditomere 2 slender and apically curved; telopoditomeres 3 and 4 with a membranous ledge; telopoditomere 3 with a row of large, crenulated, sclerotised teeth; telopoditomere 4 with one or two sclerotised spines; female vulvae conspicuous and slender, operculum usually rounded.

See more recent diagnoses for *Zephronia* sensu lato and *Zephronia* sensu stricto in Srisonchai et al. (2024).

### *Zephronia* sensu stricto

*Zephronia* s.s. currently includes *Z. chantaburiensis*, *Z. chrysomallos*, *Z. dawydoffi*, *Z. enghoffi*, *Z. erawani*, *Z. golovatchi*, *Z. hui* Liu & Wesener, 2022, *Z. konkakinhensis* Semenyuk, Golovatch & Wesener, 2018, *Z. lannaensis*, *Z. laotica*, *Z. macula*, *Z. medogensis* Zhao & Liu, 2022, *Z. montis* Semenyuk, Golovatch & Wesener, 2018, *Z. ovalis*, *Z. panhai*, *Z. phrain*, *Z. siamensis*, *Z. sukhothaiensis*, *Z. tamarindina* sp. nov., *Z. tratensis*, *Z. triforma* sp. nov., *Z. zhouae* Zhao & Liu, 2022.

### List of 18 *Zephronia* species in Thailand

1. *Z. chantaburiensis* Srisonchai & Wesener, 2024
2. *Z. chrysomallos* Bhansali & Wesener, 2022
3. *Z. dawydoffi* Attems, 1953
4. *Z. enghoffi* Srisonchai & Likhitrakarn, 2021
5. *Z. erawani* Bhansali & Wesener, 2022
6. *Z. golovatchi* Srisonchai & Likhitrakarn, 2021
7. *Z. lannaensis* Likhitrakarn & Golovatch, 2021
8. *Z. laotica* Wesener, 2019
9. *Z. macula* Srisonchai & Wesener, 2024
10. *Z. ovalis* Gray, 1832
11. *Z. panhai* Srisonchai & Likhitrakarn, 2021
12. *Z. phrain* Likhitrakarn & Golovatch, 2021
13. *Z. siamensis* Hirst, 1907
14. *Z. sukhothaiensis* Srimongkol & Srisonchai, 2026
15. *Z. tamarindina* sp. nov.
16. *Z. tratensis* Srimongkol & Srisonchai, 2026
17. *Z. triforma* sp. nov.
18. *Z. viridisoma* Rosenmejer & Wesener, 2021

Among the examined material, colour variation initially suggested the presence of multiple distinct taxa. In particular, one species, *Z. triforma* sp. nov., exhibits three distinct colour morphs, which could be misinterpreted as representing separate species based on external appearance alone. In contrast, *Z. tamarindina* sp. nov. and the two previously described species each show a single, consistent colour form. However, integrative analyses combining morphological characters and *COI* sequence data confirm that the three colour morphs belong to a single species, *Z. triforma* sp. nov., whereas the remaining populations correspond to two new species and two previously described species.

In this study, we focus on describing the two new species and the two newly recorded species from Thailand.

#### Zephronia dawydoffi

Taxon classification

Animalia

SphaerotheriidaZephroniidae

Attems, 1953

58B0FAD1-D1BE-5E98-A465-E747FFC89F83

Figs 1, 2, 3, 4, 5, 6, 22A, B, 23A–E, 24

Zephronia dawydoffi Attems, 1953: 156.
Zephronia dawydoffi — Jeekel 2001: 18; Likhitrakarn et al. 2015: 177; Wesener 2016: 31; Wesener 2019: 205; Likhitrakarn et al. 2021: 23; Rosenmejer et al. 2021: 111; Srisonchai et al. 2021: 26; Bhansali and Wesener 2022: 359; Donworth and Wesener 2024: 547; Srisonchai et al. 2024: 30; Srimongkol et al. 2026: 31.

##### Material examined.

• 18 ♂♂ 33 ♀♀ (MZKKU); Thailand, Khon Kaen Province, Mueang District, Thung Setthi Temple; 16°24'33"N, 102°53'05.7"E; ca. 150 m a.s.l.; 12 July 2022; leg. C. Wilaisorn and MZKKU students; • 5 ♂♂ 7 ♀♀ (MZKKU); Thailand, Buri Ram Province, Khu Mueang District, Dong Kheng rubber plantation; 15°19'46"N, 103°00'59"E; ca. 159 m a.s.l.; 6 July 2022; leg. C. Wilaisorn and MZKKU students; • 4 ♂♂ 2 ♀♀ (MZKKU); Thailand, Buri Ram Province, Mueang District, Khao Kradong; 14°56'21.5"N, 103°05'28.8"E; ca. 213 m a.s.l.; 10 September 2023; leg. C. Wilaisorn and MZKKU students; • 3 ♀♀ (MZKKU); Thailand, Buri Ram Province, Mueang District, Ban Khok Ta Sing Waterfall; 14°55'22.8"N, 103°06'57.9"E; ca. 180 m a.s.l.; 5 June 2022; leg. C. Wilaisorn and MZKKU students. • 12 ♂♂ 21 ♀♀ (MZKKU); Thailand, Buri Ram Province, Khu Mueang District, Non Somboon Monastery; 15°16'32"N, 102°59'13"E; ca. 200 m a.s.l.; 31 July 2022; leg. C. Wilaisorn and MZKKU students.

**Figure 2.**
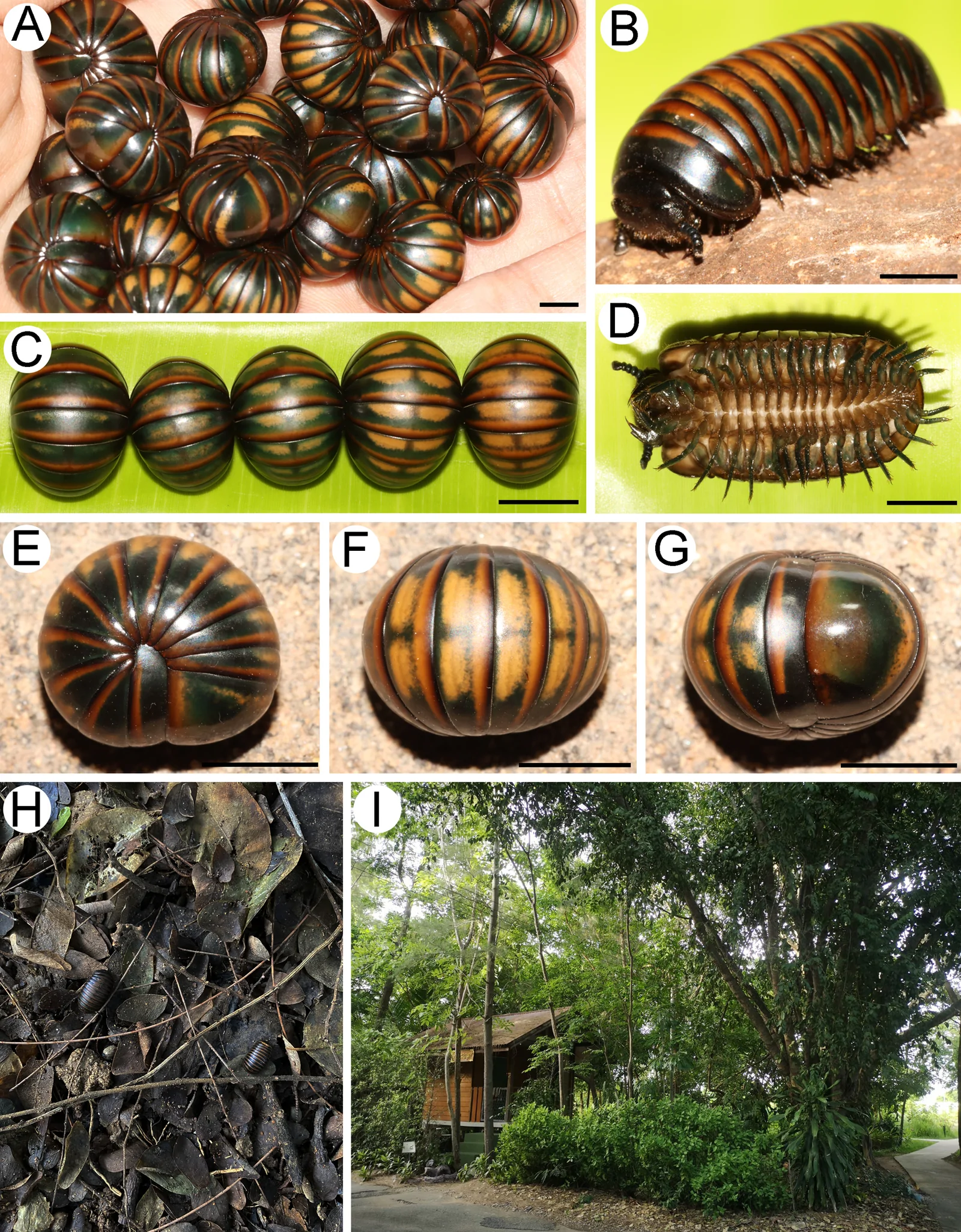
Photograph of live specimens of *Zephronia dawydoffi* Attems, 1953 and habitats. **A–G**. Live specimens of a population from Khon Kaen Province, Thailand; **H**. Pile of leaf litter; **I**. Agricultural area. Scale bars: 5.0 mm.

**Figure 3.**
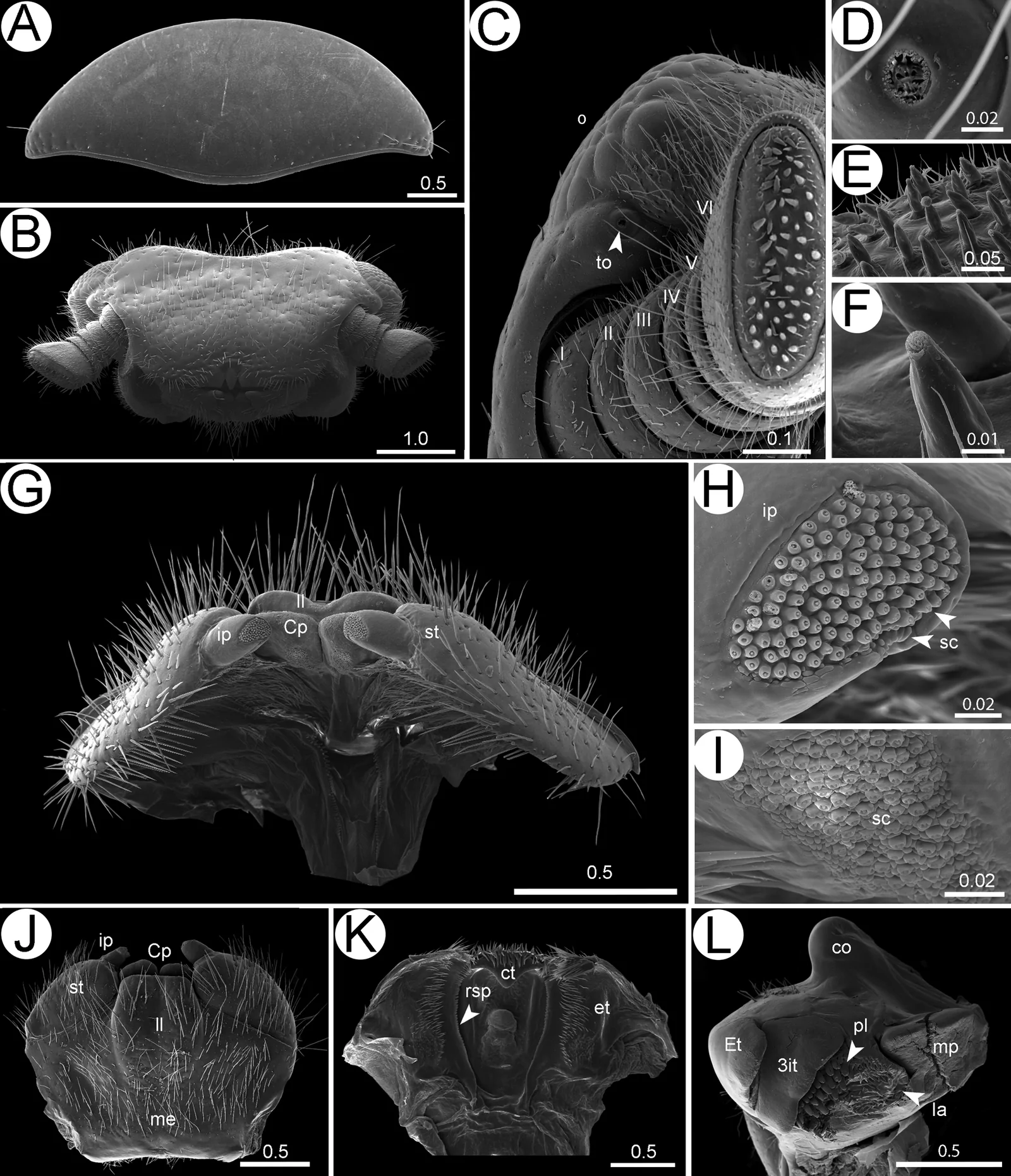
*Zephronia dawydoffi* Attems, 1953, ♂ from Thung Setthi Temple, Khon Kaen Province, Thailand (MZKKU) — SEM. **A**. Collum, dorsal view; **B**. Head, anterior view; **C**. Antenna, lateral view; **D**. Tömösváry’s organ; **E, F**. Apical cones, lateral view; **G, J**. Gnathochilarium, anterior and ventral views, respectively; **H**. Sensory cones on inner palpi, ventral view; **I**. Sensory cones on central pad, ventral view; **K**. Epipharynx, ventral view; **L**. Right mandible, mesal view. Abbreviations: 3it = 3-combed inner tooth, co = condylus, Cp = central pad, ct = central tooth, et = external tooth of epipharynx, Et = external tooth of mandible, Ia = inner area, il = incisura lateralis, ip = inner palpi, ll = lamellae linguales, me = mentum, mp = molar plate, o = ommatidia, pl = pectinate lamellae, rsp = row of spines, sc = sensory cone, st = stipites, t = Tömösváry’s organ. Scale bars in millimetres.

**Figure 4.**
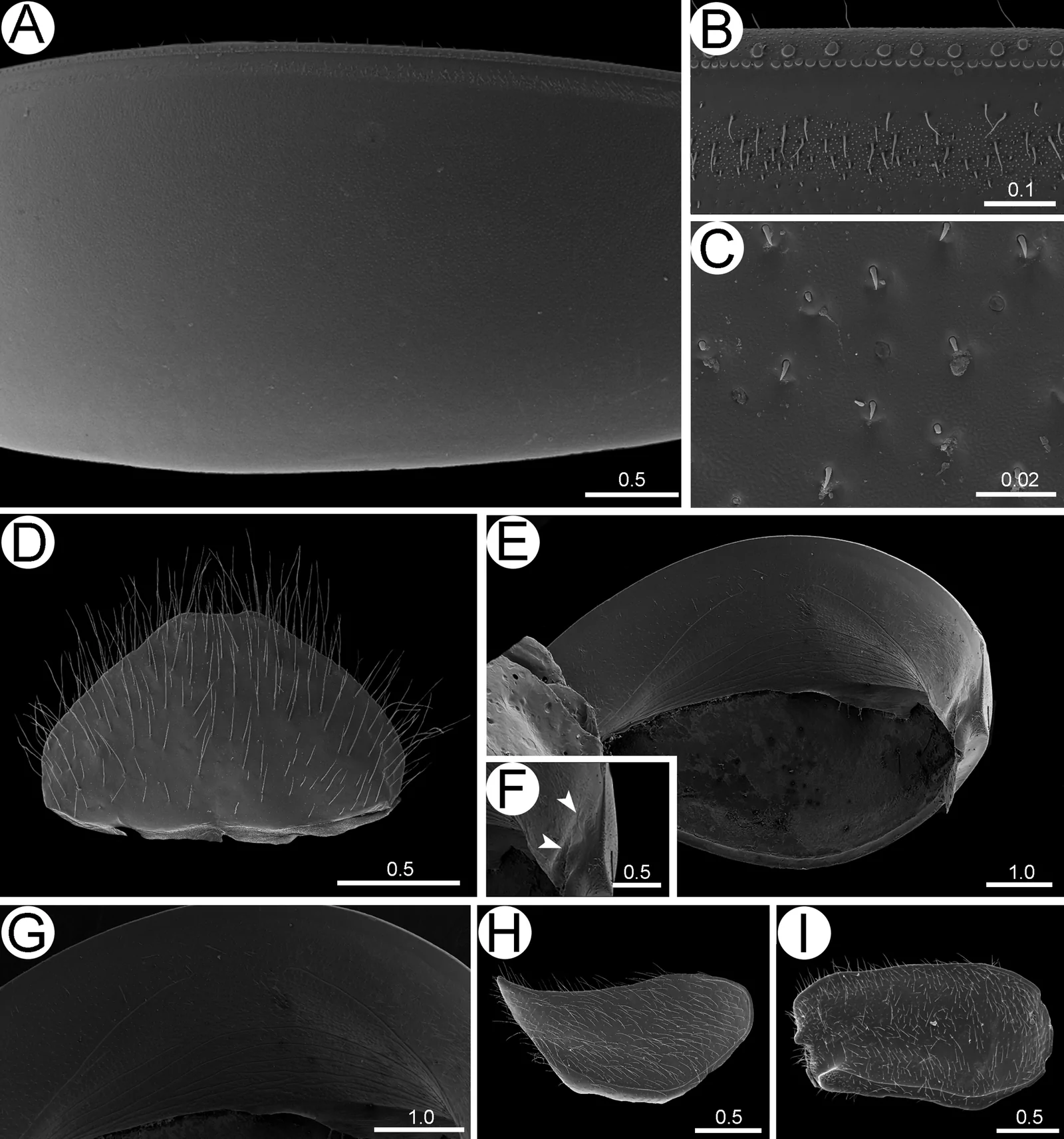
*Zephronia dawydoffi* Attems, 1953, ♂ and ♀ from Thung Setthi Temple, Khon Kaen Province, Thailand (MZKKU) — SEM. **A–C**. Surface of midbody tergite, all in dorsal views (overview, anterior margin, surface, respectively); **D**. Subanal plate; **E**. Anal shield, underside, ventral view; **F**. Locking carinae; **G**. Surface of anal shield; **H**. First pleurite; **I**. Midbody pleurite. Scale bars in millimetres.

**Figure 5.**
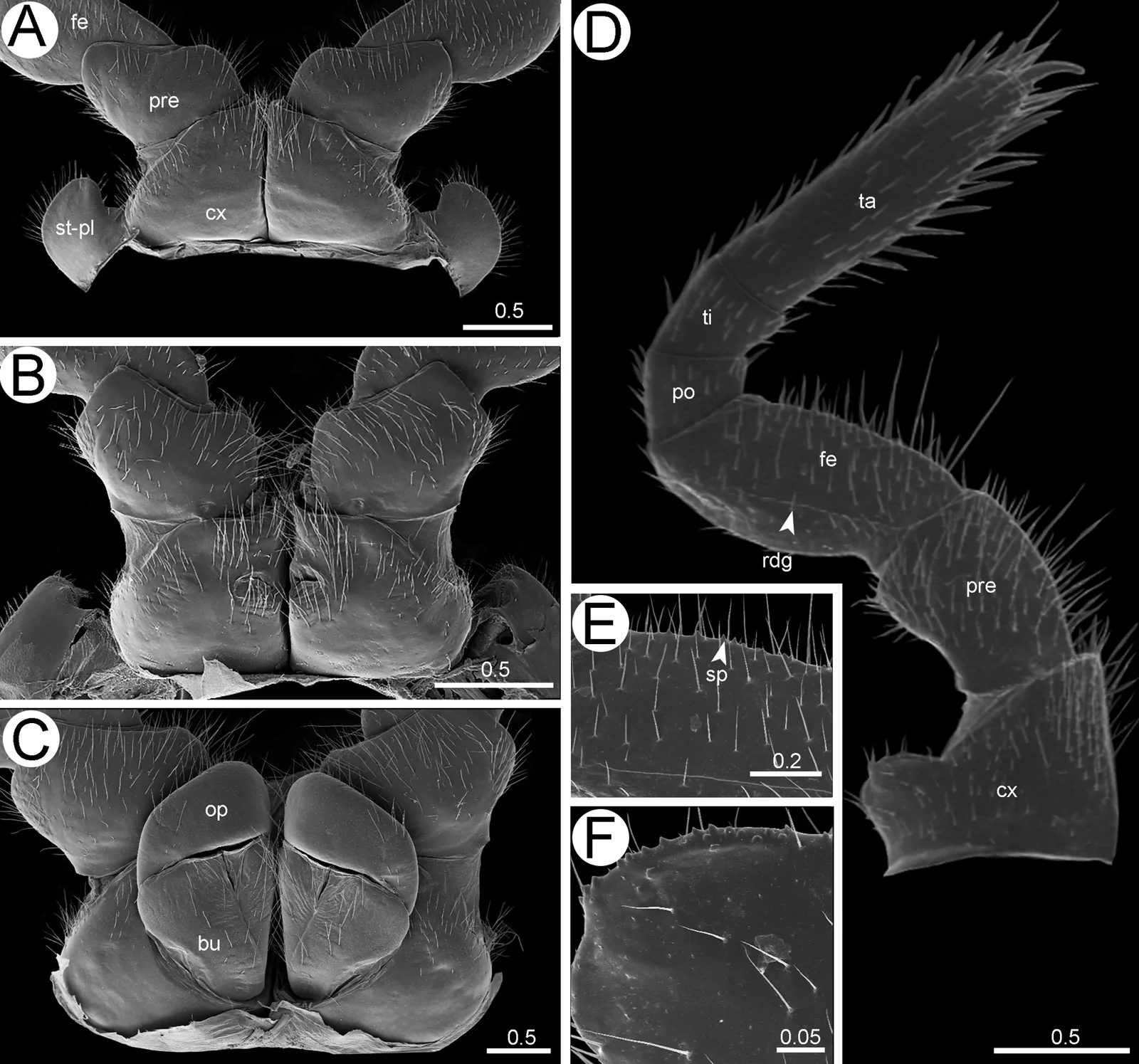
*Zephronia dawydoffi* Attems, 1953, ♂ and ♀ from Thung Setthi Temple, Khon Kaen Province, Thailand (MZKKU) — SEM. **A**. Legs 1; **B**. Legs 2 with gonopores; **C**. Leg 2 with vulvae; **D**. Midbody legs (arrow points to femoral ridge); **E**. Small triangular spines on femur; **F**. Coxal process on midbody legs. Abbreviation: bu = bursa, cx = coxa, fe = femur, op = operculum, po = postfemur, pre = prefemur, rdg = femoral ridge, st-pl = stigmatic plate, ta = tarsus, ti = tibia. Scale bars in millimetres.

**Figure 6.**
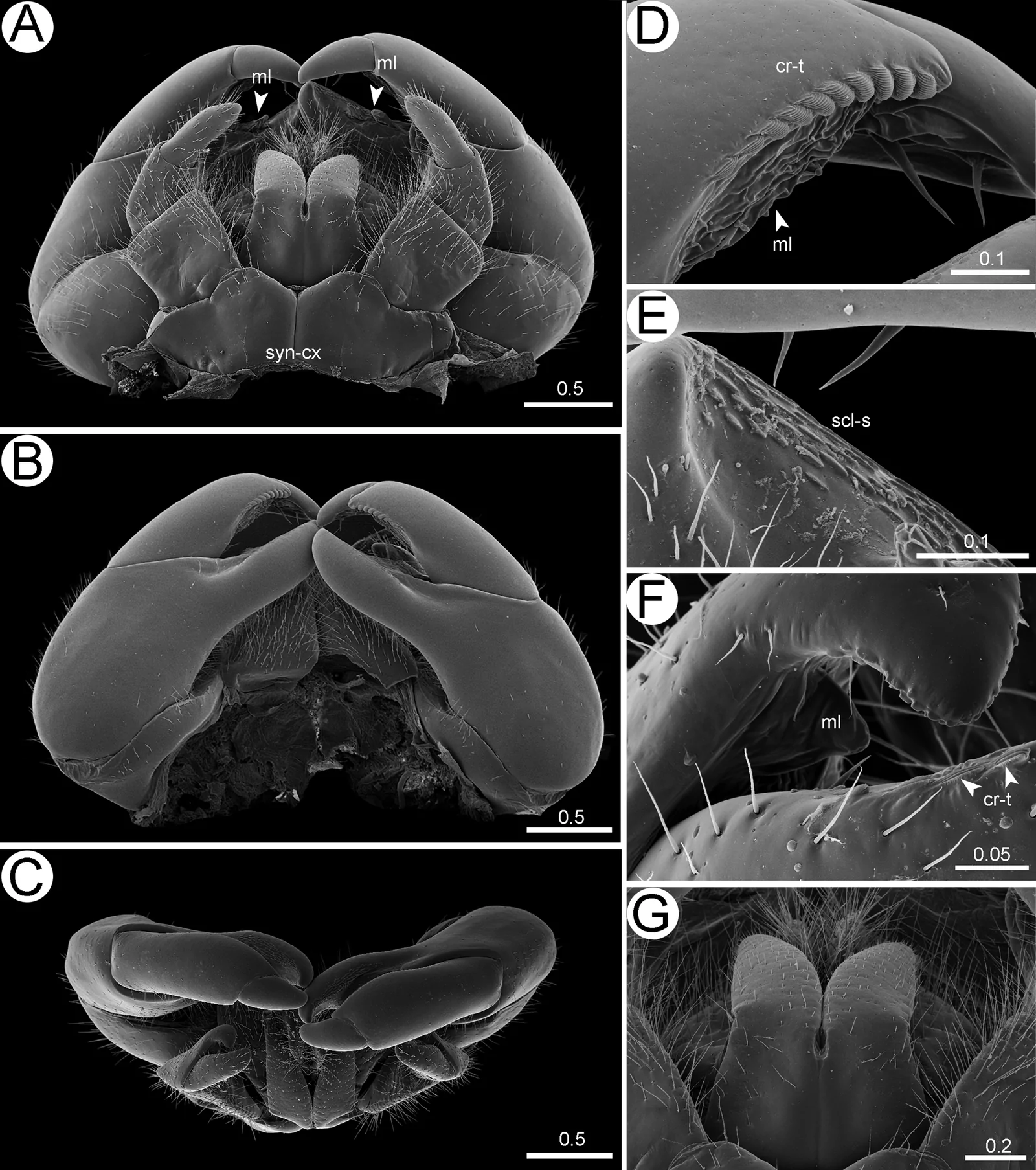
*Zephronia dawydoffi* Attems, 1953, ♂from Thung Setthi Temple, Khon Kaen Province, Thailand (MZKKU) — SEM. **A–C**. Overview of telopods (anterior, posterior, and ventral views, respectively); **D**. Crenulated teeth of posterior telopods, posterior view; **E**. Sclerotised spots on immovable finger of posterior telopod, sublateral view; **F**. Anterior telopod, ventral view; **G**. Inner horns, anterior view. Abbreviations: cr-t = crenulated teeth, ml = membranous lobe, scl-s = sclerotised spots, syn-cx = syncoxite. Scale bars in millimetres.

##### Re-diagnosis based on Thai specimens.

In *Z. dawydoffi*, the Tömösváry’s organ is positioned at the brim rather than inside the antennal groove (Fig. 3B, C), confirming its placement within *Zephronia* s.s. species group (see Semenyuk et al. 2018), which is also supported by genetic evidence (Fig. 1). *Z. dawydoffi* differs from all other *Zephronia* s.s. species, except for *Z. laotica*, *Z. ovalis*, and *Z. siamensis*, in the presence of colour patches on the tergites as well as in the posterior margin of the endotergum which is modified (Fig. 22A, B), and the lack of an apical spine in the third leg-pair. It differs from *Z. laotica*, *Z. ovalis*, and *Z. siamensis* in the shape of the anterior telopods with the process of telopoditomere 2 broadened, shorter than telopoditomere 3+4 (Figs 6C, 6F, 23A–C), the process of telopoditomere 2 of the posterior telopods long (Figs 6B, 23D, 23E), and the presence of two locking carinae on the underside of anal shield (Fig. 4F; except for *Z. laotica*). The genetic differentiation of the 658 bp *COI* fragment between *Z. dawydoffi* and other *Zephronia* ranged from 8.68 to 21.92%.

##### Type locality.

CAMBODIA (Ream, Sre Umbell; Kampot, Kampong Speu).

##### Description of specimens from Thailand.

***Measurements***. Males: body length = 10.5–18.8 mm; width (broadest), of thoracic shield = 5.3–8.6 mm, of tergite 7 = 5.6–9.3 mm; highest of thoracic shield = 3.1–5.5 mm, of tergite 7 = 3.3–6.9 mm. Females: body length = 11.5–22.0 mm; width (broadest), of thoracic shield = 5.8–10.6 mm, of tergite 7 = 6.4–11.7 mm; highest of thoracic shield = 4.0–7.1 mm, of tergite 7 = 4.4–8.2 mm.

***Colouration*** (Fig. 2A–G). Live specimens dark green with yellow semicircular colour markings. Head and collum dark green. Thoracic shield and tergites dark green (rarely greenish-brown or dark brown), each with yellowish brown posterior bands and two yellow (rarely pale yellow) dorsal patches per tergite. Anal shield dark green with a conspicuous yellow patch anteriorly, and orangish brown on posterior margin. Antenna and legs green. Venter pale brown. Preserved specimens in 95% ethanol after 1.5 years changed to brownish green, some faded to yellowish green.

***Head*** (Fig. 3B–D). Trapeziform, broad, stout; densely setose over entire surface including mouthparts. With 77–93 ommatidia (ocelli). Aberrant ommatidium situated at brim of antennal groove. Tömösváry’s organ separated from eye field and located between base of antenna and ommatidia. No sclerotised ridge between antennal groove and ommatidia.

***Antennae*** (Fig. 3B, C, E, F). Short and stout; emerging from sides of head, covered by noticeable long setae; last antennomere reaches back posteriorly to leg pair 2. Lengths of antennomeres in descending order: 6 > 5 > 4 = 3 > 2 > 1. Antennomere 6 flattened, axe-shaped; with sensilla basiconica around apical disc. Apical disc flat; with 59–73 apical cones in males, and 44–62 apical cones in females.

***Epipharynx*** (Fig. 3K). Apically with a triangular projected central tooth (ct); with conspicuous inner tooth; inner area with a row of triangular fringed spines (rsp); outer area with several slender external teeth (et).

***Gnathochilarium*** (Fig. 3G–J). Lamellae linguales (ll) rectangular, apically concave; covered by long setae. Central pads (Cp) modified; with two types of sensory cones (sc), first type small without tubercle apically, second type larger with a concave pad apically containing a single internal tubercle. Stipites (st) robust, with dense and long setae, located laterally to lamellae linguales. Mentum (me) broad and fused, densely setose. Lateral palpi inconspicuous. Inner palpi (ip) with numerous cylindrical sensory cones (sc) located in single field, each cone concave apically and containing a single internal tubercle.

***Mandibles* (*gnathal lobe*)** (Fig. 3L). With single undivided external tooth (Et) and 3-combed inner tooth (3it). With 4–5 rows of pectinate lamellae (pl). Inner area (Ia) with several rows of small slender teeth. Molar plate (mp) relatively flat. Condylus (co) conspicuous, apically with a single ridge.

***Tegument*** (Figs 2A–G, 4). Glabrous; collum, thoracic shield, tergites and anal shield covered by tiny setae; each seta emerged from a pit. Tergites and anal shield sparsely setose anteriorly and without setae posteriorly.

***Collum*** (Fig. 3A). Subsemicircular; lateral edges triangular, blunt; with a few long setae.

***Thoracic shield***. Anterior margin with shallow canal covered with a few scattered long setae, with 5–7 small keels.

***Midbody tergite*** (Fig. 4A–C). Dorso-anterior margin with a row of circular impressions and small tubercles. Inner area with a shallow groove, separated from anterior margin by a row of crenate barriers. Tips of paratergites projected posteroventrally.

***Anal shield*** (Fig. 4E–G). Sexually dimorphic, females well-rounded, males rectangular and slender. Anteriorly with a row of circular impressions and small tubercles. Inner area with a shallow groove, separated from margin by a row of crenate barriers. Underside with two dark locking carinae; length of each locking carina as long as those of tergites; densely setose especially on lateral margin.

***Endotergum of thoracic shield and midbody tergite*** (Fig. 22A, B). Thoracic shield similar to midbody tergite. Posterior margin (pm) distinctly modified, with rectangular projections, length of each projection being longer than height. Outer area (oa) with several small denticles. Marginal bristles arranged in two rows; tips of the longest bristles not reaching to posterior margin. Middle area (ma) with a single row of oval cuticular impressions (cp); distance between each impression almost twice as long as individual diameter. Cuticular impressions of thoracic shield shallower than those of midbody tergite. Inner area (ia) with long setae.

***Pleurite* (*laterotergite*)** (Fig. 4H, I). Densely setose. First pleurite boomeranglike, with pointed edge, reaching towards paratergite. Pleurite 2 slightly widened. The remaining pleurites flat, rectangular.

***Subanal plate of female*** (Fig. 4D). Densely setose; wide; triangular; apical margin weakly concave, narrow.

***Stigmatic plates*** (Fig. 5A). First stigmatic plate quite slender, flat antero-posteriorly; semicircular; apex round. Second stigmatic plate in both sexes triangular.

***Legs*** (Fig. 5). All podomeres densely setose. Coxa (cx) large, as long as length of prefemur (pre); first and second leg-pairs without coxal process; leg-pairs 3–21 with large and serrated processes. Prefemur wide, stout; apico-mesally without projection. Femur (fe) slightly long, 1.5 × longer than prefemur; with conspicuous long ridge (rdg); apico-mesally with inconspicuous projection; mesal margin dentate, with seven or eight small triangular microscopic spines (sp). Postfemur (po) and tibia (ti) short. Tarsus (ta) of midbody legs long and slender, shorter than femur+postfemur; first three leg-pairs without an apical spine; leg-pair 1 with 1–3 ventral spines; leg-pair 2 with two or three ventral spines; leg-pair 3 with 3–5 ventral spines; leg pair 4–21 with 6–9 ventral spines and two or three apical spines. In leg 10, femur 1.5 ×, tarsus 3 × longer than wide. Claw normal, with small notch at base.

Male sexual characters (Fig. 5B). Gonopore surrounded by long setae; covered with a divided, sclerotised plate. Apical part of coxa 2 without membranous process.

***Anterior telopods*** (Figs 6A, 6C, 6F, 23A–C). Consisting of four telopoditomeres. First telopoditomere conspicuous, rectangular, slightly longer than wide. Telopoditomere 2 large. Process of telopoditomere 2 (= immovable finger) broad; shorter than combination of telopoditomeres 3 and 4 (= movable finger); clearly seen in posterior view; strongly curved toward midway of movable finger; inner margin with a single membranous lobe and numerous sclerotised spots (scl-s). Telopoditomere 3 long and slender; twice as long as telopoditomere 4; partly demarcated from telopoditomere 4 by shallow suture (rarely fused with telopoditomere 4); inner margin with single spine; apically with five or six small crenulated teeth (cr-t) and two conspicuous spines. Telopoditomere 4 tip obtuse. All telopoditomeres densely setose, except for basal part of telopoditomere 1 and apical part of immovable finger.

***Posterior telopods*** (Figs 6A–E, 6G, 23D, 23E). Consisting of four telopoditomeres. First telopoditomere swollen, broad; as wide as long. Telopoditomere 2 large and slender. Process of telopoditomere 2 (= immovable finger) quite long, 2.5 × longer than wide; shorter than a combination of telopoditomeres 3 and 4 (= movable finger); apically obtuse, slightly curved; inner margin with two membranous lobes (ml) and numerous conspicuous sclerotised spots (scl-s). Telopoditomere 3 long and slender; 2.5 × longer than wide; with a large membranous ledge and single spine at inner margin; posteriorly with a row of nine or ten crenulated teeth (cr-t). Telopoditomere 4 short; 2.5 × shorter than telopoditomere 3; 1.5 × longer than wide; tip tapering; inner margin with two long spines located on a large membranous ledge. Telopoditomeres 1 and 2 covered by long setae anteriorly, sparse setae posteriorly. Telopoditomere 3 sparsely setose at inner margin. Telopoditomere 4 glabrous. Inner horns densely setose, swollen at base; tips pointed, directed posteriad.

***Female sexual characters*** (Fig. 5C). Vulva conspicuous, large, with a few long setae; covering more than half of coxa; located at mesal margin; extending mesally to base of prefemur. Operculum (op) round and slender, rectangular; tip round. Bursa (bu) large, fused into triangular shape, clearly separated from operculum by triangular groove. Apical part of coxa 2 without coxal process.

##### Remarks.

Variation in body colouration and pattern was observed among specimens. Most individuals displayed dark green tergites, while others exhibited greenish-brown or dark brown tones. Typically, two yellow patches were present on dorsal tergites, though in some specimens these patches appeared faded or paler in colour. The patch shapes also varied, most commonly forming two semicircular markings (Fig. 2A, C, F). In addition, telopoditomeres 3 and 4 of anterior telopods are only partly demarcated in most individuals (Fig. 23A–C), but in some specimens appear completely fused (Fig. 6A, C).

Morphological differences were observed between Thai specimens and the original description by Attems (1953), as well as in comparison with the syntypes deposited in the NHMW. The body size of the type specimens was between 11.0–13.0 mm in length and 5.8–6.0 mm in width, whereas the Thai specimens were larger, with a range of 10.5–22.0 mm in length and 5.6–11.7 mm in width. The thoracic shield of the Thai individuals bears 5–7 weak keels, which are absent from the type description. Furthermore, the posterior margin of the endotergum is described as flat in the type material, but appears modified in the Thai specimens. In the original description, telopoditomeres 3 and 4 of the anterior telopods are fused together, while those of Thai specimens were usually separated by a shallow suture, with only a very few specimens showing a complete fusion. Similarly, telopoditomere 2 of posterior telopods was described as having a single membranous lobe, while the Thai material consistently exhibits two lobes (like most species in the genus). These differences are interpreted as intraspecific variation within the species. In addition, some apparent discrepancies between the type specimens and the Thai material likely result from differences in observational resolution and the use of modern imaging techniques, which allow finer morphological details to be detected.

##### Distribution and habitat.

*Zephronia dawydoffi* in Thailand is currently known from two provinces in the northeastern region: four localities in the Buri Ram Province and only one locality in the Khon Kaen Province (Fig. 24). This species inhabits agricultural areas, particularly rubber plantations characterised by tall trees, limited sunlight, and fine soil. Specimens were primarily collected during the day, typically found sheltering beneath leaf litter piles (Fig. 2H, I).

Compared with its type locality in southern Cambodia, the known distribution of *Z. dawydoffi* is extended into Thailand, representing a geographic range spanning approximately 300–700 km.

#### Zephronia laotica

Taxon classification

Animalia

SphaerotheriidaZephroniidae

Wesener, 2019

636ABD8D-CAAE-5F83-B5F9-C4954AEE00E6

Figs 1, 7–11, 22C, D, 23F–J, 24

Zephronia laotica Wesener, 2019: 205.
Zephronia laotica — Srisonchai et al. 2021: 24; Likhitrakarn et al. 2021: 23; Rosenmejer et al. 2021: 111; Bhansali and Wesener 2022: 359; Srisonchai et al. 2023: 44; Donworth and Wesener 2024: 547; Srisonchai et al. 2024: 30; Sapparojpattana et al. 2025: 53; Sapparojpattana et al. 2026: 56; Srimongkol et al. 2026: 31.

##### Material examined.

• 10 ♂♂ 7 ♀♀ (MZKKU); Thailand, Si Sa Ket Province, Khun Han District, Mae Lamoon Durian plantation; 14°33'00"N, 104°31'51"E; ca. 228 m a.s.l.; 29 July 2022; leg. C. Wilaisorn and MZKKU students; • 10 ♂♂ 12 ♀♀ (MZKKU); Thailand, Si Sa Ket Province, Khun Han District, Kantrom Tai Temple; 14°34'32"N, 104°20'33"E; ca. 188 m a.s.l.; 6 October 2024; leg. C. Wilaisorn and MZKKU students; • 2 ♀♀ (MZKKU); Thailand, Si Sa Ket Province, Khun Han District, Ta Nuan Rubber plantation; 14°33'47.9"N, 104°21'24.2"E; ca. 180 m a.s.l.; 10 June 2023; leg. C. Wilaisorn and MZKKU students.

##### Re-diagnosis based on Thai material.

The position of the Tömösváry’s organ is located at the brim rather than inside the antennal groove (Fig. 8B, C), confirming the placement of *Z. laotica* as a member of *Zephronia* s.s. species group. The presence of colour patches on tergites, as well as a modified posterior margin of the endotergum, and the lack of an apical spine in the third leg-pair are three characters only shared with *Z. dawydoffi*, *Z. ovalis*, and *Z. siamensis*. *Z. laotica* differs from them in the combination of the following characters: process of telopoditomere 2 of anterior telopods wide (Figs 11F, 23F, 23H); process of telopoditomere 2 of posterior telopods long and swollen (Figs 11B, 23I, 23J); two locking carinae on the underside of the anal shield (Fig. 9F) (except for *Z. dawydoffi*). The genetic differentiation between *Z. laotica* and other *Zephronia* species ranged from 12.79 to 22.98% of *COI* sequences.

**Figure 7.**
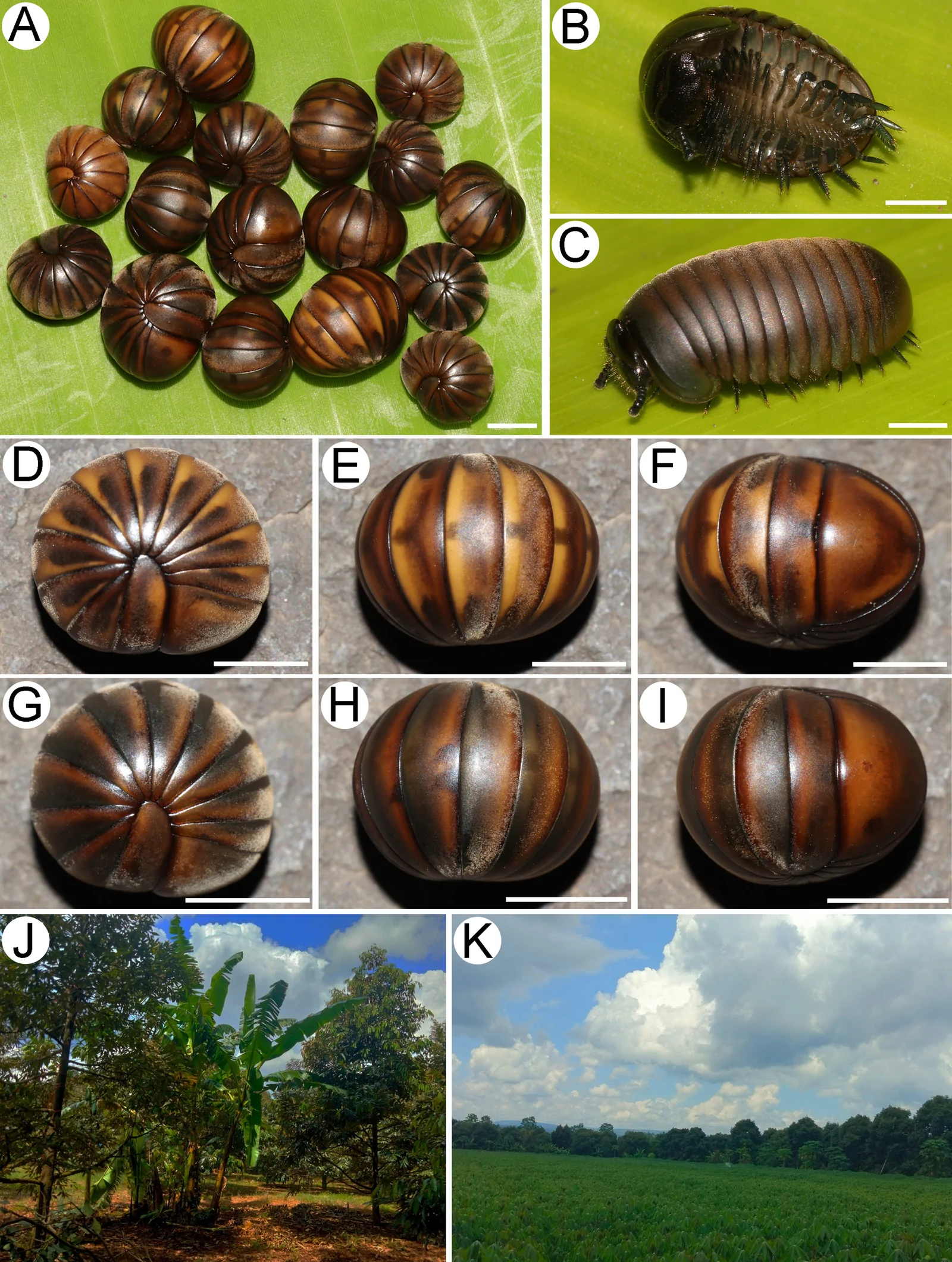
Photograph of live specimens of *Zephronia laotica* Wesener, 2019 and habitats. **A–I**. Live specimens of a population from Si Sa Ket Province, Thailand; **J, K**. Agricultural areas. Scale bars: 5.0 mm.

**Figure 8.**
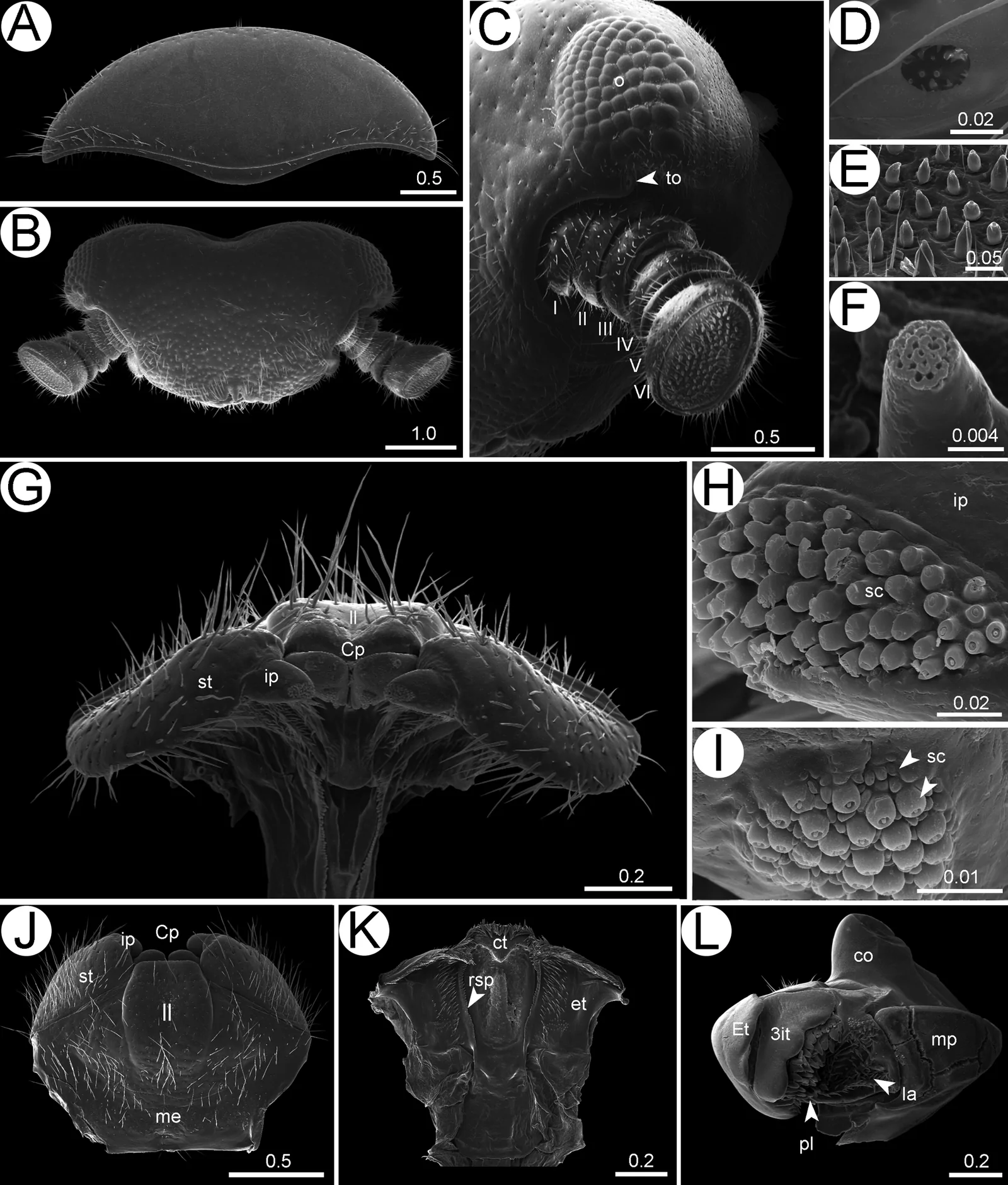
*Zephronia laotica* Wesener, 2019, ♂ from Mae Lamoon Durian plantation, Si Sa Ket Province, Thailand (MZKKU) — SEM. **A**. Collum, dorsal view; **B**. Head, anterior view; **C**. Antenna, lateral view; **D**. Tömösváry’s organ; **E, F**. Apical cones, lateral view; **G, J**. Gnathochilarium, anterior and ventral views, respectively; **H**. Sensory cones on inner palpi, ventral view; **I**. Sensory cones on central pad, ventral view; **K**. Epipharynx, ventral view; **L**. Right mandible, mesal view. Abbreviations: 3it = 3-combed inner tooth, co = condylus, Cp = central pad, ct = central tooth, et = external tooth of epipharynx, Et = external tooth of mandible, Ia = inner area, il = incisura lateralis, ip = inner palpi, ll = lamellae linguales, me = mentum, mp = molar plate, o = ommatidia, pl = pectinate lamellae, rsp = row of spines, sc = sensory cone, st = stipites, t = Tömösváry’s organ. Scale bars in millimetres.

**Figure 9.**
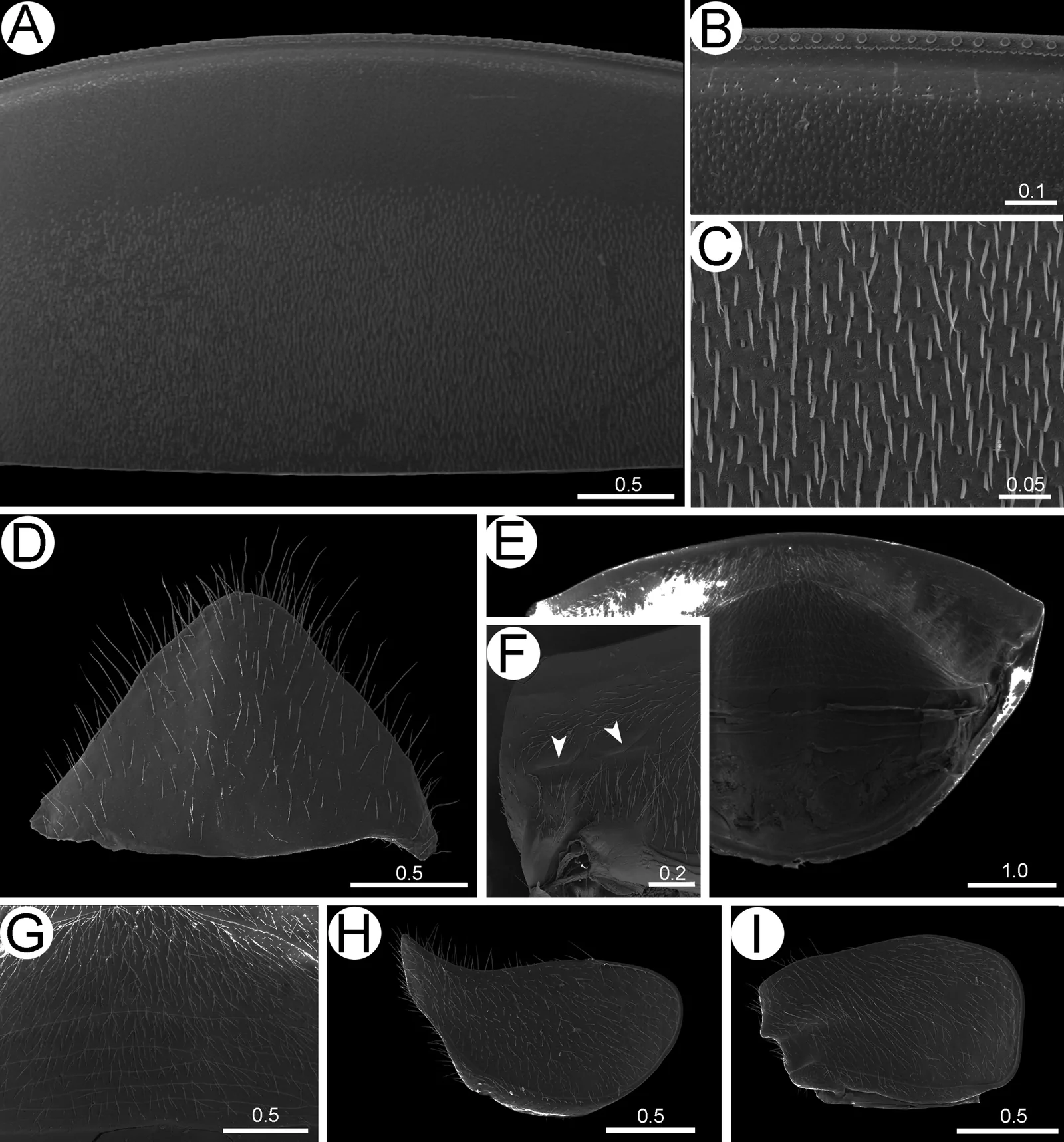
*Zephronia laotica* Wesener, 2019, ♂ and ♀ from Mae Lamoon Durian plantation, Si Sa Ket Province, Thailand (MZKKU) — SEM. **A–C**. Surface of midbody tergite, all in dorsal views (overview, anterior margin, surface, respectively); **D**. Subanal plate; **E**. Anal shield, underside, ventral view; **F**. Locking carinae (arrowheads point to carinae); **G**. Surface of anal shield; **H**. First pleurite; **I**. Midbody pleurite. Scale bars in millimetres.

**Figure 10.**
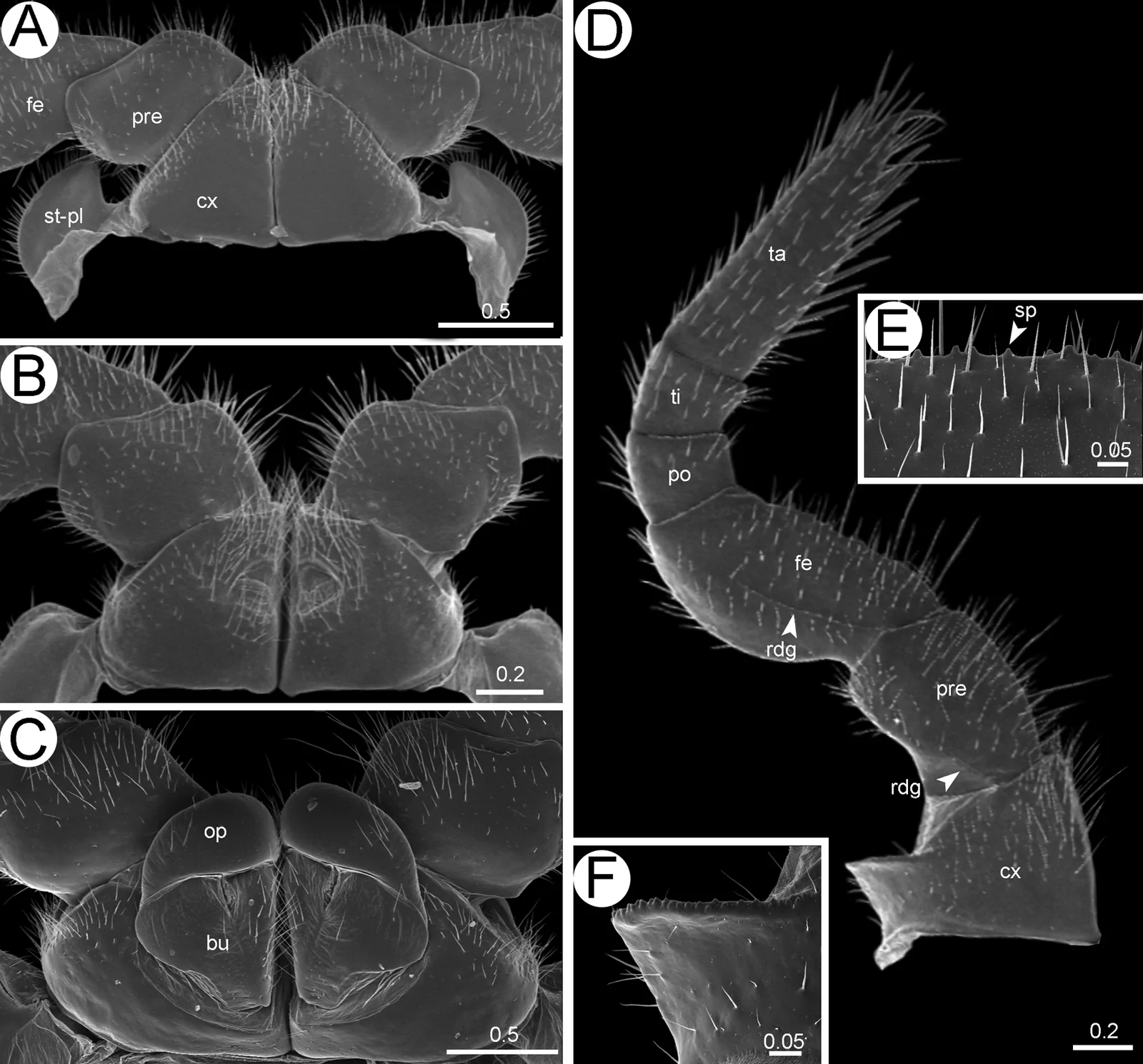
*Zephronia laotica* Wesener, 2019, ♂ and ♀ from Mae Lamoon Durian plantation, Si Sa Ket Province, Thailand (MZKKU) — SEM. **A**. Legs 1; **B**. Legs 2 with gonopores; **C**. Leg 2 with vulvae; **D**. Midbody legs (arrow points to femoral ridge); **E**. Small triangular spines on femur; **F**. Coxal process on midbody legs. Abbreviation: bu = bursa, cx = coxa, fe = femur, op = operculum, po = postfemur, pre = prefemur, rdg = femoral ridge, st-pl = stigmatic plate, ta = tarsus, ti = tibia. Scale bars in millimetres.

**Figure 11.**
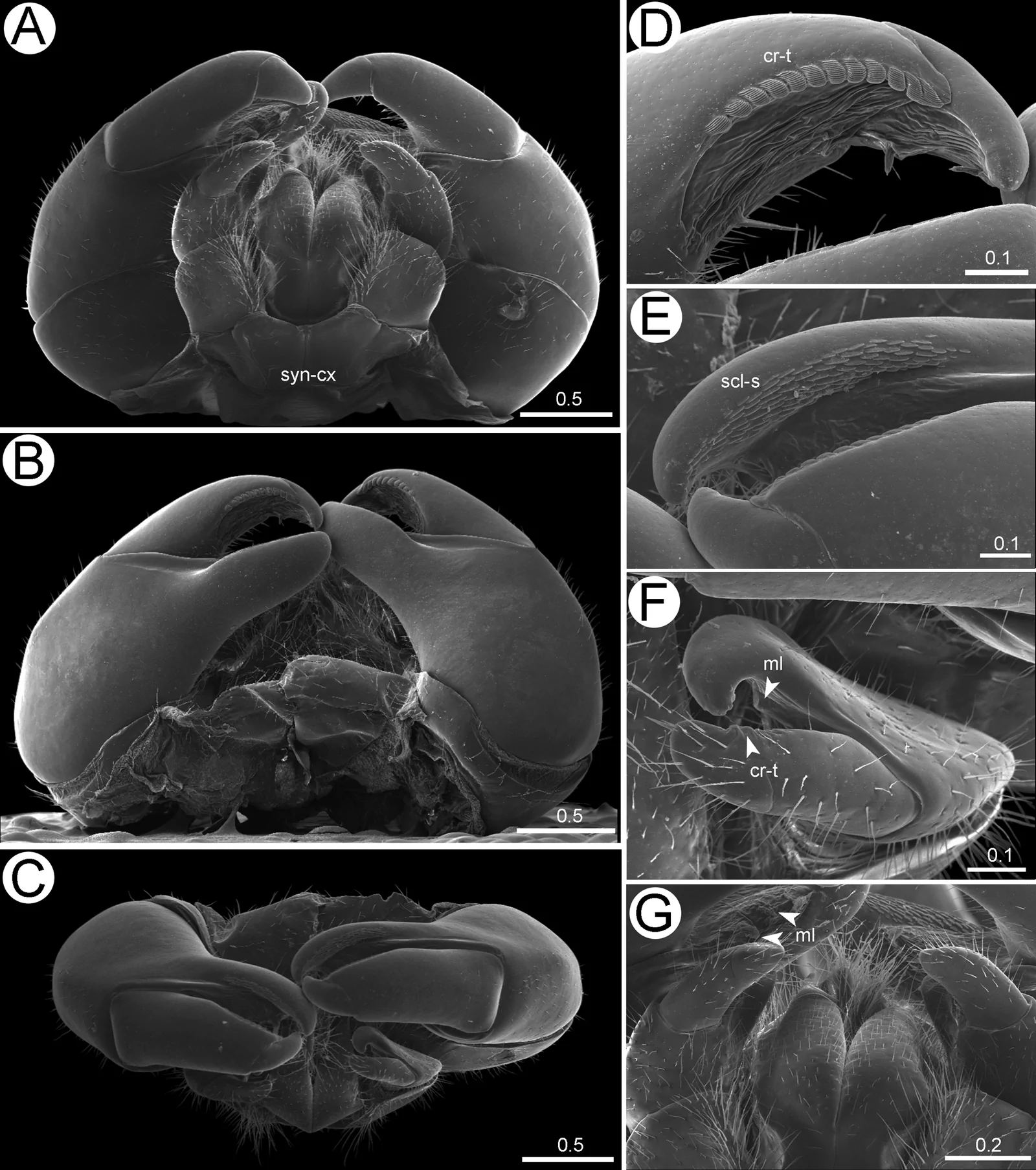
*Zephronia laotica* Wesener, 2019, ♂ from Mae Lamoon Durian plantation, Si Sa Ket Province, Thailand (MZKKU) — SEM. **A–C**. Overview of telopods (anterior, posterior, and ventral views, respectively); **D**. Crenulated teeth of posterior telopods, posterior view; **E**. Sclerotised spots on immovable finger of posterior telopod, sublateral view; **F**. Anterior telopod, ventral view; **G**. Inner horns, anterior view. Abbreviations: cr-t = crenulated teeth, ml = membranous lobe, scl-s = sclerotised spots. Scale bars in millimetres.

##### Type locality.

LAOS, Champasak Province, east of Mekong, Garden of Erawan Riverside Hotel.

##### Description of specimens from Thailand.

***Measurements***. Males: body length = 10.2–15.2 mm; width (broadest), of thoracic shield = 5.2–7.9 mm, of tergite 7 = 5.5–8.1 mm; highest of thoracic shield = 4.4–6.6 mm, of tergite 7 = 3.8–6.1 mm. Females: body length = 11.4–15.5 mm; width (broadest), of thoracic shield = 5.8–10.6 mm, of tergite 7 = 6.2–11.1 mm; highest of thoracic shield = 4.0–7.8 mm, of tergite 7 = 4.3–8.1 mm.

***Colouration*** (Fig. 7A–I). Specimens in life greenish brown or yellowish brown. Thoracic shield and tergites greenish brown, with pale brown posterior bands. Some individuals with two triangular yellow dorsal patches per tergite. Anal shield brown, posterior margin pale brown. Antenna and legs green. Venter pale brown. Specimens in 95% ethanol changed to dark brown after two years of preservation.

***Head*** (Fig. 8B–D). Trapeziform, broad, short; sparsely setose; anterior part and especially mouthparts densely setose. With 66–89 ommatidia (ocelli). Aberrant ommatidium situated at brim of antennal groove. Tömösváry’s organ separated from eye field and located between base of antenna and ommatidia. No sclerotised ridge between antennal groove and ommatidia.

***Antennae*** (Fig. 8B, C, E, F). Short and stout; emerging from sides of head, covered by dense setae; last antennomere reaching back posteriorly to leg pair 3. Lengths of antennomeres in descending order: 6 > 5 > 4 > 3 = 2 = 1. Antennomere 6 flattened, axe-shaped; with a few sensilla basiconica around apical disc. Apical disc flat; with 45–83 apical cones in males, and 41–50 apical cones in females.

***Epipharynx*** (Fig. 8K). Apically with a single triangular central tooth (ct); in the middle with a conspicuous, long, and slender inner tooth; inner area with a single row of triangular fringed spines (rsp); outer area with several pointed and long external teeth (et).

***Gnathochilarium*** (Fig. 8G–J). Lamellae linguales (ll) rectangular, slightly concave; covered by long setae. Central pads (Cp) modified, with two types of sensory cones (sc), first small without tubercle apically, second one larger with a concave pad apically containing a single internal tubercle. Stipites (st) large and thick, with dense and long setae; located laterally to lamellae linguales. Mentum (me) broad and fused, with dense and long setae. Lateral palpi inconspicuous. Inner palpi (ip) with numerous sensory cones (sc) located in a single field, each cone concave apically and containing a single internal tubercle.

***Mandibles* (*gnathal lobe*)** (Fig. 8L). With single undivided external tooth (Et) and 3-combed inner tooth (3it). With 4–5 rows of pectinate lamellae (pl). Inner area (Ia) with several rows of small, slender teeth. Molar plate (mp) relatively flat. Condylus (co) conspicuous; apically with a single ridge.

***Tegument*** (Figs 7A–I, 9). Quite dull; collum, thoracic shield, tergites and anal shield covered by golden setae; each seta emerged from a pit. Anterior margins of tergites and anal shield with fewer setae than posterior one. Posterior rim with bare setae dorsally and ventrally.

***Collum*** (Fig. 8A). Subsemicircular; lateral edges triangular, with sparse setae.

***Thoracic shield***. Anterior margin with shallow canal covered by numerous long setae; with 5–8 conspicuous keels.

***Midbody tergite*** (Fig. 9A–C). Dorso-anterior margin with a row of circular impressions and numerous small tubercles. Inner area with a shallow groove, separated from anterior margin by a row of crenate barriers. Tips of paratergites projected posteroventrad.

***Anal shield*** (Fig. 9E–G). Slightly sexually dimorphic, females well-rounded, males rectangular and slender. Sparsely setose. Anteriorly with a row of circular impressions and numerous small tubercles. Inner area with a shallow groove, separated from margin by a row of crenate barriers. Underside with two dark locking carinae; twice as long as those of tergites when combined.

***Endotergum of thoracic shield and midbody tergite*** (Fig. 22C, D). Thoracic shield slightly different from midbody tergite. Posterior margin (pm) regularly flat in thoracic shield while midbody tergite modified with rectangular projection, length of each projection being longer than height. Both thoracic shield and midbody tergite; outer area (oa) with several small denticles; marginal bristles arranged in 1–2 rows, tips of the longest bristles almost not extending over posterior margin; middle area (ma) with a row of oval cuticular impressions (cp), distance between each impression almost the same as individual diameter; inner area (ia) with a few long setae.

***Pleurite* (*laterotergite*)** (Fig. 9H, I). Densely setose. First pleurite boomeranglike, tip attenuated and pointed. Pleurite 2 wide, tip more rounded. Pleurites 3–12 broad and wide; flat; rectangular; tip reduced in size, forming a small process.

***Subanal plate of female*** (Fig. 9D). Densely setose; wide; triangular; apical margin round and narrow.

***Stigmatic plates*** (Fig. 10A). First stigmatic plate slender; slightly attenuate; apex round, slightly curved towards coxa. Second stigmatic plate in both sexes swollen; shorter than first one; tip without curve.

***Legs*** (Fig. 10). All podomeres densely setose. Coxa (cx) large; first and second leg-pairs without coxal process; leg-pairs 3–21 with serrated processes. Prefemur (pre) rectangular; with short ridge (rdg); apico-mesally with a very weak projection; mesal margin without triangular spines. Femur (fe) long; 1.5 × longer than prefemur; with conspicuous ridge (rdg); apico-mesally with a strong projection; mesal margin dentate, with 12–16 small triangular microscopic spines. Postfemur (po) and tibia (ti) short. Tarsus (ta) of midbody legs long and slender, as long as length of femur+postfemur; first three leg-pairs without an apical spine; leg-pair 1 with one or two ventral spines; leg-pair 2 with two or three ventral spines; leg-pair 3 with 3–5 ventral spines; leg pair 4 and 5 with five or six ventral spines and one or two apical spines; leg pairs 6–18 with 6–8 ventral spines and two apical spines; leg pair 19–21 with 5–7 ventral spines and one or two apical spines. In leg 10, femur 1.5 × and tarsus 2.5 × longer than wide. Claw normal; with small notch at base.

***Male sexual characters*** (Fig. 10B). Gonopore triangular; covered with an undivided, triangular, sclerotised plate. Apical part of coxa 2 without membranous process.

***Anterior telopods*** (Figs 11A, 11C, 11F, 11G, 23F–H). Consisting of four telopoditomeres. First telopoditomere rectangular; slightly longer than wide. Telopoditomere 2 large. Process of telopoditomere 2 (= immovable finger) wide; shorter than movable finger (= combination of telopoditomeres 3 and 4); clearly seen in posterior view; strongly curved toward movable finger; inner margin with a single membranous lobe and numerous sclerotised spots (scl-s). Telopoditomere 3 slender; almost twice as long as telopoditomere 4; clearly demarcated from telopoditomere 4 by shallow suture; inner margin with tiny single spine; apically with four or five crenulated teeth (cr-t). Telopoditomere 4 short; tip round, with two conspicuous spines. All telopoditomeres covered by long sparse setae, except for basal part of telopoditomere 1 and tip of telopoditomere 2.

***Posterior telopods*** (Figs 11A–E, 11G, 23I, 23J). Consisting of four telopoditomeres. First telopoditomere rectangular; stout; slightly wider than long. Telopoditomere 2 large and wide. Process of telopoditomere 2 (= immovable finger) long and swollen, twice as long as wide; slightly longer than a combination of telopoditomeres 3 and 4 (= movable finger); rounded apically; slightly curved; inner margin with two membranous lobes (ml) and numerous conspicuous sclerotised spots (scl-s). Telopoditomere 3 long and slender; twice as long as wide; inner margin with a large membranous ledge and single long spine; posteriorly with a row of 11–13 crenulated teeth (cr-t). Telopoditomere 4 quite short; 3.5 × shorter than telopoditomere 3; twice as long as wide tip; inner margin with two long spines located on membranous ledge. Telopoditomeres 1 and 2 sparsely setose on anterior surface, glabrous on posterior surface. Telopoditomere 3 sparsely setose. Telopoditomere 4 glabrous. Inner horns densely setose; slightly attenuate near tip.

***Female sexual characters*** (Fig. 10C). Vulva large, sparsely setose; covering almost 1/3 of coxa; located mesoventrally; mesally reaching to base of prefemur. Operculum (op) large; well-rounded; with rounded tip. Bursa (bu) wide; fused; separated from operculum by shallow triangular groove. Apical part of coxa 2 without coxal process.

##### Remarks.

Noticeable variations occur in colouration and patterns of tergites. Most specimens display two yellow patches on dorsal tergites, typically semicircular or rectangular in shape (Fig. 7A, E). However, in some individuals, these patches may fade to a paler hue or completely absent (Fig. 7A, H).

The Thai specimens differ from the original description by Wesener (2019) in some morphological features. They have a greater range of body sizes, with males usually being smaller and females slightly larger, and they vary more in width and height. The tergites feature triangular yellow patches instead of the bright green of the type material. The aberrant ocellus of the Thai specimens is situated close to the brim of the antennal groove rather than inside. The number of apical sensory cones varies more widely and is greater in female Thai specimens (males 45–83; females 41–50). In the original description, the posterior margin of the endotergum was flat, but in Thai specimens, it was modified. The tegument of Thai specimens is dull and densely covered in golden setae, in contrast to the type specimens, which have a smooth, shiny, and glabrous surface (T. Wesener, pers. comm., re-examination of the type material).

In contrast to the type specimens, the paratergites are more noticeably protruding posteroventrad, and the thoracic shield comprises 5–8 keels. The legs of the Thai specimens differ slightly from what was originally described. Thai specimens possessed 12–16 small triangular spines on their femurs, while no spines were mentioned in the original description. Because these spines are minute and the original description was based on light microscopy only, their absence from Wesener (2019) most probably reflects the limits of that method rather than a genuine difference between the populations. The tarsus has somewhat reduced number of ventral and apical spines and is correspondingly shorter (2.5 × versus 3.5 × broad). In contrast to the original description, females have a smaller vulva that only covers one-third of the coxa, and the subanal plate is narrowly pointed rather than well-rounded. In males, the gonopore remains similar, but the anterior telopods differ by telopoditomere 4 with crenulated teeth. The immovable fingers of posterior telopods of Thai specimens are slender with 11–13 crenulated teeth, whereas those of the type specimens bear 11. Overall, the Thai specimens show consistent differences in body size, colour pattern, tegument texture, setation, legs, and telopod morphology, suggesting large intraspecific variation from the originally described population.

Despite this obvious variation, the male anterior and posterior telopods, the principal species-diagnostic structures in *Zephronia*, are indistinguishable between the Thai and Lao material. The Thai sequences cluster with the topotypic sequence MK330977 with maximal support (BS = 100, PP = 1.00) at a divergence of 4.88–5.95%, below the lowest interspecific distance recovered in this study (8.68%). We therefore treat the Thai population as a geographically divergent population of *Z. laotica*, while acknowledging that denser sampling in Laos and nuclear markers may reveal cryptic species-level structure.

##### Distribution and habitat.

*Zephronia laotica* is currently recorded only from the lower northeastern region of Thailand, specifically Si Sa Ket Province (Fig. 24). This species is typically found during the daytime in agricultural habitats such as durian orchards and rubber plantations. Specimens from durian orchards were found buried just below the soil surface, while those from rubber plantations were observed living on the surface beneath the leaf litter or decayed wood (Fig. 7J, K).

#### Zephronia tamarindina

Taxon classification

Animalia

SphaerotheriidaZephroniidae

Wilaisorn & Srisonchai sp. nov.

4B7A84C4-3772-5086-B481-52A2BB679763

https://zoobank.org/8165DAE1-E044-451A-9F0A-412857B3BDA0

Figs 1, 12–16, 22E, F, 23K–O, 24

##### Type specimens.

***Holotype*** • ♂ (MZKKU-MYR0021); Thailand, Phetchabun Province, Bueng Sam Phan District, Nimit Sat Cha Tham Temple; 15°49'16.8"N, 100°55'03.9"E; ca 204 m a.s.l.; 3 September 2023; leg. C. Wilaisorn and MZKKU students. ***Paratypes***. • 4 ♂♂ 4 ♀♀ (MZKKU-MYR0022); same data as for holotype; • 1 ♂ 1 ♀ (CUMZ-MYR0041); same data as for holotype.

##### Diagnosis.

The position of the Tömösváry’s organ located at the brim rather than inside the antennal groove (Fig. 13B, C), identifies *Z. tamarindina* sp. nov. as a member of the *Zephronia* s.s. species group. *Z. tamarindina* sp. nov. has an orangish brown body colour in adults and olive-greenish brown colour in juveniles, and differs from all other *Zephronia* s.s. species by the following combination of characters: in the anterior telopods, the process of telopoditomere 2 is broad and only slightly shorter than the combined length of telopoditomeres 3 and 4 (Figs 16C, 23K, 23M); telopoditomere 3 of the anterior telopods is distinctly widened (Figs 16C, 16F–H, 23K–M); and in the posterior telopods, telopoditomere 4 is strongly curved toward the immovable finger (Figs 16A–D, 23N, 23O). Males possess a conspicuous membranous process on the coxa of leg-pair 2 (Fig. 15B), a feature absent in all species except males of *Z. enghoffi*, *Z. golovatchi* and *Z. sukhothaiensis*. *Z. tamarindina* sp. nov. can be distinguished from the first two species by having only one row of marginal bristles in the midbody endotergum (two rows in *Z. enghoffi and Z. golovatchi*), and by the tip of the longest bristle being only half the length of the outer area (Fig. 22E, F), whereas in *Z. enghoffi* and *Z. golovatchi* the longest bristles project beyond the posterior margin. *Z. tamarindina* sp. nov. differs from *Z. sukhothaiensis* in the process of telopoditomere 2 of posterior telopods longer than telopoditomere 3+4 (while that of *Z. sukhothaiensis* is slightly shorter). Genetic divergence of *COI* sequences between *Z. tamarindina* sp. nov. and other *Zephronia* species ranged from 10.81 to 22.07%.

**Figure 12.**
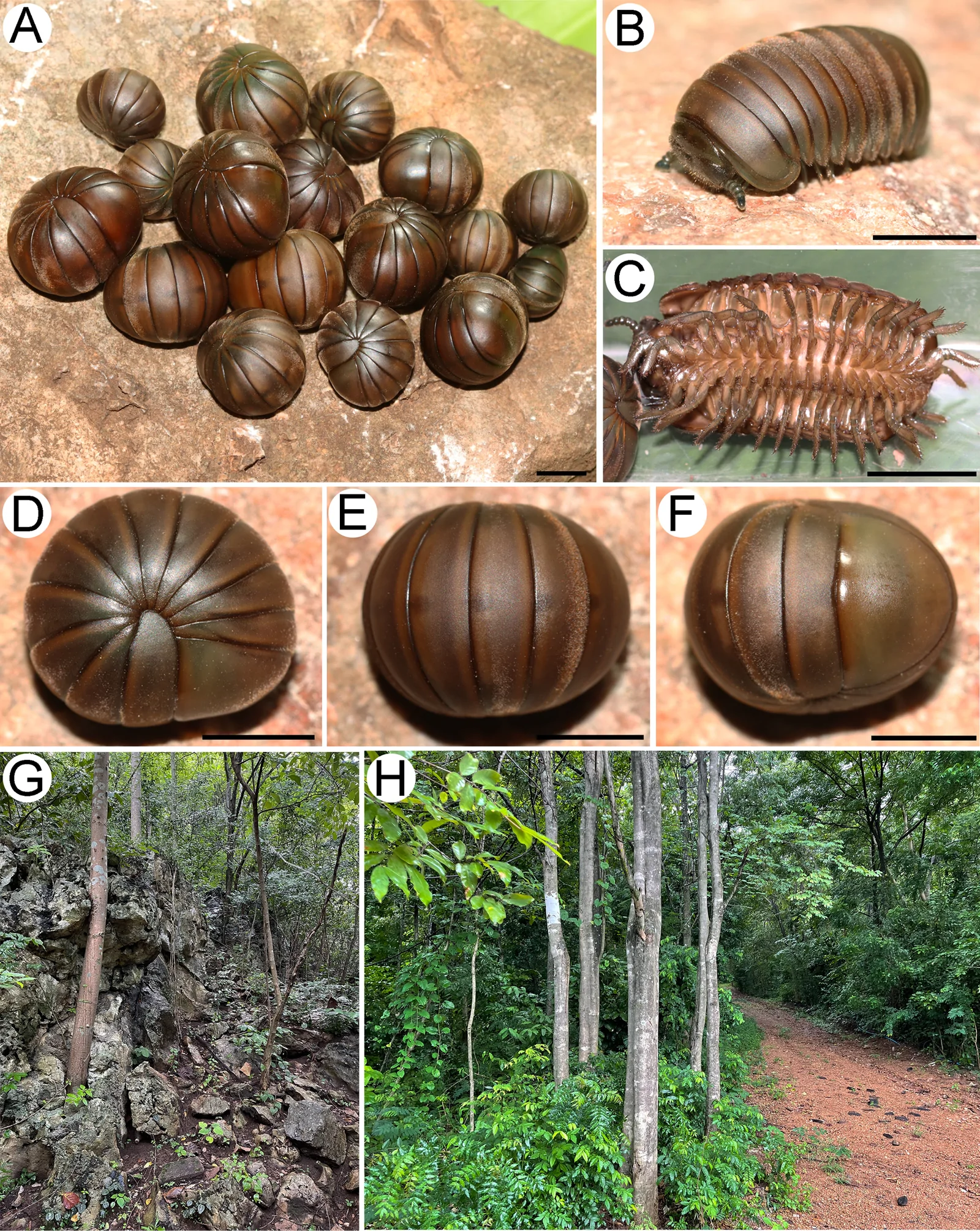
Photograph of live specimens of *Zephronia tamarindina* sp. nov. and habitats. **A–F**. Paratypes (MZKKU); **G, H**. Dipterocarp forest at the type locality. Scale bars: 5.0 mm.

**Figure 13.**
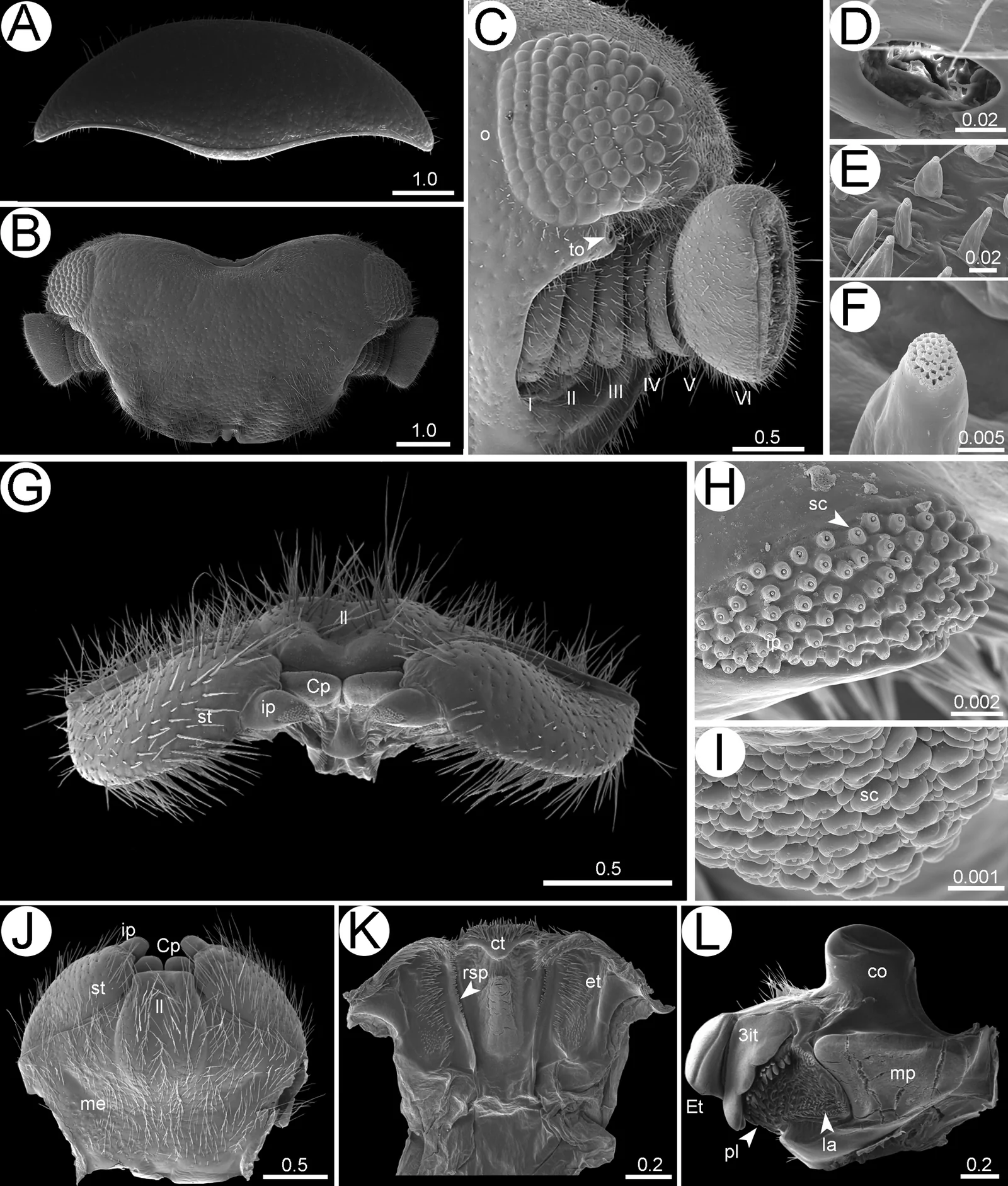
*Zephronia tamarindina* sp. nov., ♂ paratype (MZKKU) — SEM. **A**. Collum, dorsal view; **B**. Head, anterior view; **C**. Antenna, lateral view; **D**. Tömösváry’s organ; **E, F**. Apical cones, lateral view; **G, J**. Gnathochilarium, anterior and ventral views, respectively; **H**. Sensory cones on inner palpi, ventral view; **I**. Sensory cones on central pad, ventral view; **K**. Epipharynx, ventral view; **L**. Right mandible, mesal view. Abbreviations: 3it = 3-combed inner tooth, co = condylus, Cp = central pad, ct = central tooth, et = external tooth of epipharynx, Et = external tooth of mandible, Ia = inner area, il = incisura lateralis, ip = inner palpi, ll = lamellae linguales, me = mentum, mp = molar plate, o = ommatidia, pl = pectinate lamellae, rsp = row of spines, sc = sensory cone, st = stipites, t = Tömösváry’s organ. Scale bars in millimetres.

**Figure 14.**
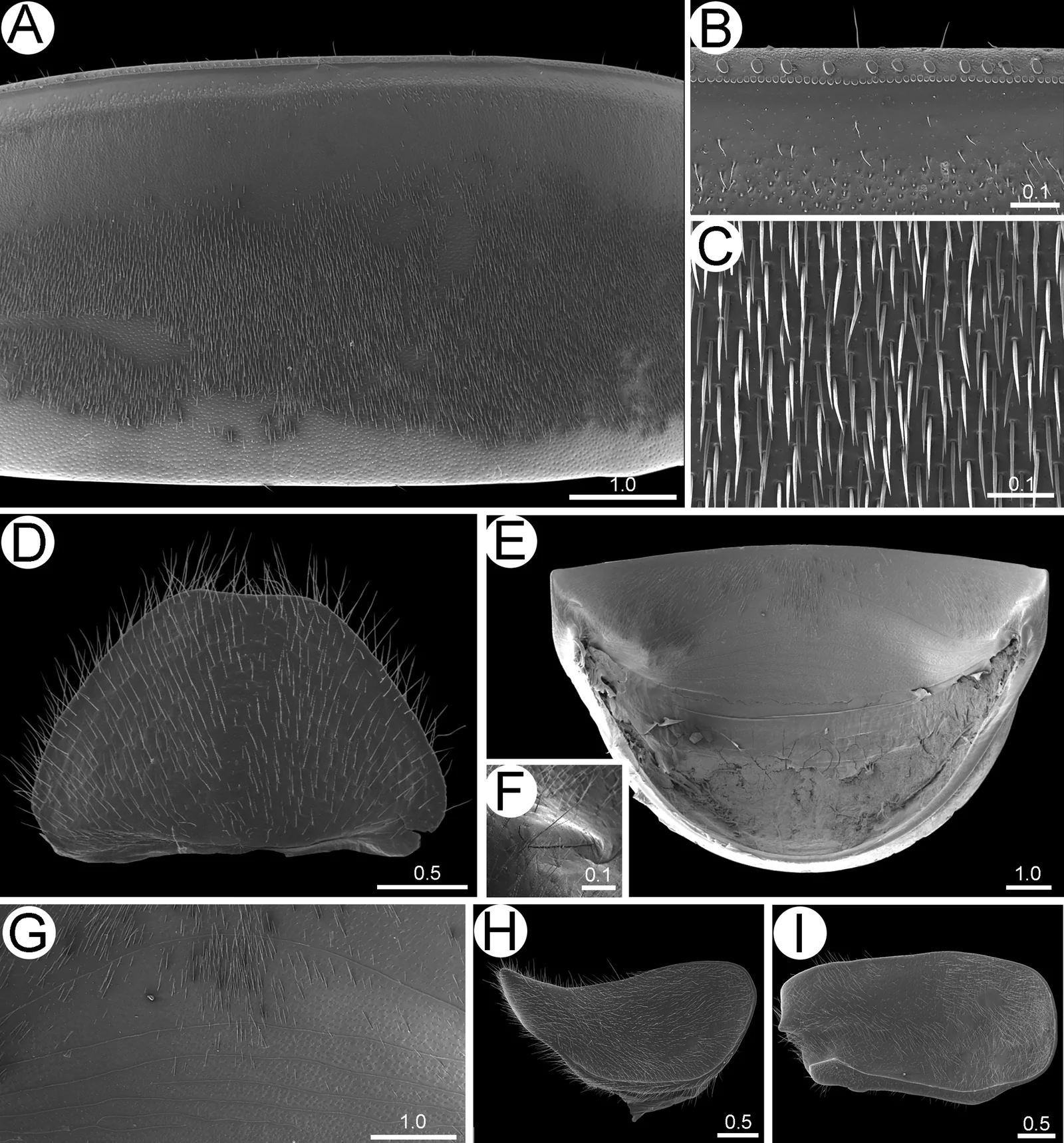
*Zephronia tamarindina* sp. nov., ♂ paratype (MZKKU), ♀ paratype (MZKKU) — SEM. **A–C**. Surface of midbody tergite, all in dorsal views (overview, anterior margin, surface, respectively); **D**. Subanal plate; **E**. Anal shield, underside, ventral view; **F**. Locking carinae; **G**. Surface of anal shield; **H**. First pleurite; **I**. Midbody pleurite. Scale bars in millimetres.

**Figure 15.**
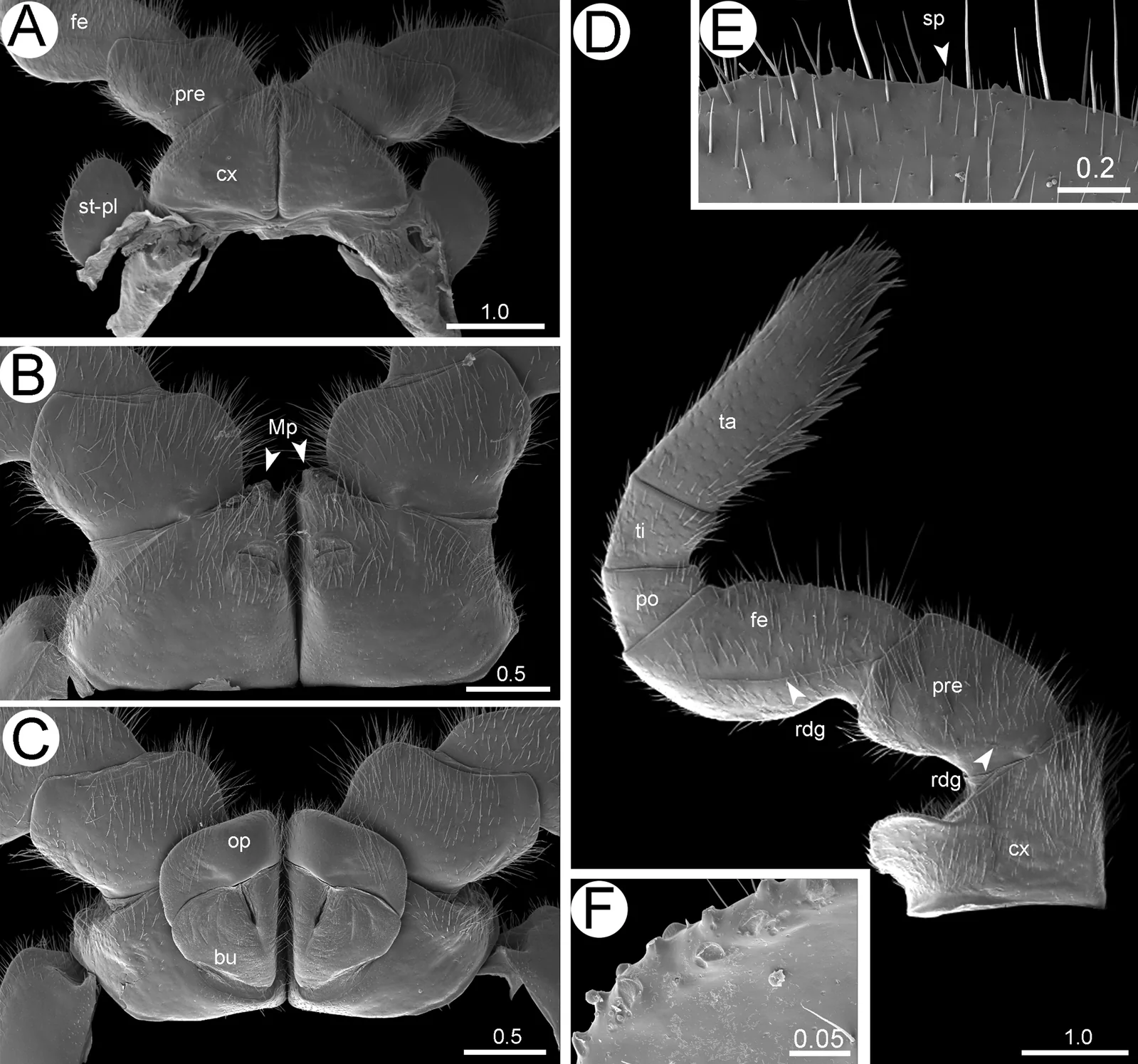
*Zephronia tamarindina* sp. nov., ♂ paratype (MZKKU), ♀ paratype (MZKKU) — SEM. **A**. Legs 1; **B**. Legs 2 with gonopores; **C**. Leg 2 with vulvae; **D**. Midbody legs (arrow points to femoral ridge); **E**. Small triangular spines on femur; **F**. Coxal process on midbody legs. Abbreviation: bu = bursa, cx = coxa, fe = femur, Mp = membranous process, op = operculum, po = postfemur, pre = prefemur, rdg = femoral ridge, st-pl = stigmatic plate, ta = tarsus, ti = tibia. Scale bars in millimetres.

**Figure 16.**
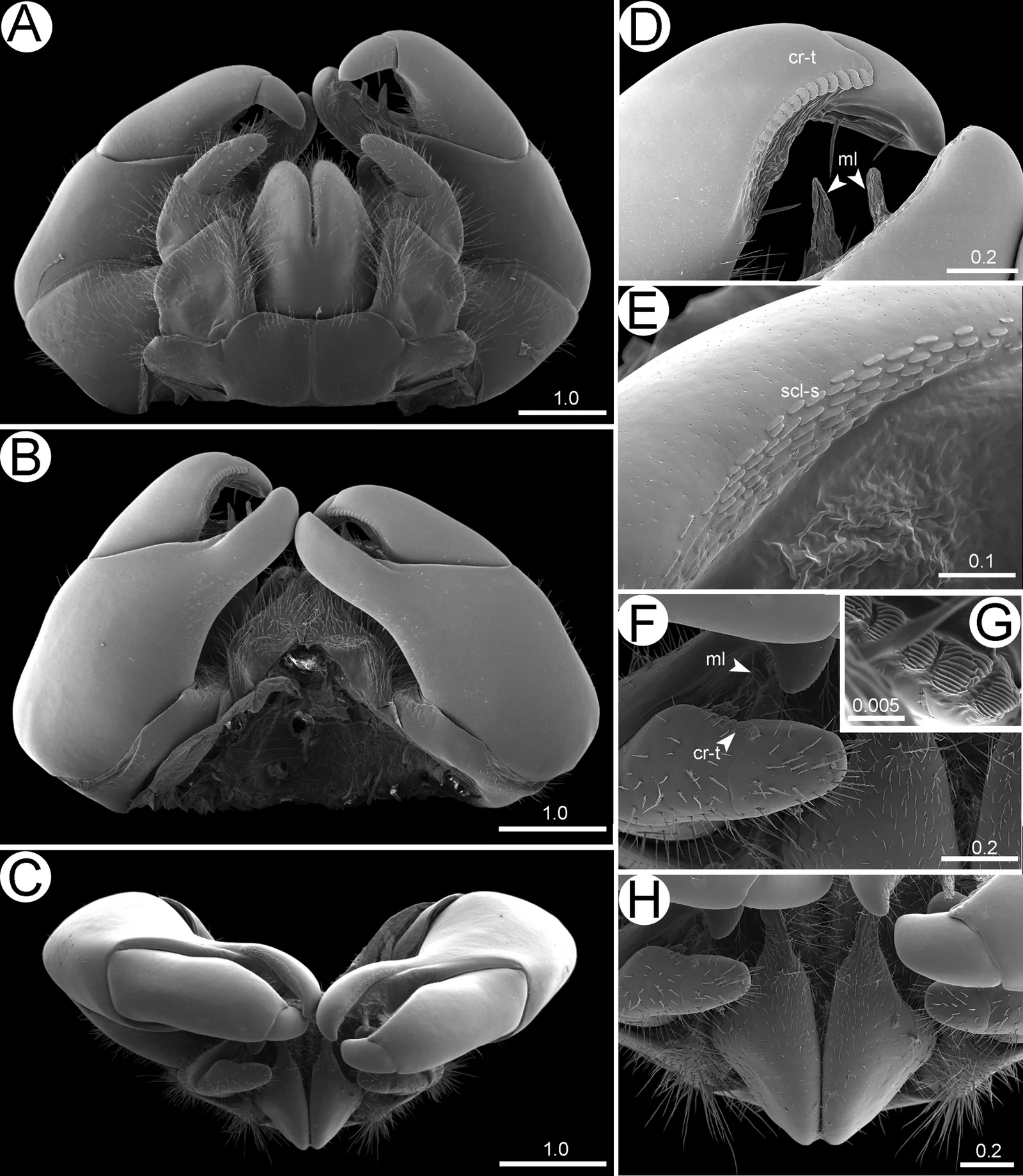
*Zephronia tamarindina* sp. nov., ♂ paratype (MZKKU) — SEM. **A–C**. Overview of telopods (anterior, posterior, and ventral views, respectively); **D**. Crenulated teeth of posterior telopods, posterior view; **E**. Anterior telopod, ventral view; **F**. Crenulated teeth of anterior telopods; **G**. Sclerotised spots on immovable finger of posterior telopod, sublateral view; **H**. Inner horns, anterior view. Abbreviations: cr-t = crenulated teeth, ml = membranous lobe, scl-s = sclerotised spots. Scale bars in millimetres.

##### Description.

***Measurements***. Male holotype; body length = 18.6 mm; width (broadest), of thoracic shield = 9.5 mm, of tergite 7 = 10.5 mm; highest of thoracic shield = 6.1 mm, of tergite 7 = 7.8 mm. Males: body length = 15.0–22.5 mm; width (broadest), of thoracic shield = 7.3–11.7 mm, of tergite 7 = 7.5–12.3 mm; highest of thoracic shield = 5.8–7.6 mm, of tergite 7 = 6.9–8.8 mm. Females: body length = 14.8–26.2 mm; width (broadest), of thoracic shield = 7.1–12.6 mm, of tergite 7 = 6.6–13.7 mm; highest of thoracic shield = 4.8–8.7 mm, of tergite 7 = 8.8–10.0 mm.

***Colouration*** (Fig. 12A–F). Live adult specimens with orangish brown body colour, juveniles olive-greenish brown. Head and collum olive green. Thoracic shield and tergites brown, with pale brown posterior bands (juveniles with two pale yellow rectangular dorsal patches per tergite); paratergites olive green or orangish brown. Roll-up form with a dark brown stripe on dorsal surface of tergites. Anal shield orangish brown with pale posterior brown band. Antenna green. Legs greenish brown. Venter pale brown. Specimens preserved in 95% ethanol changed to dark brown and some yellowish brown after 1.5 years of preservation.

***Head*** (Fig. 13B–D). Trapeziform; broad; concave dorso-posteriorly; densely setose anteriorly, especially mouthparts. With 103–115 ommatidia (ocelli). Aberrant ommatidium situated at brim of antennal groove. Tömösváry’s organ separated from eye field and located between base of antenna and ommatidia. No sclerotised ridge between antennal groove and ommatidia.

***Antennae*** (Fig. 13B, C, E, F). Apparently short and stout, emerging from sides of the head, covered by short setae; last antennomere reaches back posteriorly to leg pair 2. Lengths of antennomeres in descending order: 6 > 5 = 4 > 3 = 2 = 1. Antennomere 6 slightly flat, axe-shaped; with sensilla basiconica around apical disc. Apical disc with 65–88 apical cones in males, 55–67 apical cones in females.

***Epipharynx*** (Fig. 13K). Apically with a single triangular central tooth (ct); with a conspicuous long and slender inner tooth mesally; inner area with a single row of triangular fringed spines (rsp); outer area with several pointed and long external teeth (et).

***Gnathochilarium*** (Fig. 13G–J). Lamellae linguales (ll) oval; apically concave; with dense and long setae. Central pads (cp) modified; with two types of sensory cones (sc); first small, without tubercle apically, second larger with a concave pad apically containing a single internal tubercle. Stipites (st) large, with dense and long setae; located laterally to lamellae linguales. Mentum (me) broad, fused; with dense setae. Lateral palpi inconspicuous. Inner palpi (ip) with numerous sensory cones (sc); located in single field, each cone concave apically and containing a single internal tubercle.

***Mandibles* (*gnathal lobe*)** (Fig. 13L). With single undivided external tooth (Et) and 3-combed inner tooth (3it). With 5–6 rows of pectinate lamellae (pl). Inner area (Ia) with several rows of small slender teeth. Molar plate (mp) relatively flat, with shallow fringe. Condylus (co) conspicuous, apically with two ridges.

***Tegument*** (Figs 12A–F, 14). Dull; collum, thoracic shield, tergites and anal shield covered by golden setae; each seta located in a small pit. Tergites and anal shield sparsely setose anteriorly and densely setose posteriorly.

***Collum*** (Fig. 13A). Subsemicircular; lateral margin with a few long setae.

***Thoracic shield***. Anterior margin with a shallow canal covered by numerous long setae; with 5–9 conspicuous keels.

***Midbody tergite*** (Fig. 14A–C). Dorso-anterior margin with a row of oval circular impressions and numerous small tubercles. Inner area with a shallow groove; separated from anterior margin by a row of crenate barriers. Tips of paratergites projected posteroventrad.

***Anal shield*** (Fig. 14E–G). Slightly sexually dimorphic; females well-rounded, males rectangular and slender. Anteriorly with a row of circular impressions and numerous small tubercles. Inner area with a shallow groove; separated from margin by a row of crenate barriers. Underside with a dark locking carina; as long as those of tergites. Densely setose on both underside and upperside.

***Endotergum of thoracic shield and midbody tergite*** (Fig. 22E, F). Thoracic shield slightly different from midbody tergite. Posterior margin (pm) of both thoracic shield and midbody tergite flat, unmodified. Outer area (oa) of both thoracic shield and midbody tergite with small denticles. Marginal bristles of both thoracic shield and midbody tergite arranged in one row; bristle quite short; tips of longest bristles half-length of outer area and not reaching to posterior margin. Middle area (ma) of thoracic shield with a row of oval cuticular impressions (cp); distance between each impression as half as the length of individual diameter. Middle area of midbody tergite with a row of cuticular impressions; distance between each impression as long as individual diameter. Inner area (ia) of thoracic shield and midbody tergite with a few setae.

***Pleurite* (*laterotergite*)** (Fig. 14H, I). Densely setose. First pleurite triangular; boomeranglike; reaching towards paratergite. Pleurite 2 wider than pleurite 1; tip obtuse. Pleurites 3–12 rectangular.

***Subanal plate of female*** (Fig. 14D). Densely setose; wide; triangular; tip truncated.

***Stigmatic plates*** (Fig. 15A). First stigmatic plate subtriangular; quite slender; flat antero-posteriorly; apex round, curving towards coxa. Second stigmatic plate in both sexes swollen; weakly curved.

***Legs*** (Fig. 15). All podomeres densely setose. Coxa (cx) large; as long as length of prefemur (pre); first and second leg-pairs without coxal process; leg-pairs 3–21 with conspicuous processes. Prefemur stout; with short ridge (rdg); apico-mesally without projection. Femur (fe) wide and long, 1.5 × longer than prefemur; with conspicuous ridge; apico-mesally with a small projection; mesal margin dentate, with 6–8 small triangular microscopic spines (sp). Postfemur (po) and tibia (ti) short. Tarsus (ta) of midbody legs long and slender, as long as length of femur+postfemur; first two leg-pairs without an apical spine; leg-pair 1 with two or three ventral spines; leg-pair 2 with four or five ventral spines; leg-pair 3 with 6–10 ventral spines and one or two apical spines; leg pair 4 with 8–10 ventral spines and two or three apical spines; leg pairs 5–19 with 8–11 ventral spines and 3–5 apical spines; leg pair 20 and 21 with 7–9 ventral spines and three apical spines. In leg 10, femur 1.5 ×, tarsus 3 × longer than wide. Claw normal, with small notch at base.

***Male sexual characters*** (Fig. 15B). Gonopore quite small; covered by a divided, triangular sclerotised plate. Apical part of coxa 2 with membranous process (Mp).

***Anterior telopods*** (Figs 16A, 16C, 16F–H, 23K–M). Consisting of four telopoditomeres. First telopoditomere conspicuous; rectangular; longer than wide. Telopoditomere 2 large. Process of telopoditomere 2 (= immovable finger) broad; only slightly shorter than combination of telopoditomeres 3 and 4 (= movable finger); clearly seen in posterior view, strongly curved toward movable finger; inner margin with a single membranous lobe and numerous sclerotised spots (scl-s). Telopoditomere 3 wide; 2 × longer than telopoditomere 4; clearly demarcated from telopoditomere 4 by shallow suture; inner margin with single inconspicuous spine; apically with six or seven crenulated teeth (cr-t). Telopoditomere 4 very short; tip obtuse, with two spines. All telopoditomeres densely setose, except for basal part of telopoditomere 1 and of immovable finger of telopoditomere 2.

***Posterior telopods*** (Figs 16A–E, 23N, 23O). Consisting of four telopoditomeres. First telopoditomere broad; slightly wider than long. Telopoditomere 2 large. Process of telopoditomere 2 (= immovable finger) long; 2 × longer than wide; longer than a combination of telopoditomeres 3 and 4 (= movable finger); apically round; slightly curved; inner margin with two membranous lobes (ml) and numerous conspicuous sclerotised spots (scl-s). Telopoditomere 3 long and slender; 2.5 × longer than wide; with a large membranous ledge and single long spine at inner margin; posteriorly with a row of 14–20 crenulated teeth (cr-t). Telopoditomere 4 short; 3 × shorter than telopoditomere 3; twice as long as wide; strongly curved toward the immovable finger; inner margin with two long spines located on a membranous ledge. Telopoditomere 1 covered by long setae. Anterior surface of telopoditomere 2 with a few setae but glabrous in posterior one. Inner margin of immovable finger and telopoditomere 3 sparsely setose. Telopoditomere 4 glabrous. Inner horns densely setose; swollen at base, slightly attenuate near tip.

***Female sexual characters*** (Fig. 15C). Vulva large; sparsely setose; covering almost half of coxa; located at mesal margin; extending mesally to base of prefemur. Operculum (op) large; rectangular; mesal margin slightly concave; tip round. Bursa (bu) fused; heart-like; separated from operculum by a narrow triangular groove. Apical part of coxa 2 without coxal process.

##### Remarks.

The variation in colouration of paratergites was observed, with most specimens exhibiting an olive green colour, while some appeared orangish brown. Juveniles and smaller individuals typically showed olive greenish-brown colouration with rectangular patches on tergites. A distinctive feature of this species is the conspicuous triangular membranous process present on the coxae of male leg-pair 2 (Fig. 15B).

##### Distribution and habitat.

*Zephronia tamarindina* sp. nov. is currently known only from its type locality in Phetchabun Province and is thus considered endemic to this area (Fig. 24). The species was encountered during daytime on a rainy day within a dipterocarp forest, characterised by limestone terrain (Fig. 12G, H). Most specimens were observed moving on the ground amid gravel with sparse leaf litter.

##### Etymology.

The name is an adjective that derives from *Tamarindus* (tamarind), an important fruit cultivated in Phetchabun Province, the type locality. The name is treated as a feminine adjective in reference to the species body colouration, which changes from pale olive green in juveniles to a darker orangish brown in adults, resembling the ripening process of tamarind fruit from green to brown.

#### Zephronia triforma

Taxon classification

Animalia

SphaerotheriidaZephroniidae

Wilaisorn & Srisonchai sp. nov.

49AC5F67-AB78-5D73-AB8B-52D798D5B81D

https://zoobank.org/1B0E385B-F7DC-4EBF-9DC5-18F8D6652244

Figs 1, 17–21, 22G, H, 23P–T, 24

##### Type specimens.

***Holotype*** • ♂ (MZKKU-MYR0023); Thailand, Phitsanulok Province, Wat Bot District, Ban Nam Kob; 16°59'43.6"N, 100°23'34.0"E; ca 95 m a.s.l.; 6 August 2024; leg. C. Wilaisorn and MZKKU students. ***Paratypes***. • 29 ♂♂ 25 ♀♀ (MZKKU-MYR0024), same data as for holotype; • 1 ♂ 1 ♀ (CUMZ-MYR0042); same data as for holotype; • 1 ♂ 1 ♀ (NHMUK); same data as for holotype; • 1 ♂ 1 ♀ (NHMD); same data as for holotype; • 1 ♂ 1 ♀ (NHMW); same data as for holotype; • 2 ♂♂ 2 ♀♀ (ZFMK); same data as for holotype.

##### Additional material.

• 13 juveniles (MZKKU), same data as for holotype; • 29 ♂♂, 13 ♀♀ (MZKKU); Thailand, Phitsanulok Province, Wat Bot District, Khao Bo Ma Viewpoint; 17°18'20.3"N, 100°26'29.2"E; ca. 216 m a.s.l.; 10 July 2024; leg. C. Wilaisorn, R. Srisonchai and MZKKU students; • 7 ♂♂ 4 ♀♀ (MZKKU); Thailand, Nakhon Sawan Province, Takhli District, Buddhawajana Dhamma Retreat; 15°17'51.7"N, 100°22'24.6"E; ca 70 m a.s.l.; 31 July 2023; leg. C. Wilaisorn, R. Srisonchai, and MZKKU students.

##### Diagnosis.

The position of the Tömösváry’s organ is located at the brim rather than inside the antennal groove (Fig. 18B, C), placing *Z. triforma* sp. nov. within the *Zephronia* s.s. species group. *Z. triforma* sp. nov. exhibits a distinctive range of live body colour patterns, forming three morphotypes: (1) males with greyish brown colouration and dark brown freckles, (2) females with reddish brown colouration, and (3) both sexes with a brownish emerald-green colouration. Beyond these colour traits, *Z. triforma* sp. nov. differs from other members of the *Zephronia* s.s. group by the following combination of characters: anterior telopods with the process of telopoditomere 2 broad; telopoditomere 4 very short with a tapering tip (Figs 21C, 21F, 23P–R); operculum with a concave mesal margin and a dull tip (Fig. 20C); endotergum with a flat, regular, posterior margin bearing two rows of marginal bristles, the longest bristles not projecting beyond the posterior margin, one row of circular cuticular impressions, and an inner area lacking setae (Fig. 22G, H). Genetic divergence between *Z. triforma* sp. nov. and other *Zephronia* species ranged from 10.81 to 22.83% based on 658 bp *COI* sequences.

**Figure 17.**
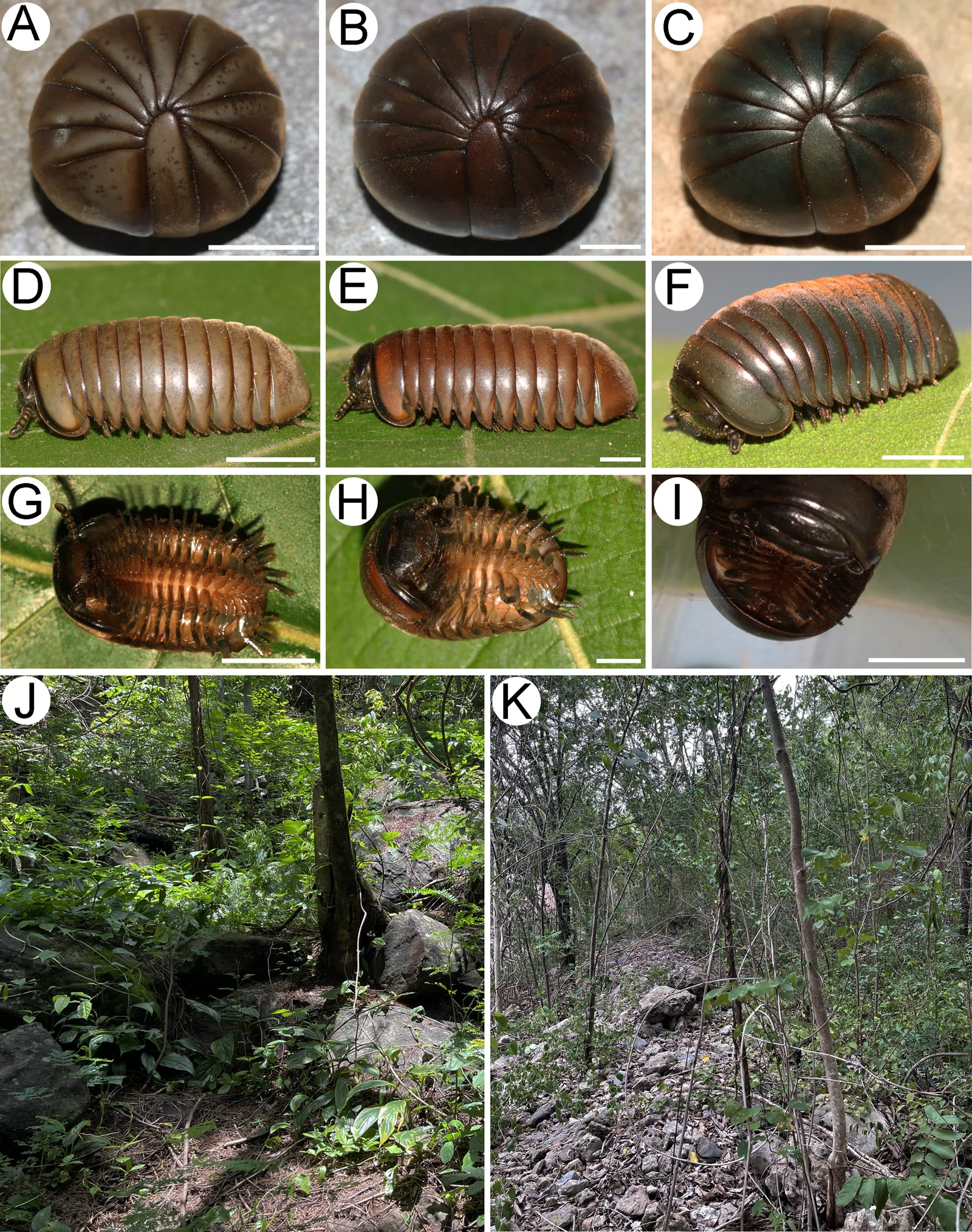
Photograph of live specimens of *Zephronia triforma* sp. nov. and habitats. **A, D, G**. Male paratypes (MZKKU); **B, E, H**. Female paratypes (MZKKU); **C, F, I**. Live specimens of a population from Nakhon Sawan Province (MZKKU); **J**. Dipterocarp forest at the type locality; **K**. Dry deciduous forest at Nakhon Sawan sampling area. Scale bars: 5.0 mm.

**Figure 18.**
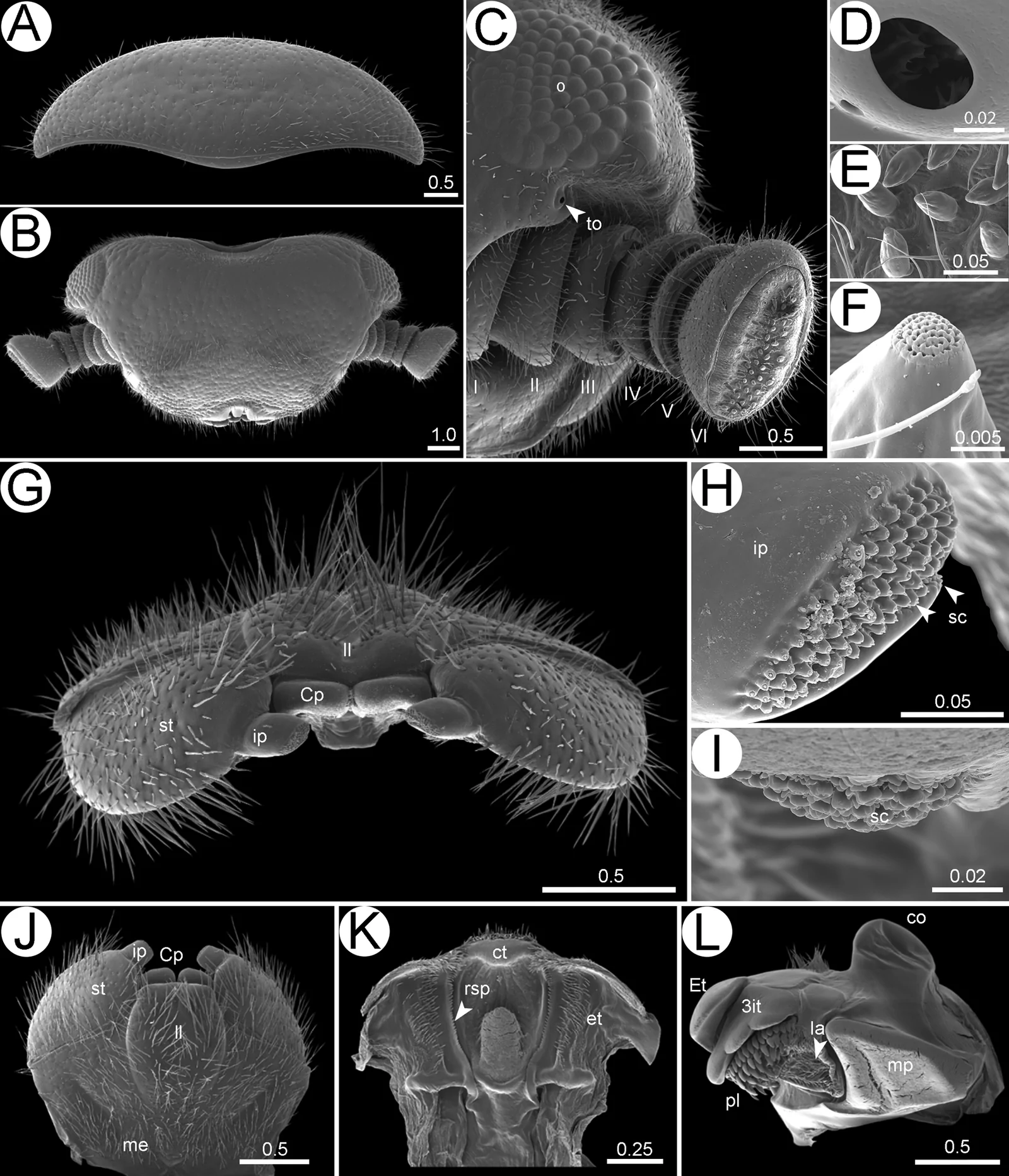
*Zephronia triforma* sp. nov., ♂ paratype (MZKKU) — SEM. **A**. Collum, dorsal view; **B**. Head, anterior view; **C**. Antenna, lateral view; **D**. Tömösváry’s organ; **E, F**. Apical cones, lateral view; **G, J**. Gnathochilarium, anterior and ventral views, respectively; **H**. Sensory cones on inner palpi, ventral view; **I**. Sensory cones on central pad, ventral view; **K**. Epipharynx, ventral view; **L**. Right mandible, mesal view. Abbreviations: 3it = 3-combed inner tooth, co = condylus, Cp = central pad, ct = central tooth, et = external tooth of epipharynx, Et = external tooth of mandible, Ia = inner area, il = incisura lateralis, ip = inner palpi, ll = lamellae linguales, me = mentum, mp = molar plate, o = ommatidia, pl = pectinate lamellae, rsp = row of spines, sc = sensory cone, st = stipites, t = Tömösváry’s organ. Scale bars in millimetres.

**Figure 19.**
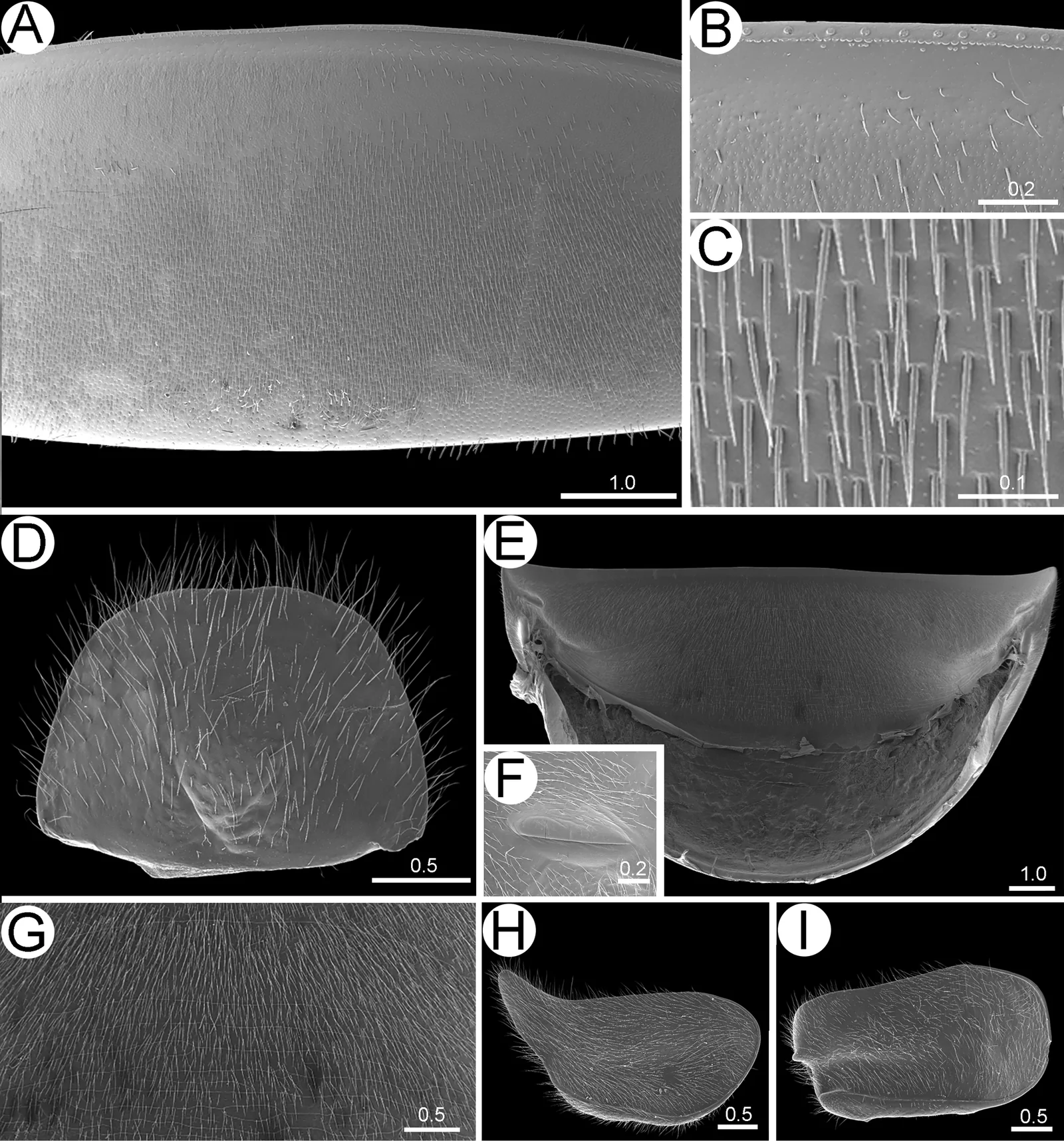
*Zephronia triforma* sp. nov., ♂ paratype (MZKKU), ♀ paratype (MZKKU) — SEM. **A–C**. Surface of midbody tergite, all in dorsal views (overview, anterior margin, surface, respectively); **D**. Subanal plate; **E**. Anal shield, underside, ventral view; **F**. Locking carinae; **G**. Surface of anal shield; **H**. First pleurite; **I**. Midbody pleurite. Scale bars in millimetres.

**Figure 20.**
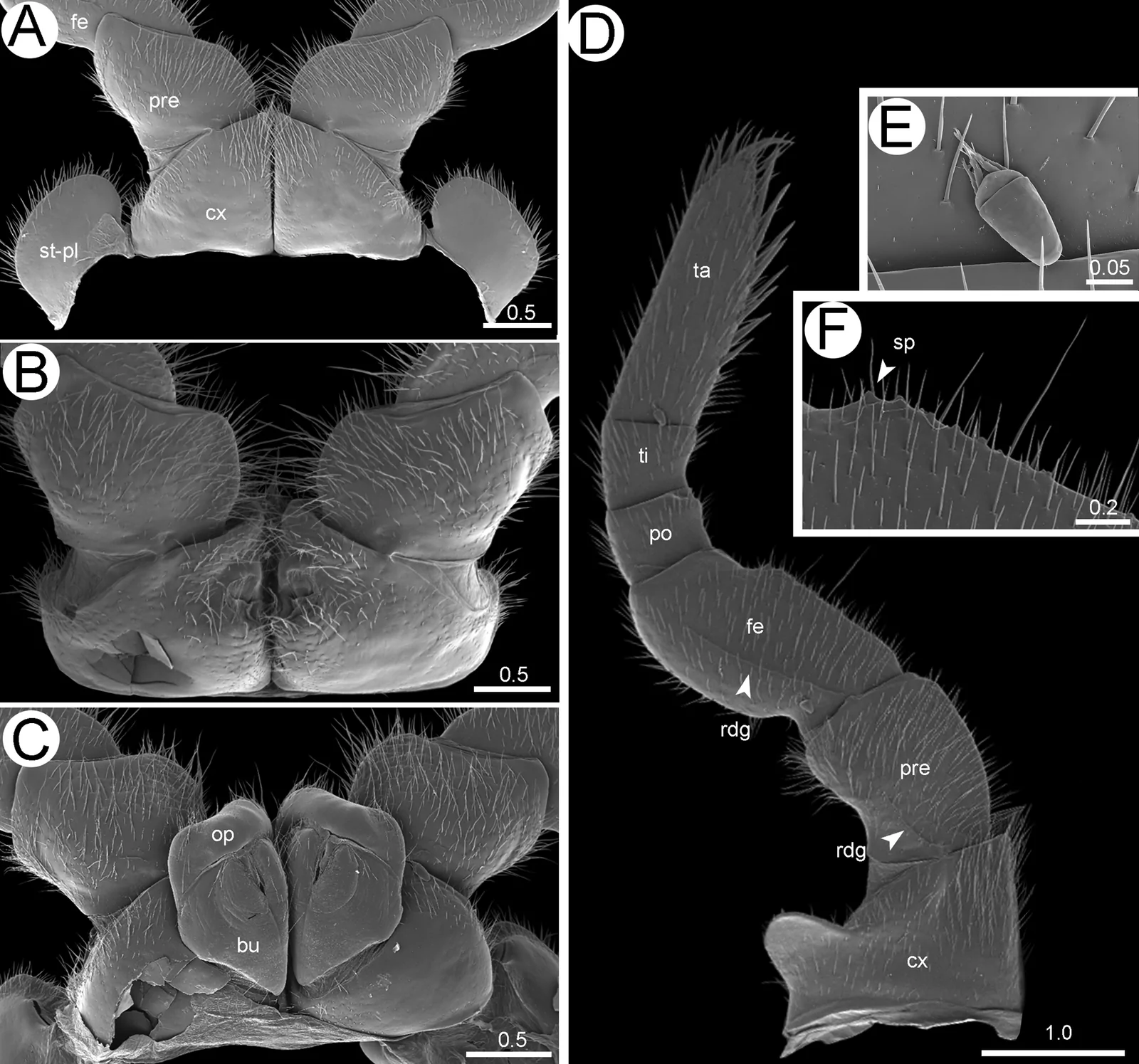
*Zephronia triforma* sp. nov., ♂ paratype (MZKKU), ♀ paratype (MZKKU) — SEM. **A**. Legs 1; **B**. Legs 2 with gonopores; **C**. Leg 2 with vulvae; **D**. Midbody legs (arrow points to femoral ridge); **E**. Phoretic deutonymphs of an unknown mite; **F**. Small triangular spines on femur. Abbreviation: bu = bursa, cx = coxa, fe = femur, op = operculum, po = postfemur, pre = prefemur, rdg = femoral ridge, st-pl = stigmatic plate, ta = tarsus, ti = tibia. Scale bars in millimetres.

**Figure 21.**
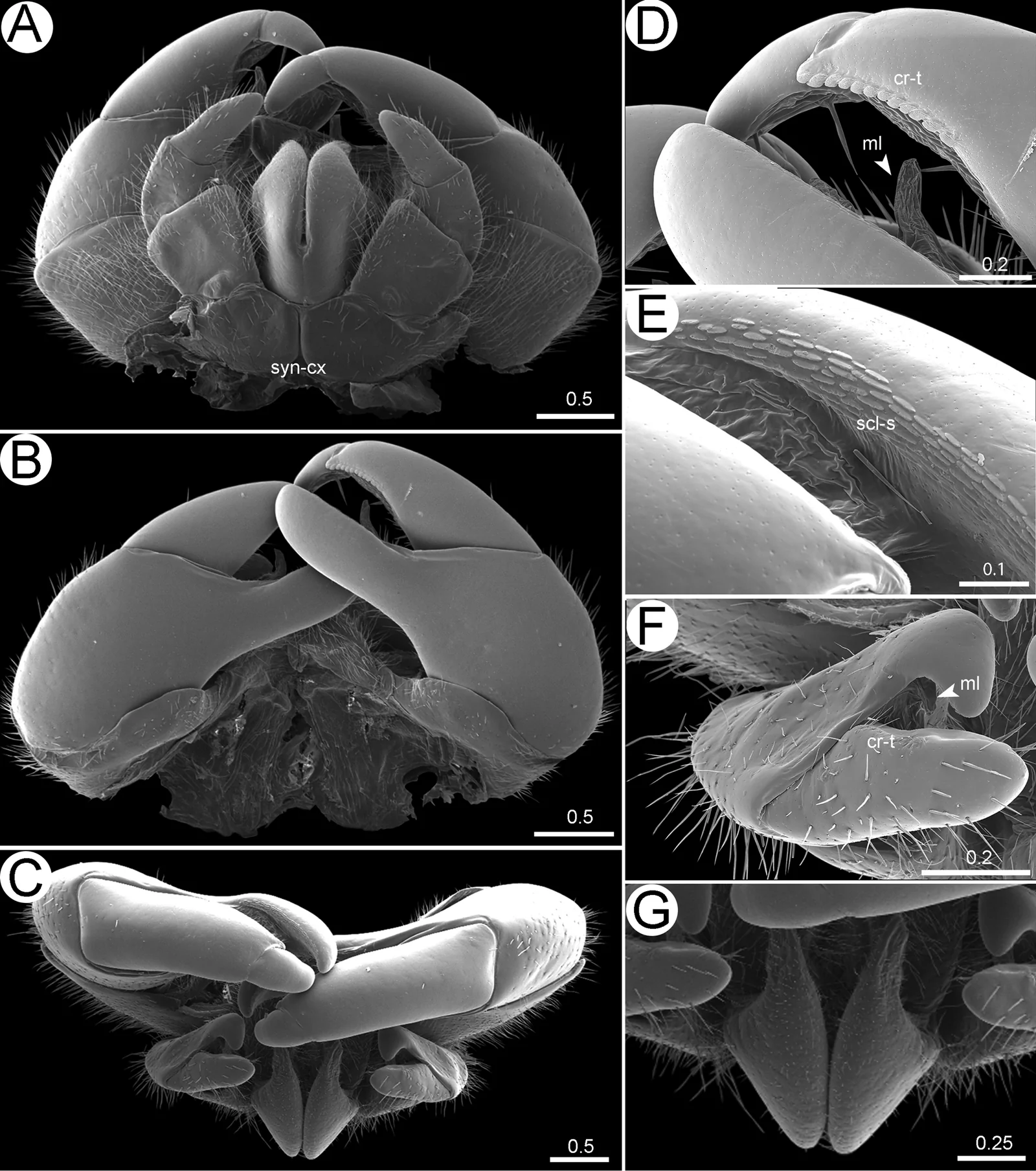
*Zephronia triforma* sp. nov., ♂ paratype (MZKKU) — SEM. **A–C**. Overview of telopods (anterior, posterior, and ventral views, respectively); **D**. Crenulated teeth of posterior telopods, posterior view; **E**. Sclerotised spots on immovable finger of posterior telopod, sublateral view; **F**. Anterior telopod, ventral view; **G**. Inner horns, anterior view. Abbreviations: cr-t = crenulated teeth, ml = membranous lobe, scl-s = sclerotised spots. Scale bars in millimetres.

**Figure 22.**
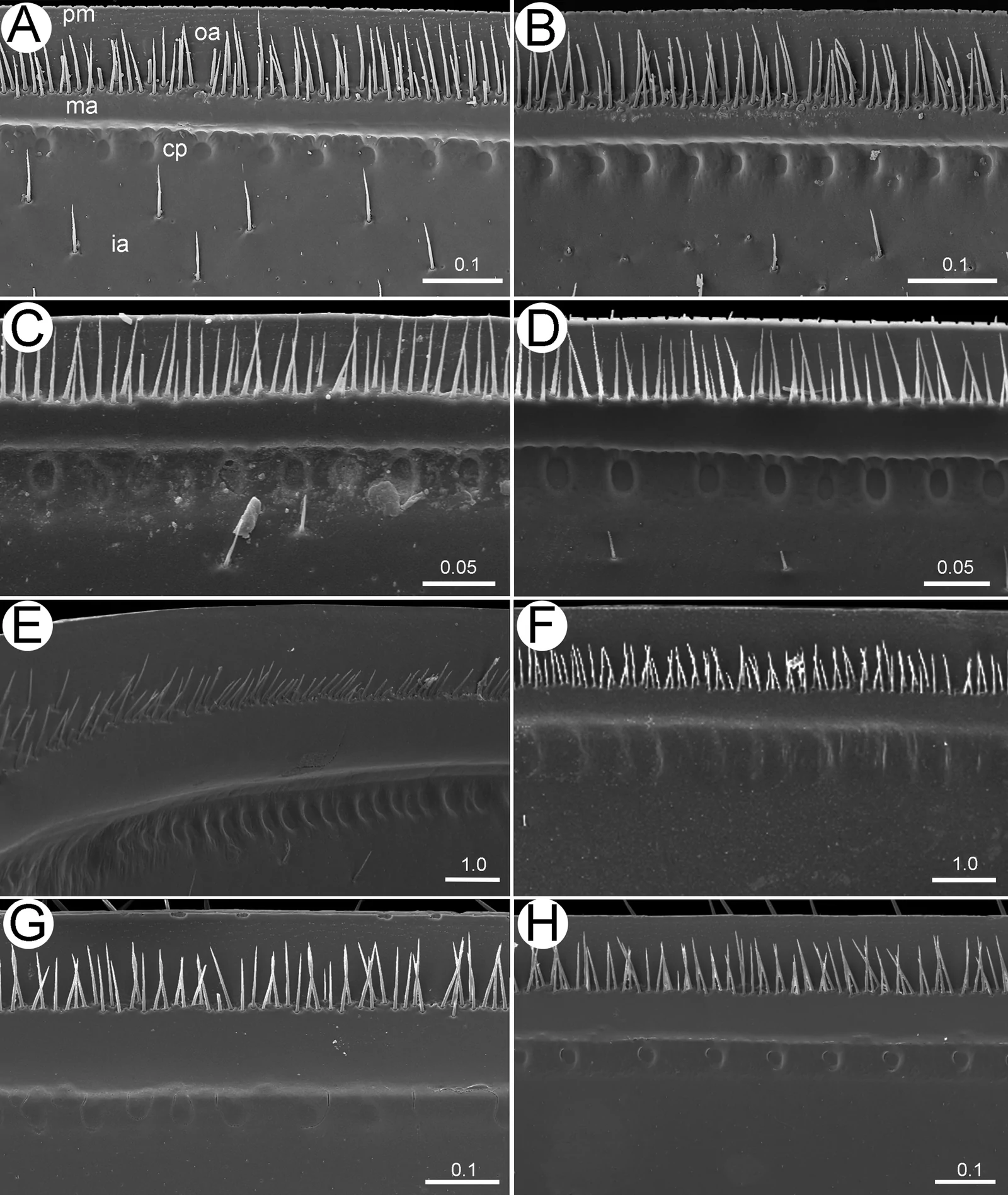
Endoterga of thoracic shields and midbody tergites — SEM. **A, B**. *Zephronia dawydoffi* Attems, 1953 (♂, MZKKU); **C, D**. *Zephronia laotica* Wesener, 2019 (♂, MZKKU); **E, F**. *Zephronia tamarindina* sp. nov. (♂ paratype, MZKKU); **G, H**. *Zephronia triforma* sp. nov. (♂ paratype, MZKKU). Abbreviations: cp = cuticular impression, ia = inner area, ma = middle area, oa = outer area, pm = posterior margin. Scale bars in millimetres.

**Figure 23.**
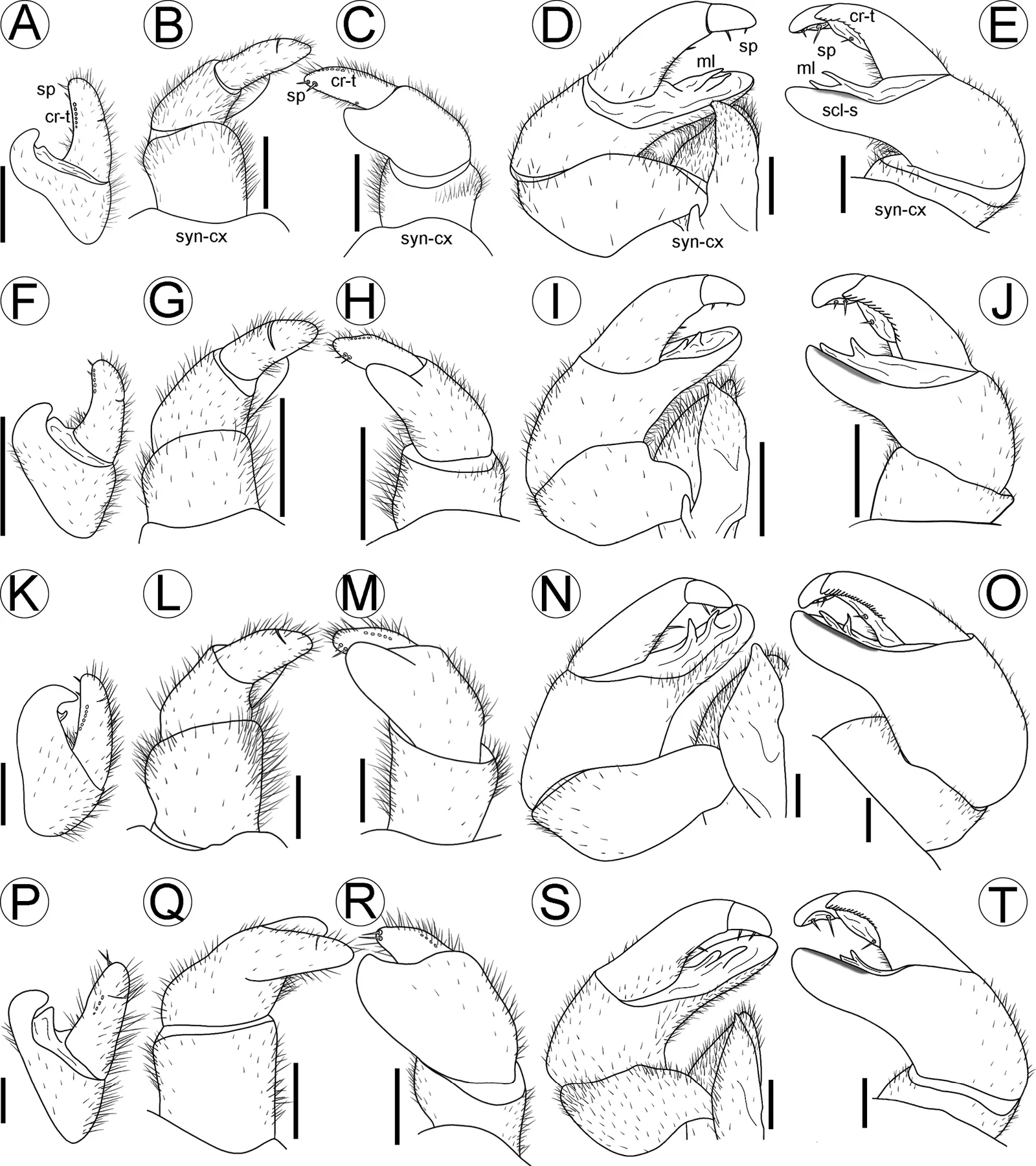
Drawing of telopods of some *Zephronia* species. **A–E**. *Zephronia dawydoffi* Attems, 1953; **F–J**. *Zephronia laotica* Wesener, 2019; **K–O**. *Zephronia tamarindina* sp. nov.; **P–T**. *Zephronia triforma* sp. nov.; **A, F, K, P**. Sublateral view of left anterior telopods; **B, G, L, Q**. Anterior view of left anterior telopods; **C, H, M, R**. Posterior view of left anterior telopods; **D, I, N, S**. Anterior view of left posterior telopods; **E, J, O, T**. Posterior view of left posterior telopods. Abbreviations: cr-t = crenulated teeth, ml = membranous lobe, scl-s = sclerotised spots, syn-cx = syncoxite, sp = spine. Scale bars: 0.5 mm.

**Figure 24.**
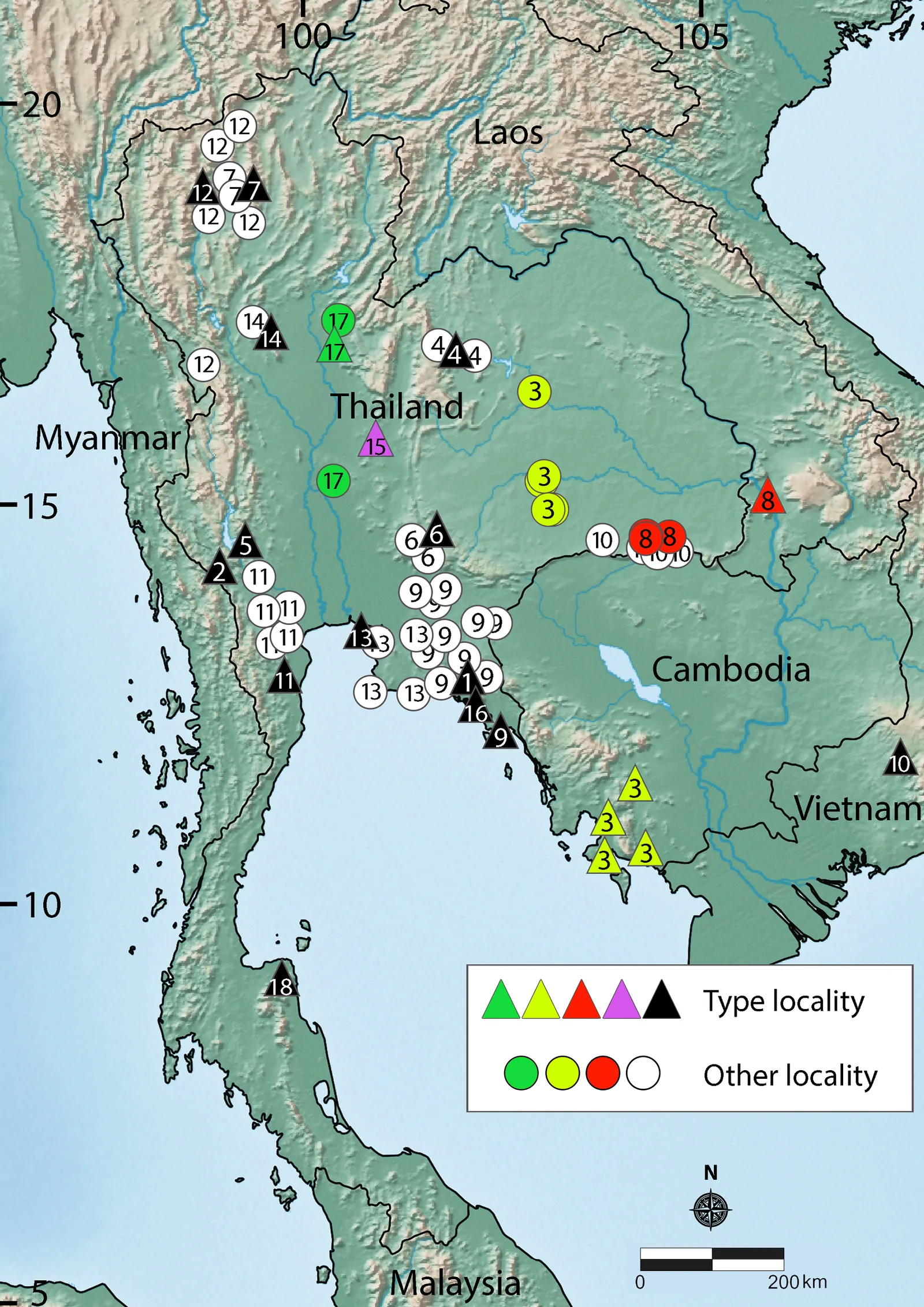
Distribution map of *Zephronia* species in Thailand. Number inside the circle indicates species: 1 = *Z. chantaburiensis* Srisonchai & Wesener, 2024; 2 = *Z. chrysomallos* Bhansali & Wesener, 2022; 3 = *Z. dawydoffi* Attems, 1953; 4 = *Z. enghoffi* Srisonchai & Likhitrakarn, 2021; 5 = *Z. erawani* Bhansali & Wesener, 2022; 6 = *Z. golovatchi* Srisonchai & Likhitrakarn, 2021; 7 = *Z. lannaensis* Likhitrakarn & Golovatch, 2021; 8 = *Z. laotica* Wesener, 2019; 9 = *Z. macula* Srisonchai & Wesener, 2024; 10 = *Z. ovalis* Gray, 1832; 11 = *Z. panhai* Srisonchai & Likhitrakarn, 2021; 12 = *Z. phrain* Likhitrakarn & Golovatch, 2021; 13 = *Z. siamensis* Hirst, 1907; 14 = *Z. sukhothaiensis* Srimongkol & Srisonchai, 2026; 15 = *Z. tamarindina* sp. nov.; 16 = *Z. tratensis* Srimongkol & Srisonchai, 2026; 17 = *Z. triforma* sp. nov.; 18 = *Z. viridisoma* Rosenmejer & Wesener, 2021. Colours (yellow, green, purple, red) indicate specimens examined in this study.

##### Description.

***Measurements***. Male holotype; body length = 17.9 mm; width (broadest), of thoracic shield = 10.6 mm, of tergite 7 = 11.1 mm; highest of thoracic shield = 5.8 mm, of tergite 7 = 6.5 mm. Males: body length = 15.2–26.2 mm; width (broadest), of thoracic shield = 8.7–11.03 mm, of tergite 7 = 9.2–15.2 mm; highest of thoracic shield = 5.2–8.7 mm, of tergite 7 = 5.3–9.7 mm. Females: body length = 18.2–30.8 mm; width (broadest), of thoracic shield = 8.9–15.2 mm, of tergite 7 = 9.6–15.9 mm; highest of thoracic shield = 5.4–9.2 mm, of tergite 7 = 5.1–11.1 mm.

***Colouration*** (Fig. 17A–I). Living specimens of Phitsanulok population of greyish brown colour with dark brown freckles in males; dark reddish-brown colour without freckles in females. Head and collum dark brown. Thoracic shield and tergites greyish brown in males, dark reddish-brown in females. Both sexes with dark brown posterior bands on tergites, and with a dark stripe on middle area of tergites, clearly seen when rolled up. Anal shield orangish brown on posterior margin. Antenna and legs green. Venter pale brown. Specimens in 95% ethanol changed to dark brown after 1.5 years of preservation.

Living specimens of Nakhon Sawan population with a brownish emerald-green body. Head and collum brownish emerald green. Thoracic shield and tergites dark green with dark brown posterior. Anal shield emerald green with pale brown on posterior margin. Antenna and legs green. Venter pale brown. Specimens in 95% ethanol faded to dark brown after 1.5 years of preservation.

***Head*** (Fig. 18B–D). Trapeziform, broad, round; densely setose anteriorly especially mouthparts. With 88–112 ommatidia (ocelli). Aberrant ommatidium situated at brim of antennal groove. Tömösváry’s organ separated from eye field and located between base of antenna and ommatidia. No sclerotised ridge between antennal groove and ommatidia.

***Antennae*** (Fig. 18B, C, E, F). Short and sturdy, emerging from both sides of head, covered by noticeable long setae; last antennomere reaches back posteriorly to leg pair 2. Lengths of antennomeres in descending order: 6 > 5 = 4 > 3 = 2 > 1. Antennomere 6 flattened, axe-shaped; with sensilla basiconica around apical disc. Apical disc shallowly concave; with 48–88 apical cones in males, and 43–77 apical cones in females.

***Epipharynx*** (Fig. 18K). Apically with a single obtuse central tooth (ct); in the middle with a conspicuous and swollen inner tooth; inner area with a single row of triangular fringed spines (rsp); outer area with several pointed and long external teeth (et).

***Gnathochilarium*** (Fig. 18G–J). Lamellae linguales (ll) rounded, apically concave, densely setose. Central pads (Cp) modified, with two types of sensory cones (sc), first small without a tubercle apically, second larger with a concave pad apically containing a single internal tubercle. Stipites (st) large and thick, with densely long setae; located laterally to lamellae linguales. Mentum (me) broad and fused, densely setose. Lateral palpi inconspicuous. Inner palpi (ip) with numerous cylinder sensory cones (sc) located in single field, each cone concave apically and containing a single internal tubercle.

***Mandibles* (*gnathal lobe*)** (Fig. 18L). With single undivided external tooth (Et) and 3-combed inner tooth (3it). With 6–7 rows of pectinate lamellae (pl). Inner area (Ia) with several rows of small, slender teeth. Molar plate (mp) relatively flat, with shallow fringe. Condylus (co) conspicuous; apically with two ridges.

***Tegument*** (Figs 17A–I, 19). Dull; collum, thoracic shield, tergites and anal shield densely covered by golden setae; each seta emerged from a pit. Tergites and anal shield sparsely setose anteriorly and densely setose posteriorly. Posterior rim with bare setae on both dorsal and ventral sides.

***Collum*** (Figs 18A). Subsemicircular; lateral edges triangular, with sparse setae.

***Thoracic shield***. Anterior margin with shallow canal covered by numerous long setae. Phitsanulok population with 3–6 small keels. Nakhon Sawan population with 6–10 conspicuous keels.

***Midbody tergite*** (Fig. 19A–C). Dorso-anterior margin with a row of circular impressions and numerous small tubercles. Inner area with a shallow groove, separated from anterior margin by a row of crenate barriers. Tips of paratergites projected posteroventrad.

***Anal shield*** (Fig. 19E–G). Slightly sexually dimorphic; female anal shield well-rounded, males rectangular and slender. Anteriorly with a row of circular impressions and numerous small tubercles. Inner area with a shallow groove; separated from margin by a row of crenate barriers. Underside with a dark locking carina; twice as long as those of tergites. Densely setose on both underside and upperside.

***Endotergum of thoracic shield and midbody tergite*** (Fig. 22G, H). Thoracic shield quite similar to midbody tergite. Posterior margin (pm) regularly flat. Outer area (oa) with several small denticles. Marginal bristles arranged in two rows, tips of longest bristles of Phitsanulok specimens two-thirds length of outer area (one-third in Nakhon Sawan specimens) and not projecting above margin. Middle area (ma) with a row of circular cuticular impressions (cp); distance between each impression twice as long as individual diameter in thoracic shield, and 3 × as long as individual diameter in midbody tergite. Cuticular impressions of thoracic shield shallower than those of midbody tergite. Inner area (ia) without tubercles or setae.

***Pleurite* (*laterotergite*)** (Fig. 19H, I). Densely setose. First pleurite boomeranglike with pointed edge, reaching towards paratergite. Pleurite 2 widened. The remaining flat, rectangular.

***Subanal plate of female*** (Fig. 19D). Densely setose; wide; semicircular; apical margin weakly concave, narrow.

***Stigmatic plates*** (Fig. 20A). First stigmatic plate quite slender, flat ante-posteriorly; apex broadly round, curved towards coxa. Second stigmatic plate in both sexes broad, weakly curved.

***Legs*** (Fig. 20). All podomeres densely setose. Coxa (cx) large, as long as length of prefemur (pre); first and second leg-pairs without coxal process; leg-pairs 3–21 with large and slightly serrated processes. Prefemur plump; with short ridge (rdg); apico-mesally with a very weak projection; mesal margin with one or two small triangular microscopic spines (sp). Femur (fe) prolonged; 1.5 × longer than prefemur; with conspicuous long ridge; apico-mesally with a strong projection; mesal margin dentated, with 10–12 small triangular spines. Postfemur (po) and tibia (ti) short. Tarsus (ta) of midbody legs long and slender, as long as length of femur+postfemur; first two leg-pairs without an apical spine; leg-pair 1 with two or three ventral spines; leg-pair 2 with four or five ventral spines; leg-pair 3 with 5–7 ventral spines and 1–3 apical spines; leg pair 4 with 7–10 ventral spines and three or four apical spines; leg pairs 5–19 with 9–12 ventral spines and 3–5 apical spines; leg pair 20 and 21 with 7–10 ventral spines and two or three apical spines. In leg 10, femur 1.5 ×, tarsus 3 × longer than wide. Claw normal; with notch at base.

***Male sexual characters*** (Fig. 20B). Gonopore surrounded by long setae; covered with a divided, triangular sclerotised plate. Apical part of coxa 2 without membranous process.

***Anterior telopods*** (Figs 21A, 21C, 21F, 21G, 23P–R). Consisting of four telopoditomeres. First telopoditomere broad; slightly longer than wide. Telopoditomere 2 large. Process of telopoditomere 2 (= immovable finger) broad; shorter than combination of telopoditomeres 3 and 4 (= movable finger); located posteriorly; clearly seen in posterior view; tapering apically; strongly curved toward movable finger; inner margin with a membranous lobe and numerous sclerotised spots (scl-s). Telopoditomere 3 slender; almost twice as long as telopoditomere 4; demarcated from telopoditomere 4 by weak suture; inner margin with single conspicuous spine; apically with 2–5 crenulated teeth (cr-t). Telopoditomere 4 very short; tapering tip, with two long spines. All telopoditomeres covered by long sparse setae, except for basal part of telopoditomere 1 and tip of telopoditomere 2.

***Posterior telopods*** (Figs 21A–E, 23S, 23T). Consisting of four telopoditomeres. First telopoditomere rectangular; longer than wide. Telopoditomere 2 large and stout. Process of telopoditomere 2 (= immovable finger) long and slender, 2.5 × longer than wide; slightly longer than a combination of telopoditomeres 3 and 4 (= movable finger); weakly tapering apical; slightly curved; inner margin with two membranous lobes (ml) and numerous conspicuous sclerotised spots (scl-s). Telopoditomere 3 long; 3 × longer than wide; with a large membranous ledge and single long spine at inner margin; posteriorly with a row of 12–17 crenulated teeth (cr-t). Telopoditomere 4 short; 4 × shorter than telopoditomere 3; twice as long as wide, slightly tapering apically; inner margin with two long spines located on membranous ledge. Telopoditomere 1 and 2 mostly covered by setae on anterior surface, glabrous on posterior surface. Telopoditomere 3 sparsely setose. Telopoditomere 4 glabrous. Inner horns sparsely setose; base wide; sharp-edged tips; slightly curved posteriorly.

***Female sexual characters*** (Fig. 20C). Vulva large, sparsely setose; covering almost half of coxa; located mesoventrally; extending mesally to base of prefemur. Operculum (op) slender; rectangular; with slightly concave mesal margin; tip of operculum dull. Bursa (bu) large; fused; separated from operculum by triangular groove. Apical part of coxa 2 without coxal process.

##### Remarks.

Notable sexual dimorphism is observed in the Phitsanulok specimens, in which adult males possess a greyish brown body with dark brown freckles, whereas adult females exhibit a reddish-brown body lacking freckles. Specimens from Nakhon Sawan Province differ in body colouration (brownish emerald-green) from those of Phitsanulok Province. The two populations also show slight morphological variation in marginal bristles of endotergum, the Phitsanulok specimens having bristles reaching two-thirds the length of the outer area, while those from Nakhon Sawan reach only one-third. These differences are accompanied by moderate but sub-specific genetic differentiation between the two populations (pairwise distances 5.02–5.63%), which remains well below the lowest interspecific distance recovered here (8.68%). Specimens from the type locality (Ban Nam Kob in Phitsanulok Province) were heavily infested with numerous small, white, phoretic deutonymphs of an unidentified mite, concentrated on the ventral surface, particularly legs (Fig. 20D, E).

##### Distribution and habitat.

*Zephronia triforma* sp. nov. is currently known only from the upper central region of Thailand, specifically Nakhon Sawan and Phitsanulok Provinces (Fig. 24). The specimens of populations from Phitsanulok Province were found during the daytime in dipterocarp forests, typically walking on the sandstone or sometimes hiding under the leaf litter (Fig. 17J). The individuals of the population from Nakhon Sawan Province were found in a dry deciduous forest characterised by sparse undergrowth, leaf litter, and rocky limestone terrain (Fig. 17K).

##### Etymology.

The species name is a Latin compound noun derived from *tri-* (three) and forma (form), referring to the three distinct morphotypes observed in the material used to describe the new species.

## Discussion

### Species delimitation and the barcoding gap in Thai *Zephronia*

An integrative approach combining both morphological and molecular information led to the discovery of two new giant pill-millipede species, viz., *Z. tamarindina* sp. nov. and *Z. triforma* sp. nov., and also the first records of *Z. dawydoffi* and *Z. laotica* in Thailand. All specimens of the latter two species closely match the original descriptions; however, morphological variations were observed and are documented here with SEM images. Both new species are assigned to the genus *Zephronia* based on the presence of diagnostic genus-specific characters, including axe-shaped antennae, the number of apical spines on the tarsi, and the distinctive morphology of the male telopods (Wesener 2016). Morphological and *COI* barcoding trees further indicate that the two new record species are closely related to *Z. siamensis* and *Z. ovalis*. These taxa share several diagnostic features, such as the presence of two colour patches on the tergites and a modified posterior margin of endotergum (Golovatch et al. 2012; Srisonchai et al. 2021; Srimongkol et al. 2026). In addition, the new species exhibits characters commonly observed in other congeners, including the position of the Tömösváry’s organ outside the antennal groove, a flat and regular posterior margin on the endotergum with 1–2 rows of marginal bristles, tips of the longest bristles not protruding over the posterior margin, and the absence of apical spines only on the first two leg pairs. Morphological and molecular evidence clearly place both species within *Zephronia* s.s. (Fig. 1). However, *Z. tamarindina* sp. nov. can be distinguished from its closely related congeners by the unique shape of anterior telopods (strongly curved in the process of telopoditomere 2 and widening telopoditomere 3, as well as a strongly curved telopoditomere 4 of posterior telopods, and a large membranous process on the coxa of male leg-pair 2. Although *Z. enghoffi*, *Z. golovatchi* and *Z. sukhothaiensis* also possess a similar membranous process in male leg pair 2 (Srisonchai et al. 2021; Srimongkol et al. 2026), *Z. enghoffi* and *Z. golovatchi* differ from *Z. tamarindina* sp. nov. in endotergal characters (bristle number and length), *Z. sukhothaiensis* differs in the length of process of telopoditomere 2 of posterior telopods, and the three species show genetic differences from *Z. tamarindina* sp. nov. (Fig. 1).

In *Zephronia*, species identification based solely on telopod morphology can be challenging, as interspecific variation in this structure is sometimes subtle and not always sufficient for clear species delimitation. Therefore, additional morphological characters, in combination with molecular evidence, are required to achieve reliable species identification.

*Zephronia triforma* sp. nov. is notable for its sexually dimorphic colouration within the Phitsanulok population, in which the males exhibit randomly distributed dark spots on a greyish-brown body, while females are reddish brown. This colour pattern differs markedly from that observed in both sexes of the Nakhon Sawan population, which display a uniform brownish emerald-green colouration and from all other known *Zephronia* species, with the exception of the spotted morph of *Z. macula*. Nevertheless, *Z. triforma* sp. nov. can be readily distinguished from *Z. macula* by several characters, including differences in live body colouration (*Z. macula* greenish to reddish brown vs. *Z. triforma* sp. nov. greyish brown), and clear genetic divergence (Srisonchai et al. 2024, Fig. 1).

In this study, DNA barcoding proved to be a highly effective tool for species delimitation (Hebert et al. 2003). In the case of the Phitsanulok population of *Z. triforma* sp. nov., barcoding reliably matched females with males of the same species and confirmed species identity across distinct morphotypes, including those from Nakhon Sawan and Phitsanulok populations. The *COI* barcoding sequences from these two populations formed two clearly separated subclades in both BI and ML trees, each with strong support (BS = 95, PP = 1.00). Despite this phylogenetic separation, the maximum intraspecific genetic distance between the populations was only 5.63%. A similar pattern has been reported for the green and spotted morphs of *Z. macula*, which differ markedly in body colour and patterns, yet exhibit a maximum intraspecific divergence of approximately 5% (Srimongkol et al. 2026). Likewise, Srisonchai et al. (2021) documented colour variation in *Z. phrain* relative to its type specimen, although no genetic divergence data between morphotypes were provided.

In our study, the *COI* genetic distance between geographic populations of *Z. triforma* sp. nov. (5.02–5.63%) approaches, but remains below, the minimum interspecific distance observed between *Z. siamensis* and *Z. dawydoffi* (8.68%). High intraspecific sequence divergence is relatively common in giant pill-millipedes, likely driven by low dispersal ability and prolonged geographic isolation (Wesener et al. 2010; Bhansali and Wesener 2022). Because the two *Z. triforma* sp. nov. populations display identical diagnostic characters in the structure of the male telopods, we treat this divergence as population-level. In contrast, *Z. dawydoffi* and *Z. siamensis* display distinct, non-overlapping differences in telopod morphology, confirming their separation despite the narrow barcoding gap. In giant pill-millipedes, interspecific genetic distances commonly used for species delimitation typically range from 8–21% (Donworth and Wesener 2024; Srisonchai et al. 2024). In the present study, the observed range was slightly broader (8.68–25.27%), most likely reflecting the inclusion of more closely related taxa. This pattern aligns with previous findings of *Zephronia* and related genera (Golovatch et al. 2012; Wesener 2019; Rosenmejer et al. 2021; Bhansali and Wesener 2022; Srisonchai et al. 2024; Srimongkol et al. 2026). However, the discovery of additional closely related species in future may further reduce the lower boundary of observed interspecific distances (Bhansali and Wesener 2022). We emphasise that species delimitation here is morphology-primary and *COI*-corroborative, no fixed genetic threshold was applied.

The relatively large interspecific genetic distances (8.68–24.89%) between the two newly described species, the two newly recorded species from Thailand, and their congeners (*Sphaerobelum* spp., *Prionobelum* spp., *Cryxus*, and *Zephronia* spp. from Southeast Asia) nonetheless highlight the evolutionary and biogeographical processes that have shaped diversity of Thai giant pill-millipedes (Wesener 2019). Despite this, interpretations of deep evolutionary relationships remain limited by reliance on a single-locus marker. Although the phylogenetic analyses placed all four species within the *Zephronia* s.s. group, resolution of internal nodes within *Zephronia* and *Sphaerobelum* was generally poor, and instances of non-monophyly were observed (Fig. 1). Weak support at deep nodes indicates that the current dataset is insufficient to fully resolve early divergences, a limitation also noted in previous studies (Hennen et al. 2022; Srisonchai et al. 2024; Srikampha et al. 2025). While recent phylogenetic relationships are well supported, deeper evolutionary splits remain uncertain and should be treated cautiously (Philippe et al. 2011).

### Generic limits within Zephroniinae

Molecular data based on the *COI* barcoding region show that traditional generic boundaries within Zephroniinae are problematic. Specifically, *Zephronia* is non-monophyletic, with species like *Z. hui*, *Z. medogensis*, and *Z. zhouae* nesting within *Sphaerobelum*. Together with the close placement of *Prionobelum* and *Cryxus*, these trees show that traditional diagnostic traits such as telopod morphology and the position of the Tömösváry's organ have evolved independently across different lineages or show greater structural variation than previously recognised. While single-locus *COI* data cannot formally revise the subfamily classification, they clearly demonstrate that morphology-based generic limits require thorough reconsideration. To establish a stable classification for Zephroniinae, future studies incorporating multi-locus markers, more conserved nuclear regions, and genomic-scale data combined with more comprehensive taxon sampling across Southeast Asia could improve the resolution within the genus or family (Wiens and Morrill 2011; Dietz et al. 2023). Pending such data, we retain the current generic assignments and refrain from proposing nomenclatural changes here.

### Distribution and biogeography

Based on the distribution record obtained in this study, *Z. tamarindina* sp. nov. and *Z. triforma* sp. nov. appear to be restricted to the upper central region of Thailand. *Z. tamarindina* sp. nov. is currently known only from its type locality in Phetchabun Province, whereas *Z. triforma* sp. nov. has been recorded exclusively from Nakhon Sawan and Phitsanulok Provinces. Consequently, both species are considered endemic to the upper central region. *Z. tamarindina* sp. nov. exhibits a narrow distribution, similar to several other Thai species that are confined to their type localities, such as *Z. chantaburiensis*, *Z. chrysomallos*, *Z. erawani*, and *Z. viridisoma* (Fig. 24). By contrast, *Z. triforma* sp. nov. shows a distributional pattern more comparable to widely distributed species, including *Z. macula*, *Z. panhai*, *Z. siamensis*, *Z. phrain*, and *Z. dawydoffi* (Fig. 24), some of which occur over areas spanning 200–300 km (Srisonchai et al. 2024). Both new species inhabit similar habitats and microhabitats.

*Zephronia dawydoffi*, originally described from southern Cambodia (Ream, Sre Umbell; Kampot, Kampong Speu) (Attems 1953; Wesener 2016), is here recorded for the first time from northeastern Thailand, including Buri Ram and Khon Kaen Provinces, extending its known distribution by approximately 300–700 km northward (Fig. 24). Within Thailand, the species appears to be relatively widespread in several districts of Buri Ram Province but was recorded from only a single locality in Khon Kaen Province. This distribution pattern suggests a relatively broad and semi-continuous range from southern and central Cambodia to the lower and central parts of northeastern Thailand. The distribution appears to be mainly continuous, but it is likely structured by modest geographical features such as low mountain ranges, the Phnom Dangrek or Daegrêk ranges, which may act as partial barriers to dispersal. Consistent with a pattern of isolation by distance, these conditions may promote gradual population differentiation across areas (Attwood et al. 2008; Siriwut et al. 2015). This interpretation is further supported by the observed *COI* genetic divergence between Cambodian and Thai populations of 3.65–5.18% (mean 4.03%), which indicates the early stages of population structuring across the range. No material is available from the ca. 150–700 km separating the Thai populations from the type localities, and the possibility of a morphological cline therefore remains untested. A similar distributional pattern has been observed in *Z. ovalis*, which also has a relatively broad and largely continuous range across mainland Southeast Asia (Srimongkol et al. 2026). It suggests that widely distributed *Zephronia* taxa share similar dispersal restrictions and landscape-mediated population structuring due to these similar distribution patterns of the two species.

*Zephronia laotica* was originally reported from Champasak Province in southern Laos, on the east bank of the Mekong River, and in this study it was recorded from Si Sa Ket Province in northeastern Thailand, approximately 150–200 km from its type locality (Fig. 24). Both *Z. dawydoffi* and *Z. laotica* occur primarily in agricultural habitats, especially rubber plantations with fine soil, where they are found under leaf litter and decayed wood. This matches Wesener’s (2019) observation of *Z. laotica* inhabiting hotel gardens as effectively man-made plantation forests. Comparable habitat associations have also been reported in other arthropod groups, highlighting the role of human-altered environments in shaping contemporary distribution patterns (Monaghan et al. 2009; Seibold et al. 2019; de Vries et al. 2026). Nevertheless, the habitat preference of this species remains unclear. Although it is frequently recorded from anthropogenic environments, it also occurs in natural habitats (K. Srikampha, unpublished data). This suggests that *Z. laotica* may not be restricted to human-modified landscapes but may have originally been distributed in natural ecosystems, with its apparent association with disturbed habitats potentially reflecting sampling bias or recent ecological expansion.

This study increases the number of recognised *Zephronia* species in Thailand to 18, expanding the genus’s known range, particularly in northeastern and upper central Thailand. The steady discovery of new giant pill-millipede species since 2021 (e.g., Likhitrakarn et al. 2021; Srisonchai et al. 2021; Donworth and Wesener 2024) highlights Thailand’s exceptional yet still underexplored biodiversity. Given that *Zephronia* is a species-rich genus, these findings suggest the existence of additional, as-yet-undescribed species, especially within the country’s unexplored natural habitats. With continued research and broader field surveys, *Zephronia* is poised to become not only one of the key groups representing the diversity of Thailand’s millipede fauna, but also an important component of the Southeast Asian biodiversity hotspot.

## Supplementary Material

XML Treatment for Zephronia

XML Treatment for Zephronia dawydoffi

XML Treatment for Zephronia laotica

XML Treatment for Zephronia tamarindina

XML Treatment for Zephronia triforma

## Additional information

### Artificial Intelligence (AI) use

The authors accept full responsibility for the content of the manuscript, including the disclosure of any use of AI.

No AI tools were used in the preparation of this manuscript.

### Author ORCIDs

C. Wilaisorn https://orcid.org/0009-0001-3673-4167

R. Srisonchai https://orcid.org/0000-0002-7142-0999

W. Kutanan https://orcid.org/0000-0001-7767-1644

N. Tungpairojwong https://orcid.org/0000-0001-8135-4246

### Data availability

The data underlying this article are available in the GenBank Nucleotide Database (https://www.ncbi.nlm.nih.gov/genbank/), and can be accessed with accession numbers listed in Table 1.

## Supplementary materials

Supplementary material 1 Supplementary information Data type xlsx Explanation note Genetic divergences of cytochrome c oxidase subunit I (*COI*) sequence (uncorrected *p*-distances) within and among *Zephronia* species, and related genera (*Sphaerobelum*, *Prionobelum*, and *Cryxus*). https://binary.pensoft.net/file/1796612This dataset is made available under the Open Database License (http://opendatacommons.org/licenses/odbl/1.0/). The Open Database License (ODbL) is a license agreement intended to allow users to freely share, modify, and use this Dataset while maintaining this same freedom for others, provided that the original source and author(s) are credited. Chalintorn Wilaisorn, Ruttapon Srisonchai, Wibhu Kutanan, Nisarat Tungpairojwong
